# Dual-locked strategy for fluorescence/chemiluminescence dual-modal imaging reprogramming

**DOI:** 10.1039/d5sc09065b

**Published:** 2026-03-06

**Authors:** Qi Wang, Zhao-Yao Song, Zhe Song, Guangji Wang, Le Zhen

**Affiliations:** a Key Laboratory of Drug Metabolism and Pharmacokinetics, China Pharmaceutical University Nanjing 210009 Jiangsu China i_m_zhenle@163.com; b Instrumental Analysis Center, China Pharmaceutical University Nanjing 210009 Jiangsu China

## Abstract

Fluorescence (FL) and chemiluminescence (CL) each offer unique advantages for molecular imaging. FL allows for continuous and real-time tracking of analytes, while CL provides an ultra-high signal-to-background ratio by eliminating the need for exogenous excitation light. Integrating these two modalities for imaging presents significant synergies as well as challenges. However, the rarely reported FL/CL dual-modal probes are typically limited to a “CL–FL” mode, in which both FL and CL signals are activated simultaneously, with the transient CL signals being captured instantly and processed preferentially. In this study, we designed a novel near-infrared FL/CL dual-modal probe that introduces an innovative “FL-trigger-CL” mode for imaging. This approach employs a dual-locked strategy, allowing for flexible and time-controlled triggering of the CRET-based redshift CL signal dependent on fluoride-ion-mediated desilylation, a biocompatible bioorthogonal chemistry. The probe, named FTC-01, was successfully applied to detect carbon monoxide, an endogenous inflammatory biomarker, in both cells and mice with colitis, incorporating FL for primary detection and CL for accurate in-depth detection. This advancement is expected to enhance the application of this dual-modal imaging logic in disease diagnosis and drug candidate screening. Furthermore, our dual-locked probe platform demonstrates excellent compatibility and the potential for expansion, facilitating the integration of a broader range of sensing units and guiding the rational design of dual-modal probes in the future.

## Abstract 2

A dual-locked probe enables sequential “FL-trigger-CL” imaging, allowing on-demand activation of near-infrared chemiluminescence to validate carbon monoxide elevation in a live colitis model.

## Graphical abstract

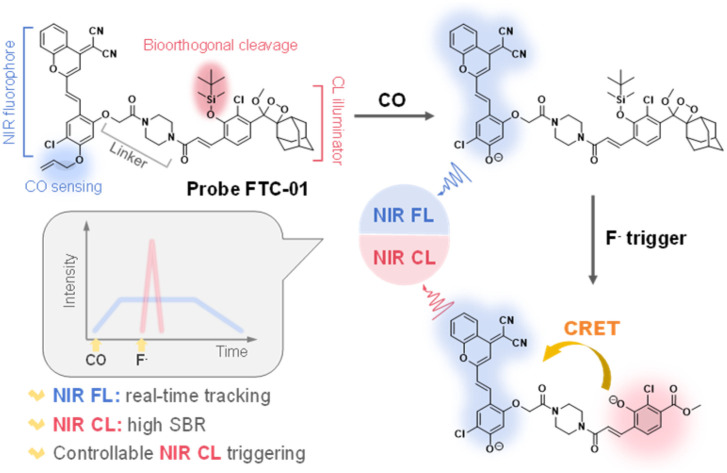

## Introduction

Activatable small-molecule optical probes have emerged as powerful tools for disease diagnosis and bioanalysis, owing to their ability to selectively interact with biomarkers and generate spatiotemporally resolved signals.^1,2^ Among these probes, chemiluminescence (CL)^3,4^ and fluorescence (FL)^5,6^ imaging modalities stand out as complementary techniques: FL enables real-time tracking of biomolecules with high temporal resolution, while CL eliminates photobleaching and background interference through external light-free excitation, achieving ultrahigh signal-to-background ratios (SBRs).^7,8^ Integrating both modalities into a FL/CL dual-modal unimolecular platform could synergistically combine their strengths for sensitive, real-time, and high-precision bioimaging.^9–12^ However, recent advances in near-infrared (NIR, 700–1700 nm) imaging complicate the integration of NIR FL and NIR CL. Both methods impose stringent and often distinct requirements on molecular design, including extended π-conjugation, optimized donor–acceptor alignment, and controlled energy transfer. This makes it challenging to harmonize the two emission mechanisms within a single probe.

**Fig. 1:**
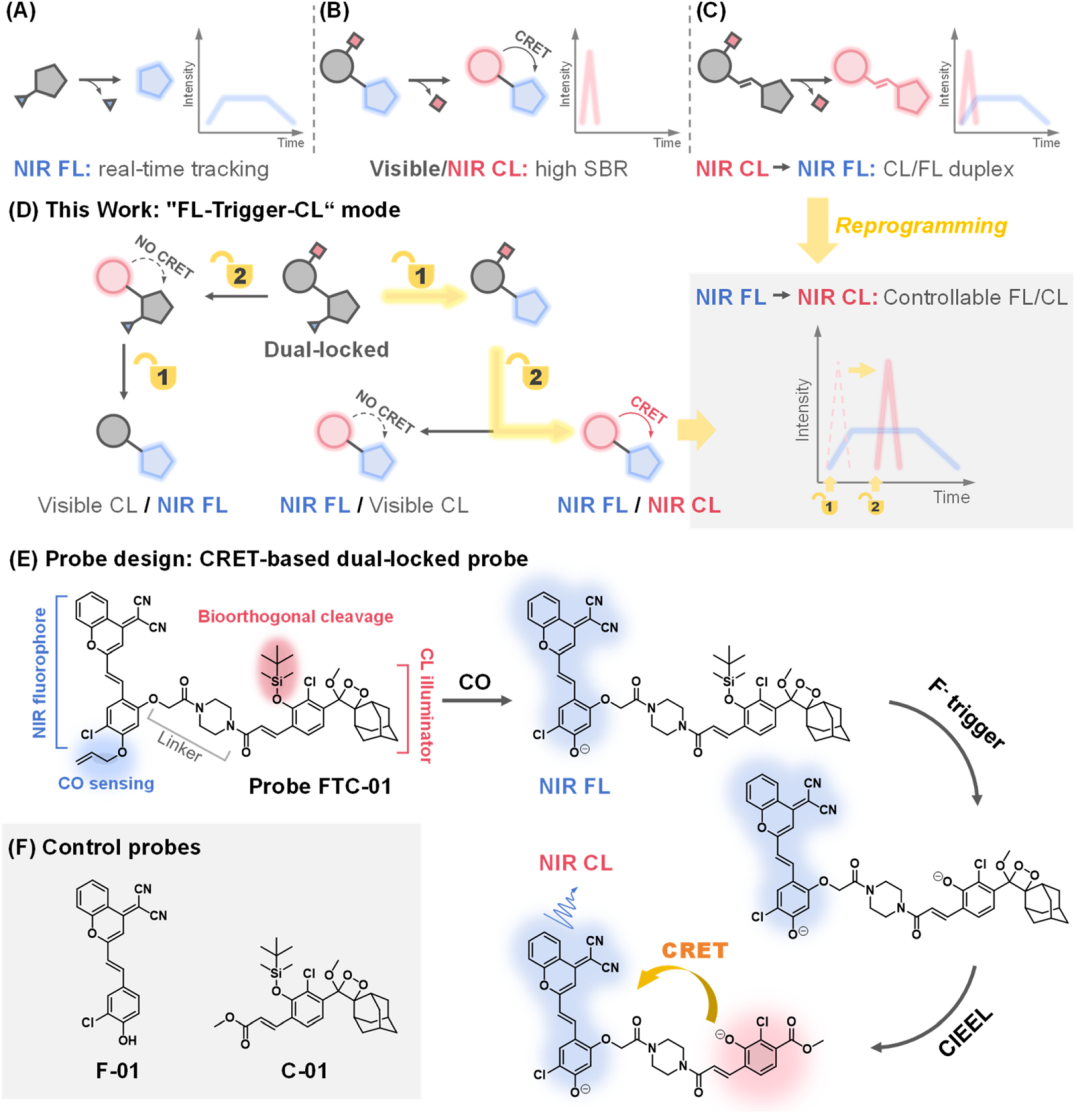
Design of the CRET-based dual-locking probe FTC-01. (A–C) Schematic illustration of the output modes and kinetic profiles of NIR FL (A) and NIR CL (B is CRET-based NIR CL; C is conjugate-based bimodal NIR CL/NIR FL). (D) Schematic illustration of the CRET-based dual-locked probe with “FL-trigger-CL” mode. (E) Chemical structure of FTC-01 and its activated forms in response to CO and F^−^. (F) Design of control probes F-01 and C-01.

NIR fluorophores in both NIR-I (700–900 nm) and NIR-II (1000–1700 nm) windows offer real-time tracking, deeper tissue penetration and reduced scattering (Fig. 1A).^2,13,14^ Recently, the emission wavelength of Schaap's dioxetane,^15–20^ a well-regarded illuminator for constructing small-molecule CL probes, has been gradually transitioning from the visible spectrum (400–650 nm) into the NIR range. Current strategies to achieve NIR CL imaging either rely on chemiluminescence resonance energy transfer (CRET)-mediated wavelength shifting (Fig. 1B and Table S1)^21–28^ or the extension of π-conjugation in dioxetanes (Fig. 1C).^12,29–32^ For example, Song *et al.* recently developed an H_2_S-activated unimolecular probe, CD-950, which achieved CL in the NIR-II region through efficient CRET, exhibiting bright signals at 950 nm that are specifically activated by H_2_S, with a high SBR.^28^ Nevertheless, this type of probe presents a limitation: the fluorophore moiety lacks analyte-responsive “off–on” switching due to its structural independence from the activatable group of the probe, precluding dual-channel specificity. In contrast, dioxetane systems with extended π-conjugation emit NIR CL directly and produce metabolites that enable FL imaging. For instance, Song *et al.* recently constructed NIR-II CL unimolecular probes featuring a multiconjugated double-bond structure.^12^ The metabolites produced by the probes after chemiexcitation could also be excited by using a laser, allowing for simultaneous CL/FL imaging of superoxide anions in mice liver injury. Given the transient nature of CL signals, which last for around 10 minutes as reported for representative direct NIR-CL probes,^12^ it is crucial to prioritize timely capture of these signals within this time window to prevent signal loss.

In this work, we propose a novel dual-locked molecular design^33^ that addresses the above challenges through two key innovations: (1) independently caging the CL and FL units with distinct biochemical triggers, enabling sequential activation; (2) reprogramming the traditional imaging paradigm to “FL-trigger-CL” mode, where FL signals facilitate real-time monitoring, followed by on-demand CL activation using an externally added “trigger” (in this case, F^−^) for high-SBR validation. As illustrated in the schematic (Fig. 1D), this dual-locked probe emits NIR FL/NIR CL signals only when two conditions are met: the availability of CRET and the sequential uncaging of the probes. By regulating the timing of the second trigger-mediated uncaging, the initiation of CRET can be controlled, resulting in “FL-trigger-CL” mode imaging.

As a proof of concept, we applied this strategy to the detection of carbon monoxide (CO) – a gaseous signaling molecule implicated in inflammation and oxidative stress regulation.^34,35^ While fluorescent probes have advanced CO bioimaging,^36–41^ CL-based detection remains limited to buffer systems.^42^ Our dual-locked probe, FTC-01, uniquely integrates a CO-responsive NIR fluorophore and a fluoride-ion-sensitive CL emitter based on Schaap's dioxetane, which can be bioorthogonally uncaged to initiate chemically initiated electron exchange luminescence (CIEEL),^43^ and subsequent CRET (Fig. 1E). This design achieves controlled sequential NIR FL-to-NIR CL imaging *in vivo*, successfully tracking CO elevation in a DSS-induced colitis model. To our knowledge, this represents the first CRET-based dual-locked probe with independent analyte recognition, the first demonstration of endogenous CO detection *via* CL imaging, and a new dual-channel framework that redefines multimodal imaging logic.

## Results and discussion

### Probe design

The dual-locked probe FTC-01 consists of three functional components: a NIR fluorophore, a CL illuminator, and a linker (Fig. 1E). Our initial development focused on the CO-responsive NIR FL fluorophore. Previous studies have shown that CO can reduce Pd(ii) to Pd(0), which acts as a catalyst in the Tsuji–Trost reaction to remove allyl groups.^40–42,44,45^ The allyl-caged phenolic hydroxyl group enables CO-specific activation *via* deallylation. A dicyanomethylene-4*H*-benzopyran derivative F-01 (Fig. 1F) was selected and modified with chlorine atoms to increase the p*K*_a_ of the phenolic hydroxyl group, facilitating effective intramolecular charge transfer (ICT) under physiological pH conditions.^46^ Next, we designed Schaap's dioxetane-based CL probe C-01 (Fig. 1F), which carried a *tert*-butyldimethylsilane (TBS) moiety, offering biocompatibility advantages^47–51^ including bioorthogonal excision, high sensitivity to fluoride ions (F^−^), and ease of synthesis.

**Fig. 2:**
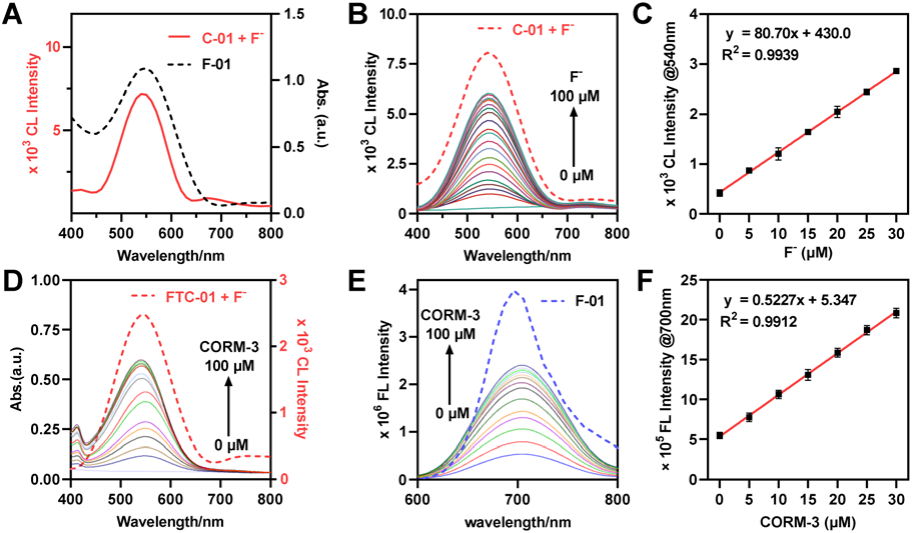
Characterization of FTC-01*in vitro*. (A) CL spectrum of C-01 (10 µM) + NaF (20 µM); control: the absorbance spectrum of F-01 (10 µM). (B) CL spectra of FTC-01 (10 µM) in the presence of NaF (0–100 µM); control: the CL spectrum of C-01 (10 µM) + NaF (100 µM). (C) The linear relationship between FTC-01 (10 µM) and NaF concentration (0 to 30 µM). (D) Absorption spectra of FTC-01 (10 µM) + PdCl_2_ (10 µM) in the presence of CORM-3 (0–100 µM); control: the CL spectrum of FTC-01 (10 µM) + NaF (20 µM). (E) FL spectra of FTC-01 (10 µM) in the presence of CORM-3 (0–100 µM); control: the FL spectrum of F-01 (10 µM). *λ*_ex_ = 540 nm. (F) The linear relationship between FTC-01 (10 µM) + PdCl_2_ (10 µM) and CORM-3 concentration (0 to 30 µM). All the measurements were performed in PBS (10 mM, pH = 7.4, 50% DMSO) at 37 °C. The LOD was calculated using 3*σ*/*k*, where *σ* = 0.466. Data as mean values ± SD (*n* = 5).

To achieve effective CRET, it is essential to maximize the overlap between the CL emission spectrum of the donor and the absorption spectrum of the acceptor.^21–28,52^ Additionally, the spatial arrangement of the donor and acceptor molecules must be appropriately configured.^53^ We found that the CL emission spectrum of probe C-01 significantly overlaps with the absorption spectrum of F-01 (Fig. 2A). This overlap supports our selection of probes C-01 and F-01 as the donor and acceptor for CRET, respectively. Furthermore, we modified probe F-01 by introducing another phenolic hydroxyl group at the para-position of the chlorine atom, which serves as a ‘handle’ for coupling to the CL donor. To connect the CL donor to the fluorophore while maintaining some rigidity, we utilized a piperazine-containing linker. Based on these modifications, we designed and synthesized probe FTC-01, along with control probes C-01 and F-01 (Schemes S1–S3), to validate the CRET process. In summary, the selection of CO and F^−^ as the keys for the dual-lock system follows a clear imaging logic: the endogenous inflammation-related molecule CO first triggers NIR fluorescence for preliminary screening, after which the exogenous, highly selective bioorthogonal stimulus F^−^ is employed to turn on NIR chemiluminescence on-demand for precise verification. The two response mechanisms guarantee strict sequential signal activation, thereby enabling a fundamental shift from the conventional “CL–FL” mode to a controllable “FL-trigger-CL” paradigm.

### Probe response to F^−^ and CO

The optical properties of FTC-01 and its response to F^−^ and CO were initially evaluated. The reaction between FTC-01 and F^−^ resulted in the removal of the TBS group, producing a bright chemiluminescent signal with a maximum emission wavelength of 540 nm (Fig. 2B). The resulting CL spectrum closely matched that of the control probe C-01 (Fig. S1A) and exhibited a concentration-dependent enhancement in response to F^−^. The CL intensity showed a linear correlation over a wide range, with a high correlation coefficient and a low limit of detection (LOD) (*R*^2^ = 0.9939, *y* = 80.70*x* + 430; LOD = 8.95 nM) (Fig. 2C). Subsequently, the absorption and fluorescence spectra of the probes were analyzed. After the addition of CORM-3 (a CO donor) and PdCl_2_, both FTC-01 and F-01 displayed nearly identical absorption and emission spectra, with a maximum absorption peak near 540 nm and a strong NIR fluorescence signal at around 700 nm (Fig. S1B). Notably, the absorption spectrum closely aligned with the CL spectrum of FTC-01 described above, supporting the facilitation of the CRET process (Fig. 2D). The fluorescence titration curve of FTC-01 increased gradually with increasing CO concentration (Fig. 2E) and demonstrated a strong linear relationship (Fig. 2F), yielding a correlation coefficient *R*^2^ of 0.9912. The LOD for the CO donor was determined to be 2.674 µM. These experiments indicated that FTC-01 are appropriately designed to retain the same optical properties as the parent probes (C-01 and F-01), which is conducive to realizing CRET.

### *In vitro* CRET

**Fig. 3:**
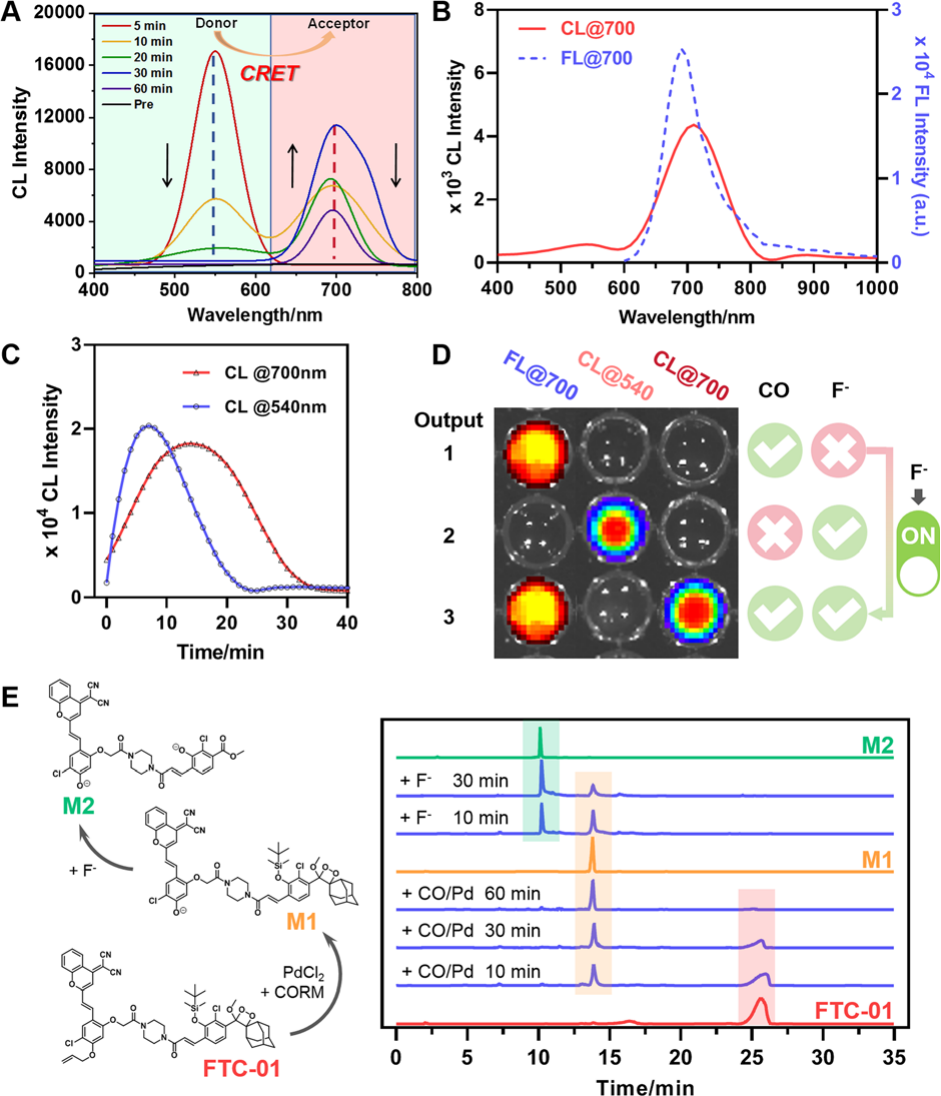
Confirmation of controllable CRET initiation. (A) Kinetic profiles of CL spectra for FTC-01 during CRET under trigger condition 1: FTC-01 (100 µM)/PdCl_2_ (100 µM)/CORM-3 (200 µM)/NaF (200 µM). (B) FL spectrum of F-01 (10 µM, *λ*_ex_ = 540 nm) and CL spectrum of FTC-01 (10 µM) under trigger condition 2: FTC-01 (100 µM)/PdCl_2_ (10 µM)/CORM-3 (50 µM) for 30 min, and then NaF (50 µM). (C) Kinetic profile of CL@540 after treating FTC-01 (10 µM) with NaF (50 µM) *versus* that of CL@700 after treating FTC-01 (10 µM) under trigger condition 2. CL kinetics of FTC-01 (10 µM) at the CL@540 channel produced by treatment with NaF (50 µM) only *versus* CL@700 channel produced by treatment under trigger condition 2. (D) Schematic diagram of FTC-01 (10 µM) output signals in different activators and different signal channels. (E) The unlocking mechanism of FTC-01. FTC-01 (2 mM), PdCl_2_ (2 mM), and CORM-3 (4 mM) were incubated for 60 minutes, followed by the addition of NaF (2 mM) aqueous solution for 30 minutes. All the measurements were performed in PBS (10 mM, pH = 7.4, 50% DMSO) at 37 °C. Data as mean values ± SD (*n* = 5).

Subsequently, it was confirmed that FTC-01 could achieve CRET through the following key findings: (1) change of CL wavelengths: under trigger condition 1, which involves simultaneous application of F^−^ and CORM-3/PdCl_2_ for unlocking, the coexistence of CL signals at two wavelengths (CL@540 and CL@700) was observed (Fig. 3A). During the first 30 min, the intensity of CL@540 gradually decreased while that of CL@700 steadily increased, reaching its peak at 30 minutes. At 60 minutes, CL@540 completely disappeared, and CL@700 dropped to half its intensity compared to the 30 minute reading. For trigger condition 2, which entailed pretreating with PdCl_2_/CORM-3 for 30 minutes prior to triggering with fluoride ions (F^−^), the maximum emission wavelength of the CL signal presented by FTC-01 red-shifted to 700 nm. The CL emission spectrum closely resembled that of FL@700 from F-01 (Fig. 3B and S1B). Notably, the CL signal was amplified approximately 40-fold relative to the background signal. (2) Changes in the CL kinetics: when F^−^ were used as the solo trigger, the intensity of CL@540 peaked between 5 and 8 minutes and completely disappeared by 20 min (Fig. 3C). However, when both locks were activated under trigger condition 2, the peak intensity of CL@700 was delayed until 15 min and persisted until 37 min before it finally disappeared. Consistent with our probe design, F^−^ alone are not expected to initiate CRET, whereas trigger condition 2 effectively induces it. The observed shift in the luminescence peak timing across trigger conditions suggests that delayed CL kinetics can be attributed to the CRET process. Early signs of this delay are evident in Fig. 3A, where CL@540 appears first, followed by a gradual increase in CL@700.

Notably, FTC-01 was capable of generating three different signals based on CRET: FL@700, CL@540, and a dual-modal FL/CL@700, which correspond to the presence of three specified analytes: CO, F^−^, and CO + F^−^, respectively (Fig. 3D and S3). We believe that this system offers a primary advantage: when CO was detected using the NIR-FL channel, the CRET process could be initiated by introducing F^−^ as a trigger, which generated an additional CL signal. This strategy allows us to implement a controlled CRET process that pops up the CL signal with an appropriate trigger during the initial NIR-FL-based screening. We refer to this process as the “FL-trigger-CL” mode. This mode reprograms the imaging sequence of FL and CL, allowing for better synchronization between the long-duration imaging of FL and the precise imaging of CL, while overcoming the short time window for capturing CL signals using conventional CL probes as well as the emerging CRET-based CL probes. Additionally, the CRET efficiency of FTC-01 was calculated to exceed 93%, based on the ratio of the area under the curve of CL@700 to that of CL@540 (Fig. S2). Furthermore, the structural confirmation of the intermediate (M1 and M2) generated by FTC-01 upon activation provides direct evidence supporting the proposed reaction mechanism (Fig. 3E, Schemes S4 and S5). Together, these findings demonstrate the capability of FTC-01 to achieve CRET under specific dual activation conditions.

### Stability and selectivity

**Fig. 4:**
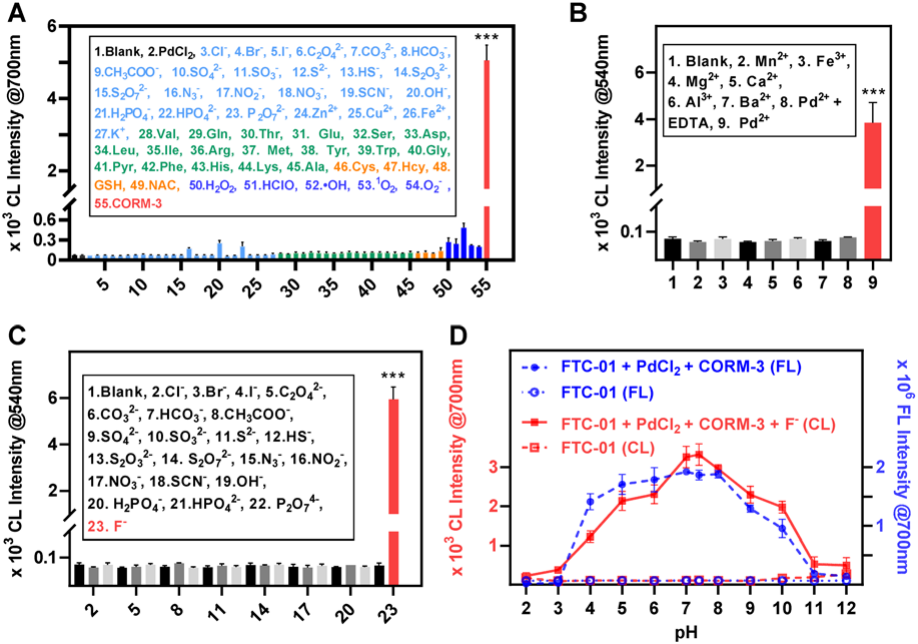
(A) NIR-CL intensity of FTC-01 (10 µM) at 700 nm after treatment with PdCl_2_ (10 µM) and biological analytes (100 µM; 3–27: inorganic ions; 28–45: amino acids; 46–49: thiol; 50–54: ROS/RNS; 55: CORM-3) for 30 min, followed by addition of F^−^ (20 µM). (B) NIR-CL intensity of FTC-01 (10 µM) at 700 nm after treatment with metal ions (10 µM) and CORM-3 (100 µM) for 30 min, followed by addition of F^−^ (20 µM). (C) CL intensity of FTC-01 (10 µM) at 540 nm in the presence of F^−^ (20 µM) or other analytes (100 µM). (D) NIR CL/FL intensity of FTC-01 (10 µM) after treatment with PdCl_2_ (10 µM) and CORM-3 (100 µM) for 30 min in PBS (10 mM, 50% DMSO) at different pH values, followed by addition of F^−^ (20 µM). All the measurements were performed in PBS (10 mM, 50% DMSO) at 37 °C. Data as mean values ± SD (*n* = 5); ***P < 0.001.

The effects of biological interferences on the cleavage at the reaction site and the CRET process of FTC-01 were evaluated. First, it was determined that biological analytes (such as potentially interfering ions, amino acids, thiols, and ROS) had little effect on the CRET process. In the presence of F^−^, bright CL@700 signals were produced exclusively when CO coexisted with PdCl_2_ (Fig. 4A and B). Next, the reaction specificity of fluoride ions in removing the TBS group from FTC-01 was confirmed by monitoring CL signals after adding anions (such as halogens, carbonate, sulfate, nitrate, phosphate, *etc.*) to the buffer system containing FTC-01. The CL signals significantly increased only in the presence of F^−^, while signals from other anions were nearly undetectable (Fig. 4C). The impact of pH values on the response of FTC-01 was also investigated (Fig. 4D). Across a wide range of pH values from 2 to 12, FTC-01 demonstrated a very low background in both FL and CL channels. Between pH 5 and 9, FTC-01 yielded satisfactory FL@700 and CRET signals with signal-to-background ratios (SBRs) exceeding 20. In the optimum pH range of 7 to 8, SBR values reached 30 or higher.

### Cellular CO imaging

**Fig. 5:**
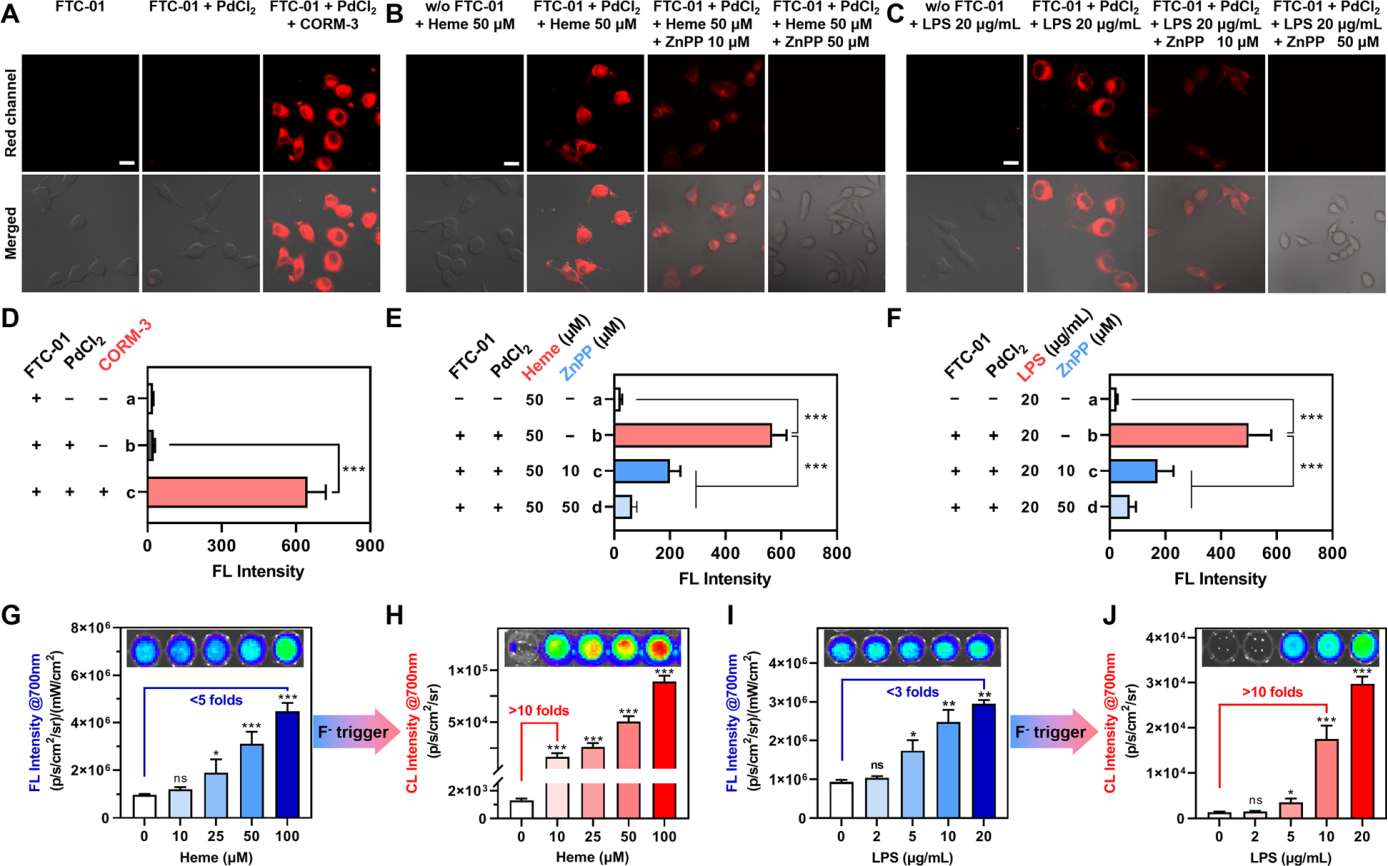
Detection of cellular CO. (A) Confocal fluorescence images of HeLa cells pretreated with CORM-3 (100 µM) for 30 min and then incubated with FTC-01 (10 µM) + PdCl_2_ (10 µM) for 30 min. (B and C) Confocal fluorescence images of HeLa cells pretreated with Heme (50 µM) or LPS (20 µm mL^−1^) for 12 h, with subsequent inhibitor ZnPP treatment in selected groups, before incubation with FTC-01 (10 µM) + PdCl_2_ (10 µM) for 30 min. Red channel: *λ*_ex_ = 555 nm, *λ*_em_ = 685–760 nm. Scale bar = 20 µm. (D–F) Quantification of the FL intensities in (A–C) respectively. (G–J) FL@700 and CL@700 intensities of HeLa cells. HeLa cells were induced with different concentrations of Heme (G and H) or LPS (I and J) for 12 h and then treated with FTC-01 (10 µM) and PdCl_2_ (10 µM) for 30 min, followed by NaF (50 µM) treatment. Inset: FL/CL images of HeLa cells. All the signals were obtained in the fluorescence or bioluminescence mode of an IVIS Lumina XR III system, and the corresponding wavelength emission signals were collected through filters. *λ*_ex_ = 540 nm, *λ*_em_ = 700 nm. Data as mean values ± SD (*n* = 6); ns: not significant, *P < 0.05, **P < 0.01, ***P < 0.001.

The application of FTC-01 for detecting and imaging intracellular CO was validated. Initially, FTC-01 exhibited low cytotoxicity, with >80% cell viability in HeLa and HepG2 cells at concentrations up to 40 µM (CCK-8 assay, 24 h) (Fig. S4 and S5). Subsequently, we treated the cells with PdCl_2_ and the CO donor CORM-3 in combination with FTC-01 (Fig. 5A and S6A). The probe exhibited minimal fluorescence with FTC-01 alone or FTC-01 combined with PdCl_2_, suggesting a low detectable intracellular CO content. However, when PdCl_2_ was co-expressed with CORM-3, FTC-01 produced NIR-FL signals within cells. Specifically, the signal intensity increased 50-fold in HeLa cells (Fig. 5A and D) and 10-fold in HepG2 cells (Fig. S6). This indicates that FTC-01 can be intracellularly activated to emit NIR-FL signals by CO.

Heme oxygenase (HO) is responsible for metabolizing hemoglobin (Heme), which is the primary pathway for intracellular CO production.^35,54^ It has also been shown that lipopolysaccharide (LPS) can increase cellular HO-1 expression.^55,56^ In light of these findings, this study aims to investigate the effects of Heme and LPS on HO-mediated endogenous CO production using FTC-01 (Fig. 5B and C). HeLa cells were pre-treated with Heme or LPS for 12 hours, after which intracellular CO levels were analyzed using confocal imaging. Heme or LPS treatment markedly enhanced red fluorescence, an effect abolished by the HO-1 inhibitor zinc protoporphyrin IX (ZnPP) (Fig. 5E and F). Consistent results were also observed in HepG2 cells (Fig. S7). These results demonstrate that FTC-01 is a reliable imaging probe for measuring intracellular CO levels and indicate that both Heme and LPS increase intracellular CO levels.

### Cellular CRET

Dual-channel detection (CL@700 and FL@700) revealed significant amplification of the CO-dependent signal from FTC-01 (Fig. 5G–J): under the FL@700 channel, we observed that increasing concentrations of Heme or LPS resulted in a substantial increase in the signal. The maximum FL@700 intensities demonstrated an increase of 4.8-fold for Heme (100 µM) and 2.9-fold for LPS (20 µg mL^−1^), compared to untreated controls (Fig. 5G and I).

Fluoride-triggered CRET was subsequently achieved through monitoring of CL@700. Before this, the optimal concentration of the fluoride trigger (F^−^) was determined to be 50 µM (Fig. S8). The CL response displayed a positive correlation with the inducer concentrations, resulting in notable signal amplification. Specifically, treatment with low-dose Heme (10 µM) and mid-concentration LPS (10 µg mL^−1^) both generated over a 10-fold enhancement in the CL@700 signal compared to baseline levels (Fig. 5H and J). Single-component systems containing either PdCl_2_ or CORM-3 failed to initiate CRET upon F^−^ exposure, as evidenced by CL@540 signal detection (Fig. S9A and B). However, when PdCl_2_ and CORM-3 were co-administered, fluoride-triggered CRET activation was achieved, as indicated by a complete signal transition from the CL@540 to the CL@700 channel (Fig. S9C and D). These results demonstrate that (1) both Heme and LPS effectively induce intracellular CO generation, with Heme having a more pronounced effect; (2) the probe FTC-01 successfully recognizes and images endogenous CO; and (3) a cellular “FL-trigger-CL” mode of FTC-01 was validated, in which F^−^ enables a precise switch in signal modes by triggering CRET, with CL@700 showing superior sensitivity for detecting CO compared to FL@700-based methods.

### *In vivo* imaging

**Fig. 6:**
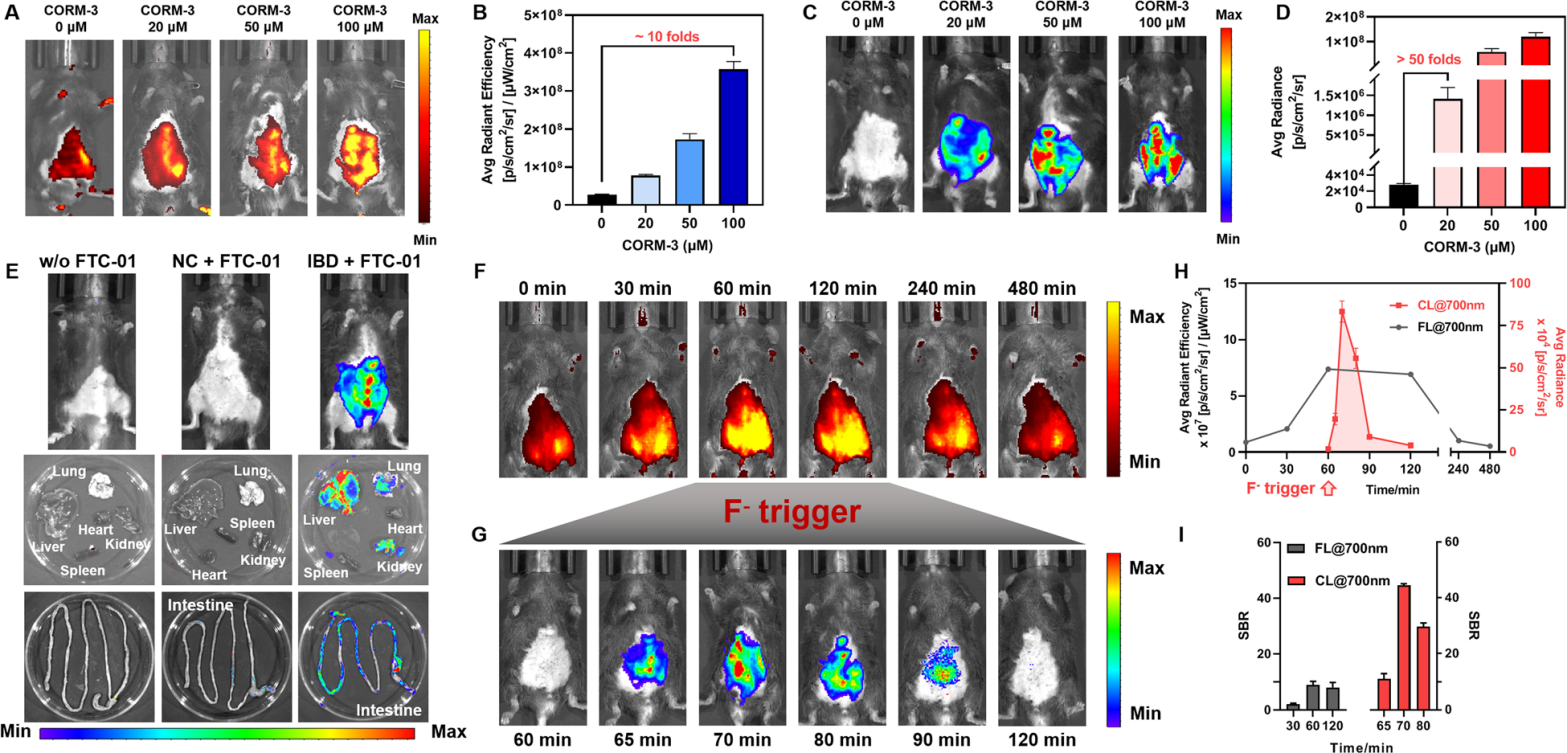
*In vivo* CO profiling. (A–D) NIR FL/NIR CL dual-channel *in vivo* imaging of CO released by CORM-3. FL@700 images (A) and quantification (B) of C57 mice treated with FTC-01 (200 µM, 25% v/v DMSO, i.p.)/PdCl_2_ (200 µM, i.v.)/different concentrations of CORM-3 (10% v/v DMSO, i.v.) for 30 min. CL@700 images (C) and quantification (D) after injection of NaF (1 mM, i.v.) for 30 min. (E–I) NIR FL/NIR CL dual-channel *in vivo* imaging of CO in IBD mice and organs. FL@700 (E and F) and CL@700 (G) images after sequential injection of PdCl_2_ (200 µM, i.v.), FTC-01 (200 µM, 25% v/v DMSO, i.p.), and NaF (1 mM, i.v.) at 30 minute intervals in the vehicle group (w/o FTC-01), normal control (NC) or IBD mice. Quantification (H) and signal-to-background ratio (SBR) (I) of FL@700 and CL@700 signals in F and G. All injections were dissolved in 200 µL saline each time. All the signals were obtained in the bioluminescence mode of the IVIS Lumina XR III system. *λ*_ex_ = 540 nm, *λ*_em_ = 700 nm. Data as mean values ± SD (*n* = 5).

FTC-01 effectively detects intracellular CO. Following this, FL/CL dual-modal CO detection and imaging were performed in mice. An initial biocompatibility assessment of FTC-01 indicated no acute toxicity, as evidenced by stable body weight progression (*Δ*weight < 5%) and a 100% survival rate in mice, further supported by the absence of notable histopathological damage in vital organs (Fig. S10 and S11). Additionally, FTC-01 enabled effective detection of CO, demonstrating deep-tissue penetration capability up to 12 mm, as validated in a bacon model (Fig. S13). For CO detection, the mice underwent sequential treatments: (1) PdCl_2_ (200 µM, i.v.) + gradient CORM-3 concentrations (0–100 µM, i.v.) for 30 min; (2) FTC-01 (200 µM, i.p.) for 30 min followed by FL detection; and (3) NaF (1 mM, i.v.) for CRET activation and CL detection. Imaging *via* FL@700 at 30 min post-probe injection demonstrated a dose-dependent enhancement in signal intensity, achieving a maximum ratio of 10.2 (Fig. 6A and B). Remarkably, subsequent CL@700 detection revealed a signal-to-background enhancement of up to 50-fold (Fig. 6C and D), even at sub-micromolar concentrations of CORM-3 (20 µM), significantly outperforming the FL@700 modality. These results suggest that FTC-01 is a promising tool for *in vivo* imaging of CO and can facilitate controllable CRET, allowing for more sensitive detection in living animals.

*In vivo* CO profiling in colitis models: to evaluate the diagnostic potential of FTC-01 under pathological conditions, we established an inflammatory bowel disease (IBD) model induced by dextran sulfate sodium (DSS) (4% DSS). The colitis phenotype was validated through several assessments: (1) progressive body weight loss (*Δ*weight > 20% *vs.* control, Fig. S10A); (2) an elevated disease activity index (Fig. S10B); (3) colon shortening (4.5 ± 0.5 cm in IBD mice *vs.* 8.4 ± 0.3 cm in healthy mice, Fig. S10C), and (4) histopathological confirmation of epithelial barrier disruption (Fig. S12). Endogenous CO levels were then measured following sequential injections of PdCl_2_ (200 µM, i.v.) and FTC-01 (200 µM, i.p.) at 60 min intervals, followed by NaF administration (1 mM, i.v.). The abdomens of IBD mice showed bright signals in the CL@700 channels, which were absent in normal mice. This indicates an upregulation of endogenous CO in DSS-induced intestinal inflammation (Fig. 6E). Furthermore, imaging of major organs (heart, liver, kidneys, spleen, lungs, and intestines) revealed that the intestines, particularly the colon, of IBD mice exhibited a distinct CL@700 signal (Fig. 6E). There was also a distribution of CO in the liver and kidneys. These findings align with previous literature, indicating that DSS impacts the physiological functions of the liver^56^ and kidneys,^57^ alongside causing intestinal injury. Overall, these results demonstrate that FTC-01 can be effectively used for the *in vivo* detection and imaging of CO in various organs. This capability for CO profiling positions FTC-01 as the first valuable tool for investigating the role of CO in inflammatory diseases.

### *In vivo* controllable CRET

The *in vivo* “FL-trigger-CL” mode of FTC-01 was validated using real-time multimodal imaging. Initially, PdCl_2_ (200 µM, i.v.) was injected into IBD mice, followed by the administration of FTC-01 (200 µM, i.p.) 30 minutes later. Over time, a gradual increase in the abdominal FL@700 signal was observed, reaching a plateau between 60 and 120 minutes before slowly diminishing until it nearly disappeared at 240 minutes (Fig. 6F and H). The duration of the FL@700 signal was sufficient, with a signal-to-background ratio (SBR) of approximately 8 (Fig. 6I), which is favorable for outlining the lesion site and determining the optimal detection time. Based on these observations, we initiated the CRET at 60 minutes (Fig. 6G). A CL@700 signal was detected 5 minutes after the F^−^ injection (1 mM, i.v.) at 65 minutes, indicating the activation of the CRET process. The CL@700 intensity peaked at 70 minutes and then gradually decreased over the next 20 minutes (Fig. 6H). Notably, the CL@700 signal exhibited superior SBR values compared to the FL@700 signal, with peak intensity reaching as high as 46 (Fig. 6I). These findings suggest that FTC-01 facilitates a controllable CRET process *in vivo*, effectively combining FL and CL signals.

## Conclusions

In summary, this study has designed and synthesized a unique double-locked unimolecule probe, FTC-01, to develop novel bimodal molecular imaging tools that integrate both FL and CL signals. The probe allows for the sequential activation of FL and CL signals: the NIR FL signals are initially activated through a biomolecular sensor, while the time-controllable activation of CL signals is achieved through bioorthogonal cleavage. Notably, the molecule is structurally designed to facilitate CRET-based red-shifting of the CL signal. This innovative dual-modal imaging strategy is applied to detect endogenous CO, successfully activating the NIR CL signal with the assistance of F^−^*in vitro* and *in vivo*. This technique establishes a synergistic and complementary imaging paradigm, using NIR FL signals for primary screening and NIR CL signals for precise detection.

Despite the promising results, we acknowledge certain limitations in the current study. The probe activation relies on the exogenous addition of multiple components, including F^−^ and Pd-based catalysts. This requirement may pose challenges for *in vivo* applications due to potential biological incompatibilities, such as off-target effects of the additives and their disparate tissue distribution. Future work will focus on developing more biocompatible activation strategies. This includes exploring the use of endogenous enzymes or stimuli to trigger the probes, as well as engineering advanced bioorthogonal uncaging systems that can enhance the precision of the detection.

## Conflicts of interest

There are no conflicts to declare.

## Ethical statement

All animal procedures were performed in accordance with the Guidelines for Care and Use of Laboratory Animals of China Pharmaceutical University and approved by the Animal Ethics Committee of China Pharmaceutical University. All experiments were performed in compliance with the National Standard of the People's Republic of China, GB/T 35892-2018 (Laboratory animals—Guideline for ethical review of animal welfare).

## Supplementary Material

SC-OLF-D5SC09065B-s001

## Data Availability

The data supporting this article have been included as part of the supplementary information (SI). Supplementary information: synthesis procedures, NMR and HRMS spectra, photophysical characterization (UV-vis and fluorescence spectra), and biological imaging details. See DOI: https://doi.org/10.1039/d5sc09065b.
