# Supplementary material for: Dual-locked strategy for fluorescence/chemiluminescence dual-modal imaging reprogramming
Source: Chem Sci. 2026 Mar 6;17(18):9088–97. doi: 10.1039/d5sc09065b (PMC12991303; doi:10.1039/d5sc09065b)
Supplement: SC-017-D5SC09065B-s001 [file SC-017-D5SC09065B-s001.pdf]

Supplementary Information (SI) for Chemical Science.
This journal is © The Royal Society of Chemistry 2026

Supplementary information

Dual-locked Strategy for Fluorescence/Chemiluminescence Dual-modal Imaging Reprogramming

Qi Wang[a], Zhao-Yao Song[a], Zhe Song[b], Guangji Wang[a,*], Le Zhen[a,*]

a. Key Laboratory of Drug Metabolism and Pharmacokinetics, China Pharmaceutical University, Nanjing 210009, Jiangsu, China

b. Instrumental Analysis Center, China Pharmaceutical University, 24 Tong Jia Xiang, Nanjing, Jiangsu 210009 China

Table of contents

1. General Experimental Methods and Materials······S2

2. Compound Synthesis ······S5

3. Summary of activatable small molecule probes based on CRET ······S15

4. Supplemental Figures ······S18

5. High Resolution Mass Spectrometry and NMR Spectra ······S32

References ······S52

1. General Experimental Methods and Materials

1.1 Instruments

^{1}H-NMR and ^{13}C-NMR spectra were recorded on the AVANCE 500MHz or AVANCE NEO 600MHz spectrometer. The mass spectrometry was captured on the Agilent 6230 TOF LC/MS system (U.S.A.). The fluorescence images of cell and tissue sections were captured using a confocal laser scanning microscope (Olympus, Japan) and an objective lens (×20 or ×40). High pressure sterilization pot (Tommy, Japan). High-performance liquid chromatography (HPLC) analysis using the LC-20A system (Shimadzu, Japan), equipped with an LC-20AT pump and SPD-20A detector, and Waters Xselect HSS T3 column as the chromatographic column. The instrument required for in vitro and in vivo imaging of the probe is the IVIS dynamic imaging system (Caliper Life Sciences, Hopkinton, Massachusetts, U.S.A.). Chemiluminescence imaging and intensity quantification were performed using the live imaging software Living Image (Caliper Life Sciences, Hopkinton, Massachusetts, U.S.A.). The UV-Vis absorption spectrum, fluorescence spectrum, chemiluminescence spectrum, and kinetic curve were recorded on the Spectramax iD5 multimode microplate reader (Molecular Devices) and microplate reader (BIO-TEK Synergy HTX).

1.2 Materials

Unless special stated, all solvents and chemicals were purchased from commercial suppliers in analytical grade and used without further purification. CORM-3, $PdCl_2$ and NaF were purchased from Adamas-bata. Ferroheme (heme) was purchased from Bidepharm. Lipopolysaccharides (LPS) was purchased from Adamas-life. HepG2 and HeLa cells lines were purchased from Pricella Biotechnology Co., Ltd. (Wuhan, China). Fetal bovine serum (FBS), penicillin-streptomycin (PS), dulbecco's modified eagle

medium (DMEM) and minimum essential medium (MEM) were purchased from Gibco (Waltham, MA, USA). The stock solutions (1.0 mM) of control probe (**C-01, F-01**) and **FTC-01** were prepared by dissolving in DMSO. Water was purified and doubly distilled by a Milli-Q system (Millipore, USA).

1.3 Preparation of reactive oxygen species

The hydroxyl radical (•OH) was generated through fenton reaction between H_2O_2 and $FeSO_4 \cdot 7H_2O$. Dissolve potassium superoxide (KO_2) in anhydrous DMSO solution and stir vigorously to obtain O_2^- solution. Single oxygen ($^1O_2$) was obtained by adding NaClO (100 mM in H_2O) to a solution containing large excess of H_2O_2. All these reagents were prepared before the experiment and were not stored.

1.4 Cell culture

HeLa cells were cultured in MEM with 10% FBS and 1% penicillin-streptomycin. HepG2 cells were cultured in DMEM with 10% FBS and 1% penicillin-streptomycin. The cells were placed in 37 ℃, 5% CO_2 incubator and passaged once every other day. Cells from 5th to 20th passages were taken for the experiment.

1.5 Cytotoxicity study

A standard CCK8 assay was used to evaluate the cytotoxicity of **FTC-01**, $PdCl_2$, CORM-3 and NaF. Cells were seeded at 5000 cells per well in 96-well plates in 200 μL of medium with 10% FBS and incubated at 37 ℃ in 5% CO2 for 24 h. After that, cells were treated with different concentrations of **FTC-01**, $PdCl_2$, CORM-3 and NaF for 8 h. After removing the above medium and washed with PBS for three times, the cells were incubated with medium containing CCK8 reagent (10%, 100 μL). After the plates were shaken for 2 h at 37 ℃, the absorbance was then measured at 450 nm by a multi-detection microplate reader. The cytotoxic effects (VR) were assessed by the following equation: $VR = A/A_0 \times 100\%$, where A and A_0 represent absorbance of the experimental

group and control group respectively. The assays were performed in six sets for each concentration.

1.6 Cellular imaging

Fluorescent images of HeLa and HepG2 Cells were acquired on Zeiss LSM700 confocal laser-scanning microscope with an objective lens (×40). The fluorescence was acquired from 680 to 720 nm upon excitation at 555 nm. Scale bar: 50 μm. Cells were plated on Petridishes (Φ = 20 mm) and allowed to adhere for 24 h before imaging.

The FTC-01 probe treatment solution for cellular imaging was prepared via a two-step dilution in serum-free DMEM to achieve a final concentration of 10 μM probe and 2% DMSO. First, an intermediate working solution (200 μM) was freshly prepared by mixing 20 μL of the 1 mM stock solution (in DMSO) with 80 μL of serum-free DMEM, followed by brief vortexing and 1-2 minutes of ultrasonication to form a homogeneous intermediate dispersion (200 μM probe in 20% DMSO). For the final treatment, 10 μL of this intermediate solution was diluted with 190 μL of serum-free DMEM, yielding 200 μL of the desired 10 μM probe solution in approximately 2% DMSO.

For cell treatment, cells plated in a 20-mm Petri dish were washed, and the 200 μL of the freshly prepared solution was added to cover the monolayer for incubation prior to imaging. This optimized protocol minimizes the local impact of DMSO while ensuring consistent probe delivery.

The chemiluminescence images of HeLa and HepG2 cells were acquired on a small animal in vivo imaging system. The cells were plated on a black 96-well plate with a transparent bottom and adhered for 24 hours before imaging. The chemiluminescence signal was obtained at 540 and 700 nm using a filter in the bioluminescence mode. Additionally, in the experimental model of cellular inflammation induced by LPS and heme, the fluorescence images of HeLa and HepG2

cells were captured and quantified using the small animal in vivo imaging system in the fluorescence mode.

1.7 Animals

All animal protocols were approved by the Institutional Animal Care and Use Committee of China Pharmaceutical University (2024-07-058). Male C57BL/6 mice (6~8 weeks, 23~25 g, SPF grade) were purchased from Beijing Vital River Laboratory Animal Technology Co., Ltd., and adaptively housed in separate cages in the IVC animal room for a week.

2. Compound Synthesis

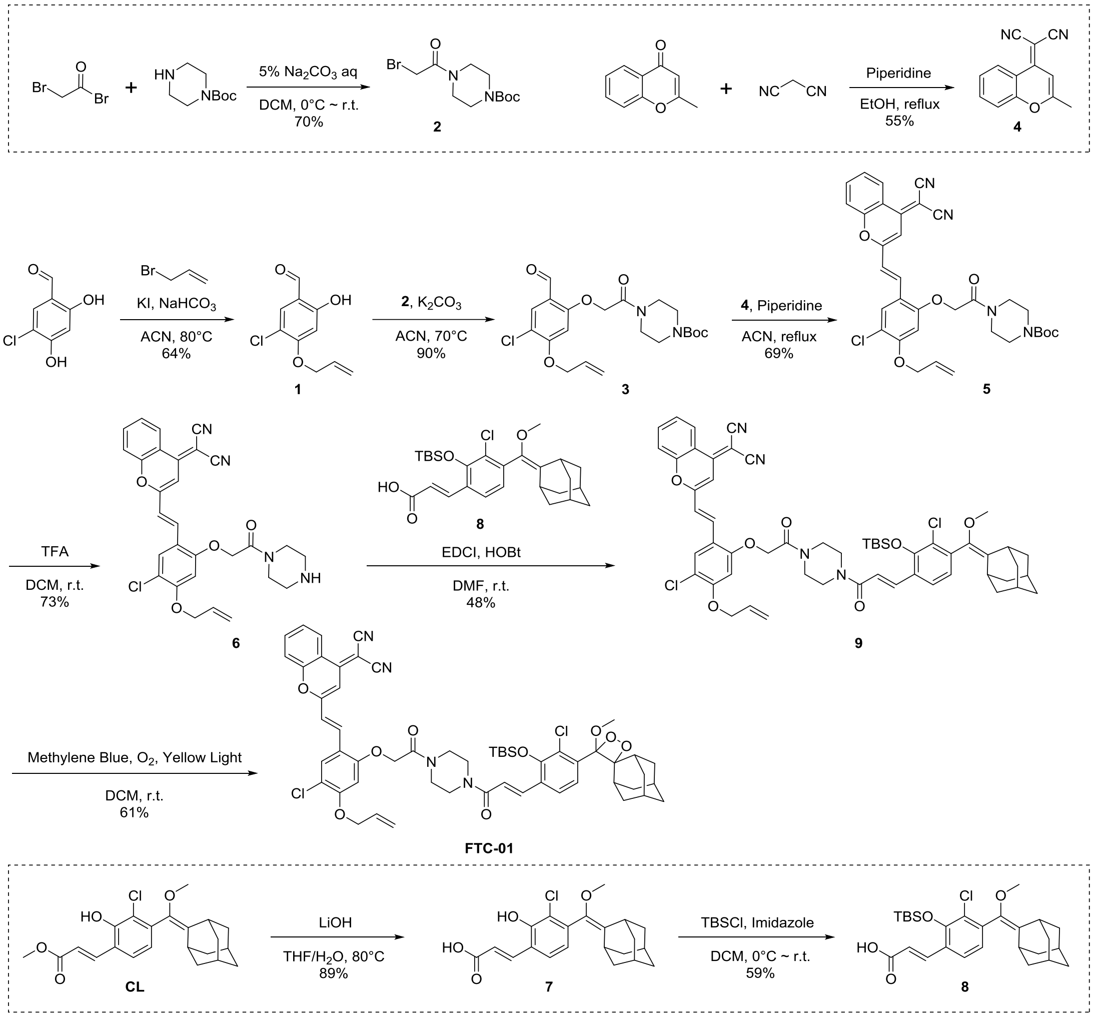

Scheme S1. Synthesis of **FTC-01**.

Synthesis of compound 1. 5-chloro-2,4-dihydroxybenzaldehyde (860 mg, 5 mmol) was dissolved in 50 mL ACN, then KI (664 mg, 4 mmol) and $NaHCO_3$ (630 mg, 7.5 mmol) were added successively. After stirring at room temperature for 10 minutes, 3-bromoprop-1-ene (476 μL, 5.5 mmol) was added. The reaction was heated to 80 ℃ and stirred for 24 h. Upon completion, the reaction was allowed to cool to room temperature, washed with H_2O and sat. NH_4Cl, extracted with EtOAc, dried with Na_2SO_4, then filtered and concentrated under reduced pressure. Column chromatography in 5% EtOAc/Hexane yielded compound **1** (680 mg, 64% yield) as a

white solid. ^{1}H NMR (600 MHz, Chloroform-*d*) δ 11.38 (s, 1H), 9.65 (s, 1H), 7.50 (s, 1H), 6.46 (d, *J* = 1.8 Hz, 1H), 6.12 – 5.96 (m, 1H), 5.49 (dd, *J* = 17.4, 1.9 Hz, 1H), 5.36 (dt, *J* = 10.6, 1.4 Hz, 1H), 4.67 – 4.60 (m, 2H). ^{13}C NMR (151 MHz, Chloroform-*d*) δ 193.58, 162.77, 160.66, 133.86, 131.14, 118.69, 114.86, 114.49, 101.35, 69.85. HRMS(ESI) calcd. for $C_{10}H_9ClO_3$ [M-H$^+$] m/z 211.01675; found 211.01702.

Synthesis of compound 2. Tert-butyl piperazine-1-carboxylate (931 mg, 5 mmol) was dissolved in 15 mL 5% Na_2CO_3 aqueous solution, then equal volume of DCM was added. After stirring at 0 ℃ for 10 minutes, 2-bromoacetyl bromide (467.5 μL, 5.5 mmol) was added. The reaction continued at 0 ℃ for 30 min, then moved to room temperature and reacted overnight. Upon completion, the reaction was washed with 1N HCl and sat. NaCl successively, extracted with DCM, dried with Na_2SO_4, filtered, and concentrated under reduced pressure. Column chromatography in 50% EtOAc/Hexane yielded compound **1** (680 mg, 70% yield) as a white solid. ^{1}H NMR (500 MHz, DMSO-*d*$_6$) δ 3.86 (s, 2H), 3.62 – 3.42 (m, 8H), 1.47 (s, 9H). ^{13}C NMR (600 MHz, Chloroform-*d*) δ 165.13, 154.11, 79.97, 46.24, 41.65, 28.11, 25.75. HRMS (ESI) calcd. for $C_{11}H_{19}BrN_2O_3$ [M+Na$^+$] m/z 329.04713; found 329.04779.

Synthesis of compound 3. Compound **1** (425 mg, 2 mmol) and compound **2** (614 mg, 2 mmol) were dissolved in 20 mL ACN, then K_2CO_3 (414 mg, 3 mmol) was added. The reaction was heated to 70° C and stirred for 8 h. Upon completion, the reaction was washed with 1N HCl and sat. NaCl successively, extracted with EtOAc, dried with Na_2SO_4, filtered, and concentrated under reduced pressure. Column chromatography in 50% EtOAc/Hexane yielded compound **3** (788 mg, 90% yield) as a colorless oil. ^{1}H NMR (500 MHz, Chloroform-*d*) δ 10.23 (s, 1H), 7.79 (s, 1H), 6.63 (s, 1H), 6.03 (ddt, *J* = 16.0, 10.3, 5.1 Hz, 1H), 5.50 (d, *J* = 17.5 Hz, 1H), 5.38 – 5.30 (m, 1H), 4.83 (s, 2H), 4.66 (d, *J* = 5.1 Hz, 2H), 3.57 (t, *J* = 5.1 Hz, 4H), 3.40 (dt, *J* = 10.9, 4.8 Hz, 4H), 1.45 (s, 9H). ^{13}C NMR (151 MHz, Chloroform-*d*) δ 186.28, 165.57, 160.02, 159.72, 154.17,

131.31, 130.09, 118.71, 116.66, 98.50, 80.41, 69.90, 68.41, 45.21, 41.94, 28.19. HRMS(ESI) calcd. for $C_{21}H_{27}ClN_2O_6$ [M+Na$^+$] m/z 461.14499; found 461.14403.

Synthesis of compound 4. 2-methyl-4H-chromen-4-one (800 mg, 5 mmol) and malononitrile (660 mg, 10 mmol) were dissolved in 50 mL EtOH, then piperidine (420 μL, 4 mmol) was added slowly. The reaction was heated to 85° C and stirred for 4 h under Ar atmosphere. Upon completion, the reaction was washed with 1N HCl and sat. NaCl successively, extracted with EtOAc, dried with Na_2SO_4, filtered, and concentrated under reduced pressure. Column chromatography in 2% MeOH/DCM yielded compound **4** (572 mg, 55% yield) as a pink soild. ^{1}H NMR (600 MHz, DMSO-d_6) δ 8.73 (dd, J = 8.4, 1.4 Hz, 1H), 7.91 (ddd, J = 8.6, 7.2, 1.4 Hz, 1H), 7.71 (dd, J = 8.5, 1.3 Hz, 1H), 7.63 (ddd, J = 8.4, 7.2, 1.2 Hz, 1H), 6.80 (s, 1H), 2.52 (s, 3H).

Synthesis of compound 6. Compound **3** (634 mg, 1.44 mmol) and Compound **4** (329 mg, 1.58 mmol) were dissolved in 20 mL ACN, then piperidine (110 μL, 0.72 mmol) was added slowly. The reaction was heated to 85 ℃ and stirred for 6 h under Ar atmosphere. Upon completion, the reaction was washed with 1N HCl and sat. NaCl successively, extracted with EtOAc, dried with Na_2SO_4, filtered, and concentrated under reduced pressure. Column chromatography in 2% MeOH/DCM yielded compound **5** (624 mg, 69% yield) as a brown solid.

Compound **5** (624 mg, 0.99 mmol) was dissolved in 10 mL DCM containing 50% trifluoroacetic acid. The reaction was stirred overnight at 0 ℃. Upon completion, adjust the reaction solution to pH = 7 with saturated sodium bicarbonate solution, then wash the reaction solution with sat. NaCl, extract with EtOAc, dry with Na_2SO_4, filter, and concentrate under reduced pressure. Column chromatography in 5% MeOH/DCM yielded compound **6** (382 mg, 73% yield) as a red-brown solid. ^{1}H NMR (500 MHz, DMSO-d_6) δ 8.64 (d, J = 8.3 Hz, 1H), 7.84 (t, J = 7.9 Hz, 1H), 7.78 – 7.68 (m, 2H), 7.61 (dd, J = 8.4, 2.8 Hz, 1H), 7.53 (t, J = 7.8 Hz, 1H), 7.46 (dd, J = 16.1, 3.0 Hz, 1H), 6.81 (d, J = 2.9 Hz, 1H), 6.73 (s, 1H), 6.06 (ddt, J = 16.1, 10.5, 5.3 Hz, 1H), 5.48 (d, J

= 17.3 Hz, 1H), 5.33 (d, *J* = 10.5 Hz, 1H), 4.97 (s, 2H), 4.69 (d, *J* = 5.3 Hz, 2H), 3.48 – 3.38 (m, 4H), 2.73 (dt, *J* = 31.9, 5.0 Hz, 4H). ^{13}C NMR (151 MHz, Chloroform-*d*) δ 165.94, 162.66, 157.88, 156.12, 152.68, 152.19, 150.46, 138.71, 136.12, 134.51, 132.70, 132.28, 131.78, 127.60, 126.82, 126.78, 125.83, 125.66, 123.49, 118.47, 116.70, 111.25, 99.10, 69.95, 57.17, 36.92, 36.47, 32.80, 29.61. HRMS(ESI) calcd. for $C_{29}H_{25}ClN_4O_4$ [M+H$^+$] m/z 529.16371; found 529.16124.

Synthesis of compound 7. Compound **CL** was synthesized according to the published literature [1]. Compound **CL** (1100 mg, 2.83 mmol) was dissolved in 10 mL THF. Then LiOH aqueous solution (10 mL, 1M) was added dropwise. The reaction was heated to 85 ℃ and stirred for 6 h under Ar atmosphere. Upon completion, adjust the reaction solution to pH = 4~5 with 1N HCl, then wash the reaction solution with sat. NaCl, extract with EtOAc, dry with Na_2SO_4, filter, and concentrate under reduced pressure. Column chromatography in 50% EtOAc/Hexane yielded compound **7** (942 mg, 89% yield) as a white solid. ^{1}H NMR (600 MHz, Methanol-*d*$_4$) δ 8.02 (d, *J* = 16.1 Hz, 1H), 7.51 (d, *J* = 7.9 Hz, 1H), 6.85 (d, *J* = 7.9 Hz, 1H), 6.62 (d, *J* = 16.1 Hz, 1H), 3.31 (d, *J* = 1.2 Hz, 3H), 3.25 (d, *J* = 3.9 Hz, 1H), 2.10 (t, *J* = 3.5 Hz, 1H), 1.97 (d, *J* = 14.0 Hz, 4H), 1.82 (dq, *J* = 57.8, 14.6 Hz, 8H). ^{13}C NMR (600 MHz, Methanol-*d*$_4$) δ 169.19, 151.99, 139.93, 139.47, 136.54, 131.07, 125.75, 122.99, 122.75, 121.86, 119.09, 55.78, 38.63, 38.53, 38.23, 38.08, 36.66, 32.83, 29.52, 28.33, 28.21. HRMS(ESI) calcd. for $C_{21}H_{23}ClO_4$ [M+H$^+$] m/z 375.13576; found 375.13340.

Synthesis of compound 8. Compound **7** (280 mg, 0.75 mmol) was dissolved in 8 mL DCM. Then imidazole (105 mg, 1.55 mmol) and TBSCl (120 mg, 0.79 mmol) were added successively in an ice water bath. The reaction was stirred for 2.5 h under Ar atmosphere at room temperature. Upon completion, the reaction was washed with H_2O and sat. NaCl successively, extracted with DCM, dried with Na_2SO_4, filtered, and concentrated under reduced pressure. Column chromatography in 50% EtOAc/Hexane yielded compound **8** (207 mg, 59% yield) as a white solid. ^{1}H NMR (500 MHz, DMSO)

δ 7.86 (d, *J* = 16.1 Hz, 1H), 7.61 (d, *J* = 8.0 Hz, 1H), 6.80 (d, *J* = 7.9 Hz, 1H), 6.54 (d, *J* = 16.1 Hz, 1H), 3.20 (s, 3H), 3.17 (s, 1H), 2.01 – 1.96 (m, 1H), 1.93 – 1.84 (m, 5H), 1.79 – 1.67 (m, 8H), 0.84 (s, 10H), -0.05 (s, 6H). ^{13}C NMR (126 MHz, $CDCl_3$) δ 167.63 , 151.69 , 139.85 , 138.34 , 136.34 , 129.53 , 125.92 , 123.53 , 122.86 , 122.45 , 120.06 , 56.32 , 38.59 – 37.70 (m), 32.36 , 28.95 , 27.63 (d, *J* = 16.2 Hz), 25.76 , 17.74 , -3.25 . HRMS (ESI) for $C_{27}H_{36}ClO_4Si$ $[M -H]^-$ m/z 487.20769, found 487.20755.

Synthesis of FTC-01. Compound **8** obtained in the previous step was dissolved in 10 mL DMF. Then EDCI (166 mg, 0.87 mmol), HOBt (97 mg, 0.72 mmol) were added in sequence to stir for 30 min. Compound **6** (382 mg, 0.72 mmol) was added. The reaction was stirred for 12 h at room temperature. Upon completion, the reaction was washed with H_2O and sat. NaCl successively, extracted with EtOAc, dried with Na_2SO_4, filtered, and concentrated under reduced pressure. Column chromatography in 2% MeOH/DCM yielded compound **9** (350 mg, 48% yield) as a red-brown solid. ^{1}H NMR (500 MHz, $CDCl_3$) δ 8.89 (dd, *J* = 8.4, 1.3 Hz, 1H), 7.98 (d, *J* = 15.5 Hz, 1H), 7.75 – 7.68 (m, 2H), 7.57 (s, 1H), 7.52 (d, *J* = 8.3 Hz, 1H), 7.47 – 7.38 (m, 2H), 6.92 (dd, *J* = 14.1, 7.3 Hz, 2H), 6.84 (s, 1H), 6.78 (dd, *J* = 15.4, 5.5 Hz, 1H), 6.67 (s, 1H), 6.07 (ddt, *J* = 15.6, 10.1, 4.8 Hz, 1H), 5.52 (d, *J* = 17.2 Hz, 1H), 5.36 (d, *J* = 10.5 Hz, 1H), 4.90 (s, 2H), 4.67 (d, *J* = 5.0 Hz, 2H), 3.70 (d, *J* = 15.5 Hz, 10H), 3.31 (s, 2H), 3.27 (d, *J* = 14.7 Hz, 1H), 1.97 – 1.76 (m, 7H), 1.75 – 1.52 (m, 6H), 1.04 (d, *J* = 1.5 Hz, 9H), 0.22 (d, *J* = 1.9 Hz, 6H). HRMS(ESI) calcd. for $C_{56}H_{60}Cl_2N_4O_9Si$ $[M+H^+]$ m/z 999.3681; found 999.3673.

Compound **9** (350 mg, 0.35 mmol) was dissolved in 15 mL DCM. A catalytic amount of methylene blue (4 mg) was added and oxygen was bubbled through the solution, while irradiating with yellow light. Upon completion, the reaction was washed with H_2O and sat. NaCl successively, extracted with DCM, dried with Na_2SO_4, filtered, and concentrated under reduced pressure. Column chromatography in 2% MeOH/DCM yielded compound **FTC-01** (220 mg, 61% yield) as a reddish brown solid. ^{1}H NMR

(500 MHz, Chloroform-*d*) δ 8.78 (d, *J* = 8.3 Hz, 1H), 7.94 (dd, *J* = 15.5, 11.0 Hz, 1H), 7.75 – 7.62 (m, 3H), 7.56 – 7.45 (m, 3H), 7.41 – 7.33 (m, 1H), 6.84 (dd, *J* = 15.7, 7.5 Hz, 2H), 6.75 (s, 1H), 6.60 (s, 1H), 6.05 (ddd, *J* = 12.2, 10.6, 5.3 Hz, 1H), 5.50 (dt, *J* = 17.2, 1.6 Hz, 1H), 5.38 – 5.31 (m, 1H), 4.91 (s, 2H), 4.64 (d, *J* = 5.1 Hz, 2H), 3.70 (d, *J* = 23.8 Hz, 8H), 3.18 (s, 3H), 2.98 (s, 1H), 2.00 – 1.29 (m, 13H), 1.03 (d, *J* = 4.0 Hz, 9H), 0.20 – 0.16 (m, 6H). ^{13}C NMR (600 MHz, Chloroform-*d*) δ 165.98, 165.45, 165.19, 158.00, 156.57, 156.25, 152.76, 152.30, 151.61, 150.97, 139.78, 139.59, 139.16, 134.68, 132.32, 131.92, 131.39, 130.10, 129.57, 127.81, 125.99, 125.91, 125.78, 124.88, 124.32, 118.66, 118.59, 117.84, 117.73, 116.89, 116.53, 115.92, 99.16, 96.45, 70.08, 62.24, 57.00, 49.62, 45.41, 42.36, 39.22, 38.99, 38.63, 38.54, 37.15, 36.64, 33.92, 33.57, 33.02, 32.69, 32.32, 31.64, 31.53, 29.67, 28.43, 28.29, 26.25, 26.03, 25.99, 25.97, 25.86, 25.70, 18.81, -3.14, -3.16, -3.26, -3.33, -3.53. HRMS(ESI) calcd. for $C_{56}H_{60}Cl_2N_4O_9Si$ [M+H$^+$] m/z 1031.35794; found 1031.35876.

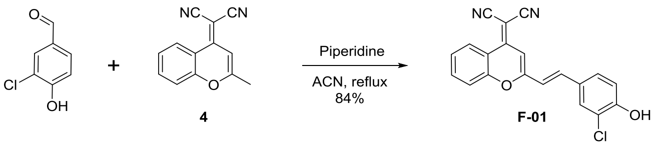

Scheme S2. Synthesis of **F-01**.

Synthesis of F-01. 3-chloro-4-hydroxybenzaldehyde (157 mg, 1 mmol) and compound **4** (208 mg, 1 mmol) was dissolved in 15 mL ACN. Then piperidine (77 μL, 0.5 mmol) was added slowly. The reaction was heated to 85 ℃ and stirred for 6 h under Ar atmosphere. Upon completion, the reaction was washed with 1N HCl and sat. NaCl successively, extracted with EtOAc, dried with Na_2SO_4, filtered, and concentrated under reduced pressure. Column chromatography in 5% MeOH/DCM yielded compound **F-01** (290 mg, 84% yield) as a brown solid. ^{1}H NMR (500 MHz, DMSO-*d*$_6$) δ 10.81 (s, 1H), 8.67 (d, *J* = 8.4 Hz, 1H), 7.87 (t, *J* = 7.8 Hz, 1H), 7.80 (s, 1H), 7.70 (d, *J* = 8.5 Hz, 1H), 7.63 – 7.47 (m, 3H), 7.31 (d, *J* = 16.0 Hz, 1H), 6.99 (d, *J* = 8.4 Hz, 1H), 6.90 (s, 1H). ^{13}C NMR (151 MHz, DMSO-*d*$_6$) δ 158.74, 155.57, 153.18, 152.34,

137.95, 135.70, 129.85, 129.24, 127.77, 126.45, 124.99, 121.05, 119.32, 117.91, 117.58, 117.45, 117.31, 116.24, 106.58, 60.12. HRMS(ESI) calcd. for $C_{20}H_{11}ClN_2O_2$ [M-H$^+$] m/z 345.04363; found 345.04361.

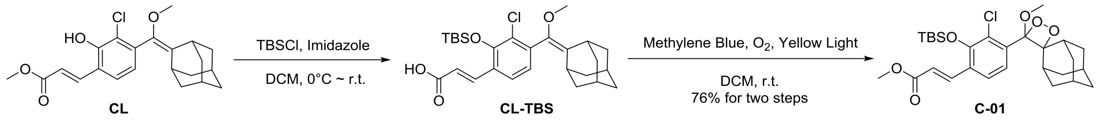

Scheme S3. Synthesis of **C-01**.

Synthesis of C-01. Compound **CL** (194 mg, 0.5 mmol) was dissolved in 3 mL DCM. Imidazole (68 mg, 1 mmol) and TBS-Cl (91 mg, 0.6 mmol) were added successively to it. The reaction was stirred for 30 min under Ar atmosphere. Upon completion, the reaction was washed with sat. NaCl, extracted with DCM, dried with Na_2SO_4, filtered, and concentrated under reduced pressure. Column chromatography in 5% EtOAc/Hexane yielded compound **CL-TBS** as a colorless oil.

Compound **CL-TBS** from the previous step was dissolved in 3 mL DCM. A catalytic amount of methylene blue (2 mg) was added and oxygen was bubbled through the solution, while irradiating with yellow light. Upon completion, the reaction was washed with H_2O and sat. NaCl successively, extracted with DCM, dried with Na_2SO_4, filtered, and concentrated under reduced pressure. Column chromatography in 5% EtOAc/Hexane yielded compound **C-01** (203 mg, 76% yield for two steps) as a colorless oil. ^{1}H NMR (500 MHz, Chloroform-*d*) δ 8.02 (d, *J* = 16.2 Hz, 1H), 7.78 (d, *J* = 8.4 Hz, 1H), 7.59 (d, *J* = 8.4 Hz, 1H), 6.46 (d, *J* = 16.1 Hz, 1H), 3.85 (s, 3H), 3.24 (s, 3H), 3.04 (s, 1H), 2.15 – 1.58 (m, 13H), 1.10 (s, 9H), 0.23 (d, *J* = 7.7 Hz, 6H). ^{13}C NMR (600 MHz, Chloroform-*d*) δ 166.97, 151.65, 139.84, 135.02, 129.00, 126.06, 125.14, 124.48, 119.38, 111.97, 96.41, 51.79, 49.61, 47.02, 39.30, 36.66, 33.94, 33.54, 32.68, 32.29, 31.64, 31.54, 27.51, 26.26, 26.00, 25.88, 18.83, -3.28.

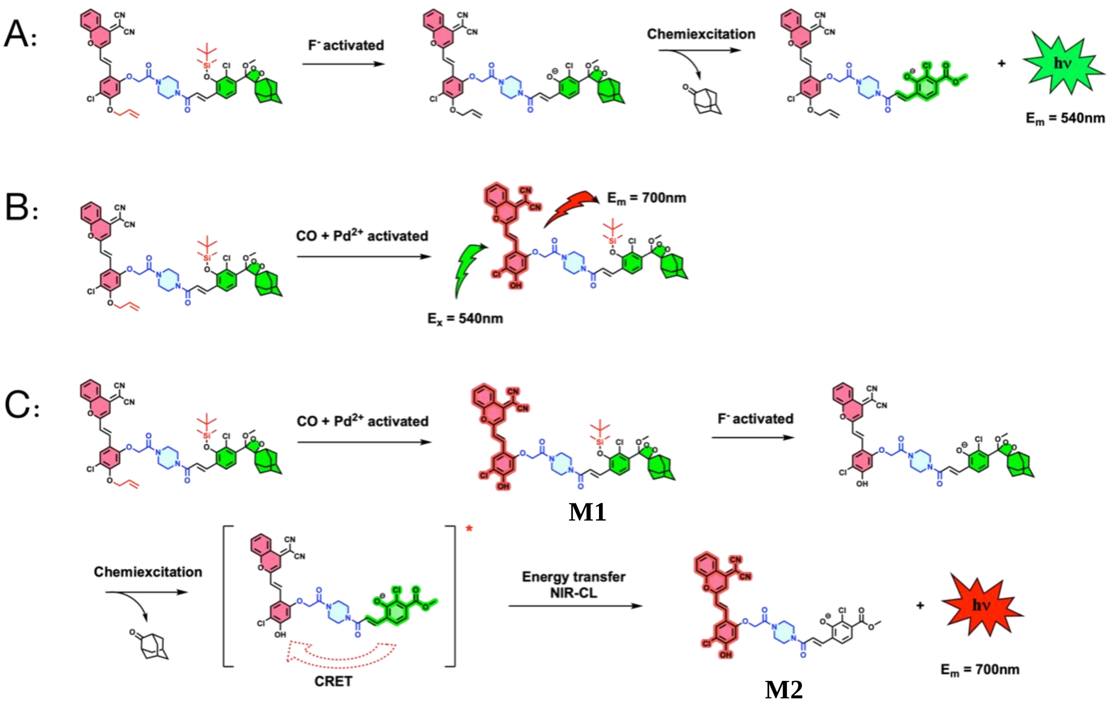

Scheme S4. (A) The chemiluminescence mechanism of **FTC-01** in response to F⁻. (B) The fluorescence mechanism of **FTC-01** in response to CO. (C) The activation mechanism of **FTC-01** triggered by F⁻ and CO. **M1** and **M2** are proposed intermediates during the probe activation.

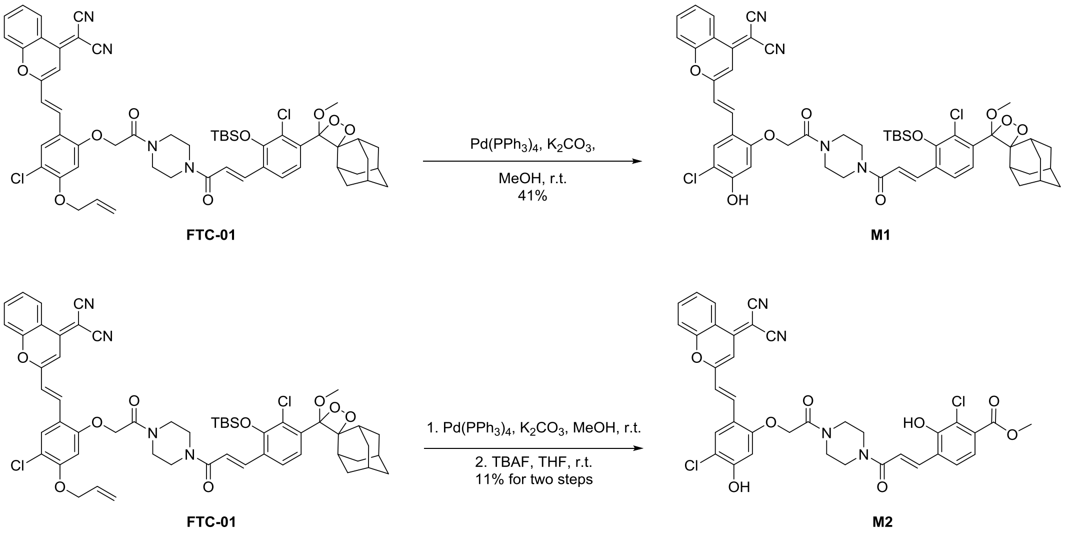

Scheme S5. Synthesis of **M1** and **M2**.

Under an argon atmosphere, the probe **FTC-01** (100 mg, 0.1 mmol) and $Pd(PPh_3)_4$ (5 mg, 0.05 eq) were dissolved in MeOH/THF (v:v = 1:2, 4 + 8 mL) and stirred at room temperature for 5 minutes. Anhydrous K_2CO_3 (42 mg, 0.3 mmol) was then added, and the mixture was stirred at room temperature for 2 hours. The mixture was then extracted with ethyl acetate and purified by column chromatography to yield **M1** (40 mg, 41% yield) as a dark-red solid. ^{1}H NMR (500 MHz, DMSO) δ 8.72 (s, 1H), 7.92 – 7.80 (m, 5H), 7.71 (d, *J* = 8.5 Hz, 1H), 7.65 – 7.42 (m, 5H), 6.94 (s, 1H), 6.58 (s, 1H), 5.05 (s, 2H), 3.84 – 3.50 (m, 9H), 3.12 (s, 3H), 2.88 (s, 1H), 2.06 – 1.92 (m, 2H), 1.82 – 1.43 (m, 9H), 0.84 (s, 9H), -0.04 (s, 6H). HRMS(ESI) calcd. for $C_{53}H_{55}O_9N_4SiCl_2$ $[M-H]^-$ m/z 989.31208; found 989.31213.

Under an argon atmosphere, the probe **FTC-01** (100 mg, 0.1 mmol) and $Pd(PPh_3)_4$ (5 mg, 0.05 eq) were dissolved in MeOH/THF (v:v = 1:2, 4 + 8 mL) and stirred at room temperature for 5 minutes. Anhydrous K_2CO_3 (42 mg, 0.3 mmol) was then added, and the mixture was stirred at room temperature for 2 hours. After the reaction solution had darkened in color, a solution of tetrabutylammonium fluoride (1.0 M in THF, 0.2 mL) was added, and the reaction was continued with stirring at room temperature for an additional 0.5 hour. The mixture was then extracted with ethyl acetate and purified by column chromatography to yield **M2** (7.9 mg, 11% yield for two steps) as a brown solid. ^{1}H NMR (500 MHz, DMSO) δ 8.71 (s, 1H), 7.94 – 7.74 (m, 5H), 7.76 – 7.68 (m, 1H), 7.63 – 7.42 (m, 2H), 7.42 – 7.22 (m, 2H), 6.93 (s, 1H), 6.58 (s, 1H), 5.05 (s, 2H), 3.86 (s, 3H), 3.82 – 3.50 (m, 8H). HRMS(ESI) calcd. for $C_{37}H_{29}O_8N_4Cl_2$ $[M+H]^+$ m/z 727.13570; found 727.13605.

3. Summary of activatable small molecule probes based on CRET

Table S1. Summary of activatable small molecule probes based on the **CRET** process for *in vivo* bioimaging applications.

	Structures	Analyte	Donor λ_{ex}/[nm]<br><br>Acceptor λ_{em}/[nm]	CRET Efficiency	Applications	Ref
1	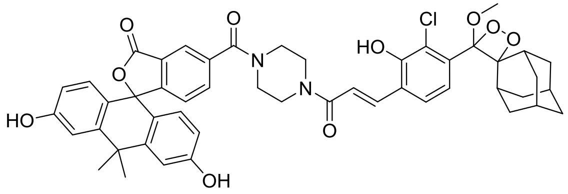	H^+/OH^-	D: 530 nm<br><br>A: 580 nm	---	Ratiometric pH imaging in live animals	[2]
2	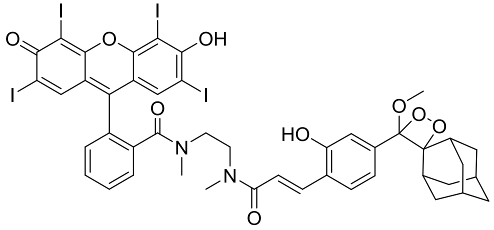	---	D: 550 nm<br><br>A: 550 nm	---	Photodynamic therapy (PDT) via $^1O_2$ generated by CRET	[3]

3	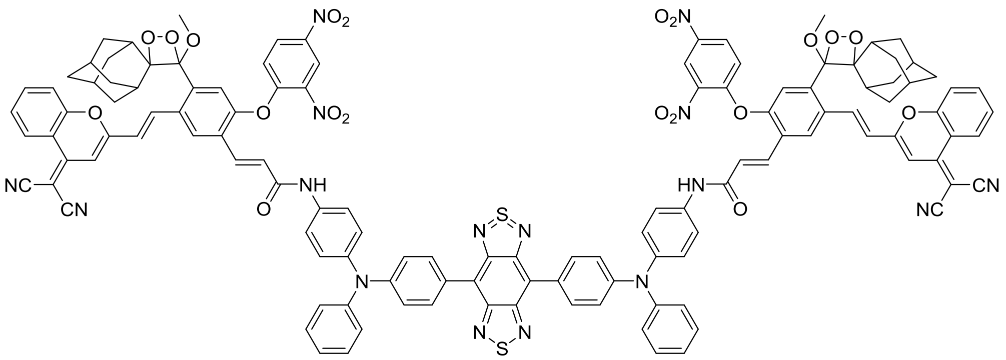	H_2S	D: 530 nm<br>A: 950 nm	95%	H_2S imaging in vivo	[4]
4	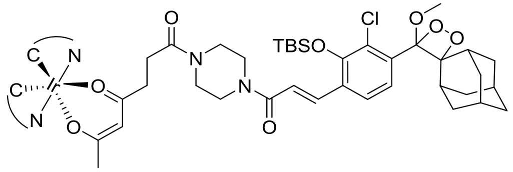	Oxygen	D: 530 nm<br>A: 615/668/705 nm	---	Ratiometric imaging of oxygen	[5]
5	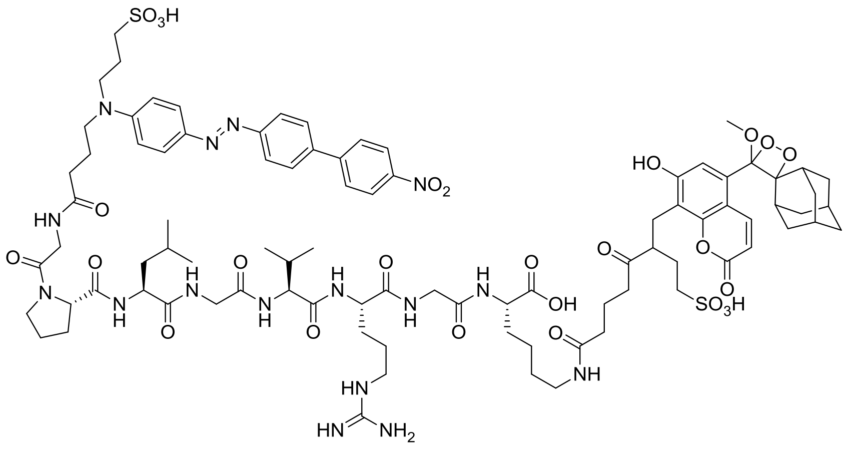	endo-protease	D: 460 nm	---	Exploiting energy transfer to detect endo-protease activity.	[6]

6	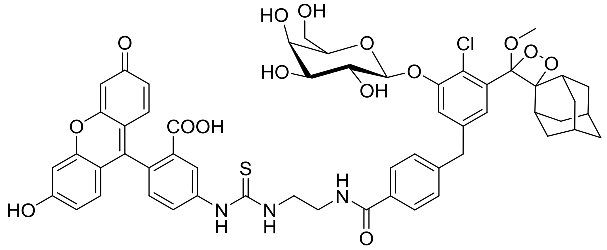	β-galactosidase	D: 470 nm<br>A: 534 nm	---	β-galactosidase imaging in vivo	[7]
	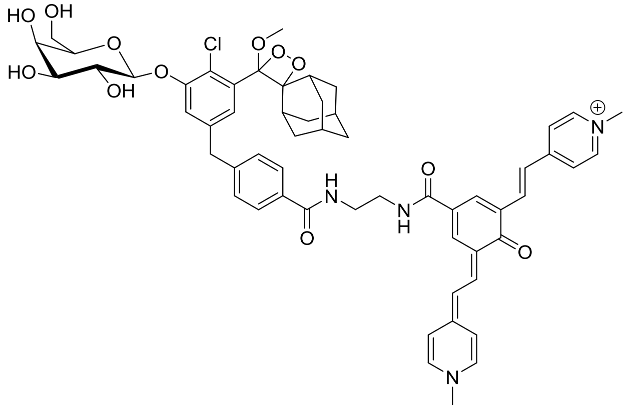		D: 470 nm<br>A: 714 nm	---		
7	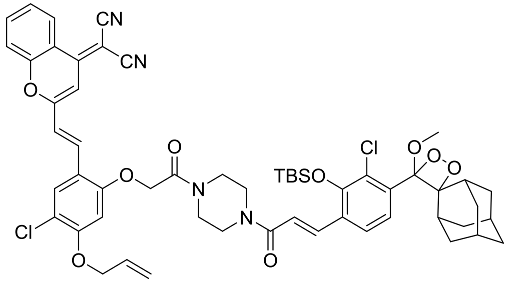<br>**FTC-01**	F^- and CO	D: 540 nm<br>A: 700 nm	93%	NIR FL/ NIR CL dual-channel imaging of CO in cells and living animals	This work

--- : Not mentioned

4. Supplemental Figures

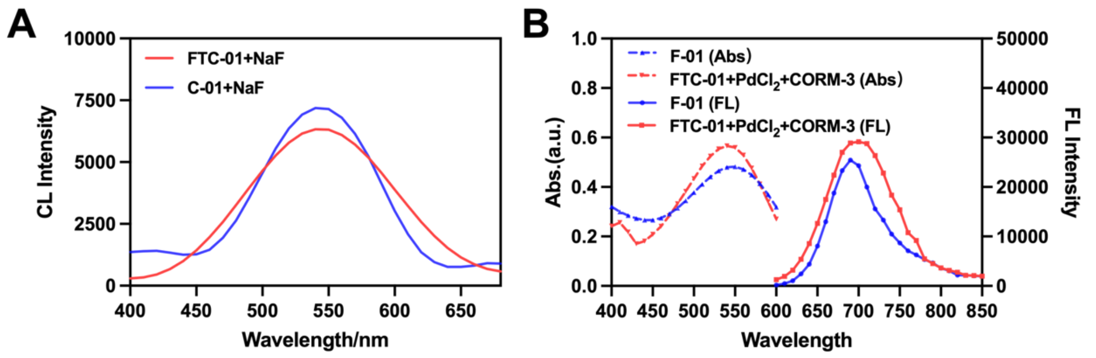

Figure S1. (A) CL spectra of **C-01** (10 μM) + NaF (20 μM) and **FTC-01** (10 μM) + NaF (20 μM). (B) Absorption spectra and FL spectra of **F-01** (10 μM) and **FTC-01** (10 μM) + $PdCl_2$ (10 μM) + CORM-3 (50 μM). λ_{ex} = 540 nm. All the measurements were performed in PBS (10 mM, pH=7.4, 50% DMSO) at 37 ℃.

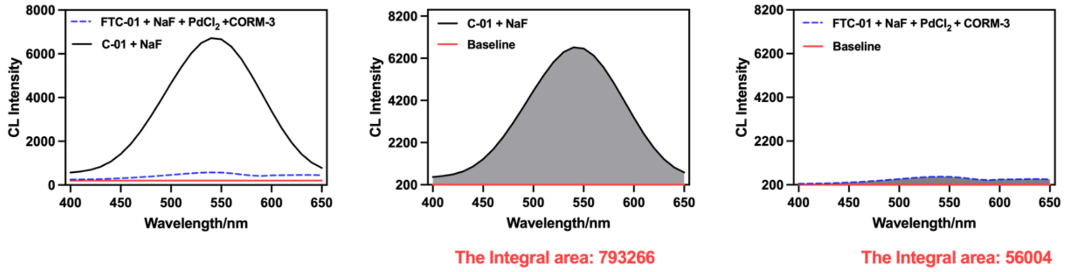

Figure S2. CRET Efficiency Calculation. The chemiluminescence resonance energy transfer efficiency (CRET) from donor to acceptor could be estimated using the following equation [2]:

$$\boldsymbol{ET = 1 - \frac{\int_x^y C_{CF-00}(\lambda)d\lambda}{\int_x^y C_{c-01}(\lambda)d\lambda}}$$

$\int_x^y C_{CF-00}(\lambda)d\lambda$ represents the integrated area of the CL emission spectrum of **FTC-01** after pre-incubation with $PdCl_2$ and CORM-3 for 60 min and subsequent addition of NaF, while $\int_x^y C_{c-01}(\lambda)d\lambda$ denotes the integrated area of the CL emission spectrum of **C-01** in the presence of NaF. Based on the calculation results, the CRET efficiency of **FTC-01** is approximately 93.0%.

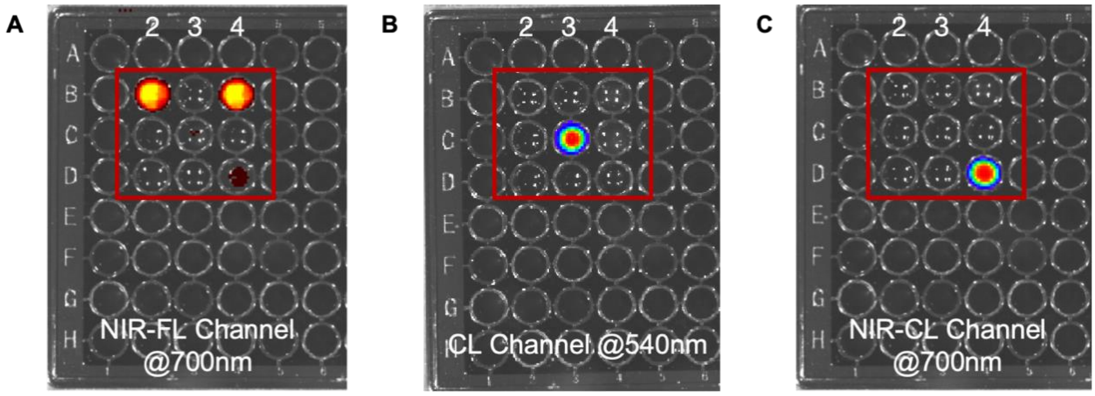

Figure S3. Signal response diagrams of **FTC-01** (10 μM) under different channels. Column 2: $PdCl_2$ (10 μM) + CORM-3 (50 μM); Column 3: NaF (50 μM); Column 4: $PdCl_2$ (10 μM) + CORM-3 (50 μM) pretreated for 1h then NaF (50 μM) was added. λ_{ex} = 540 nm. All the measurements were performed in PBS (10 mM, pH=7.4, 50% DMSO) at 37 ℃. Data as mean values ± SD (n = 3).

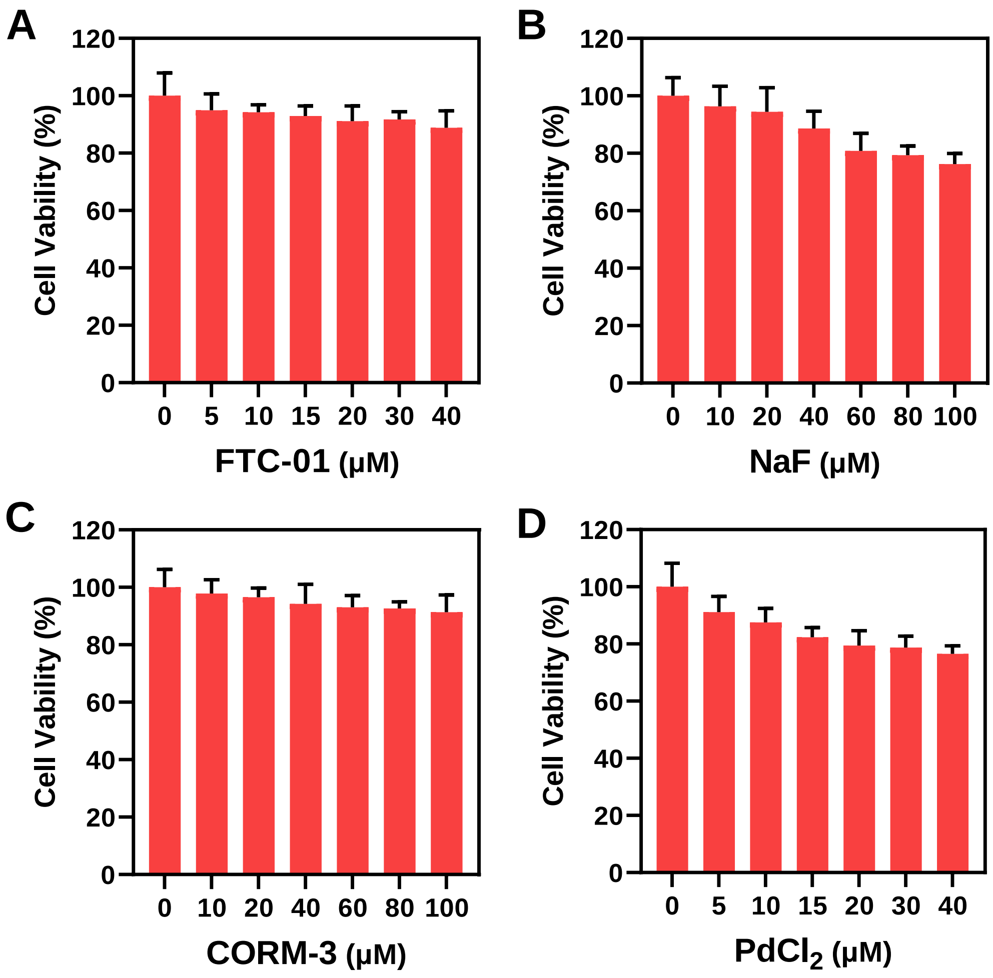

Figure S4. Effects of compounds on cell viability (CCK-8 method). HepG2 cells were treated with **FTC-01** (A), NaF (B), CORM-3 (C), or $PdCl_2$ (D) at 37 ℃ for 8 h. Data as mean values ± SD (n = 6).

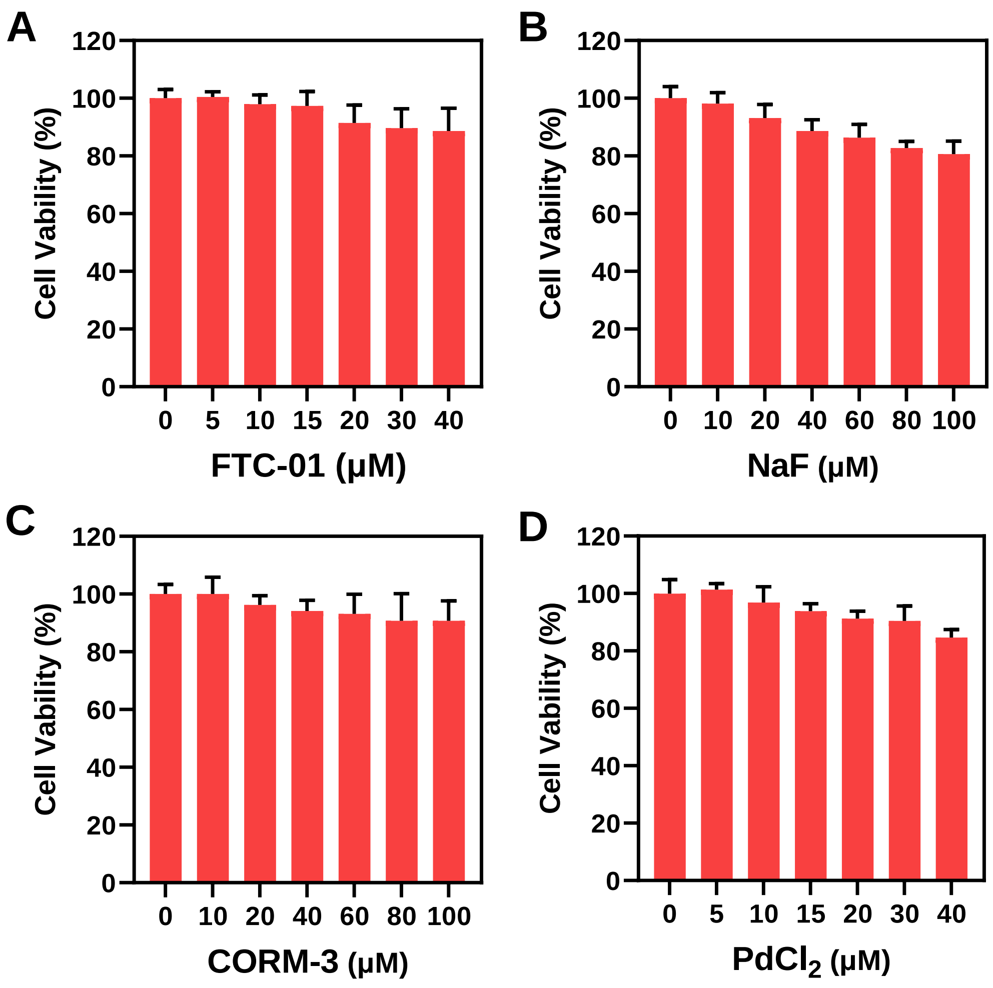

Figure S5. Effects of compounds on cell viability (CCK-8 method). HeLa cells were treated with **FTC-01** (A), NaF (B), CORM-3 (C), or $PdCl_2$ (D) at 37 ℃ for 8 h. Data as mean values ± SD (n = 6).

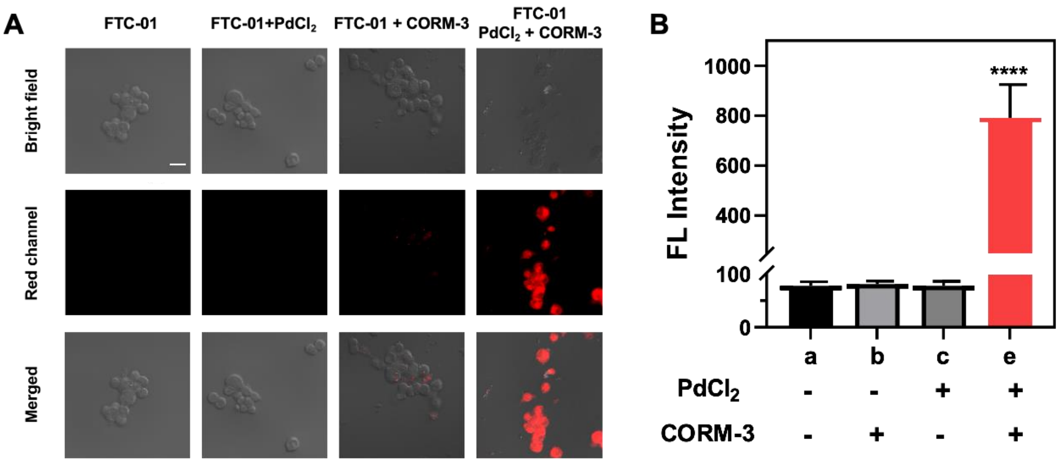

Figure S6. (A) Confocal fluorescence images of HepG2 cells treated with **FTC-01** (10 μM), $PdCl_2$ (10 μM) and CORM-3 (100 μM) for 30 min. (B) Quantification of the FL intensities in (A). Red channel: λ_{ex} = 555 nm, λ_{em} = 685−760 nm. Scale bar = 20 μm. Data as mean values ± SD (n = 6).

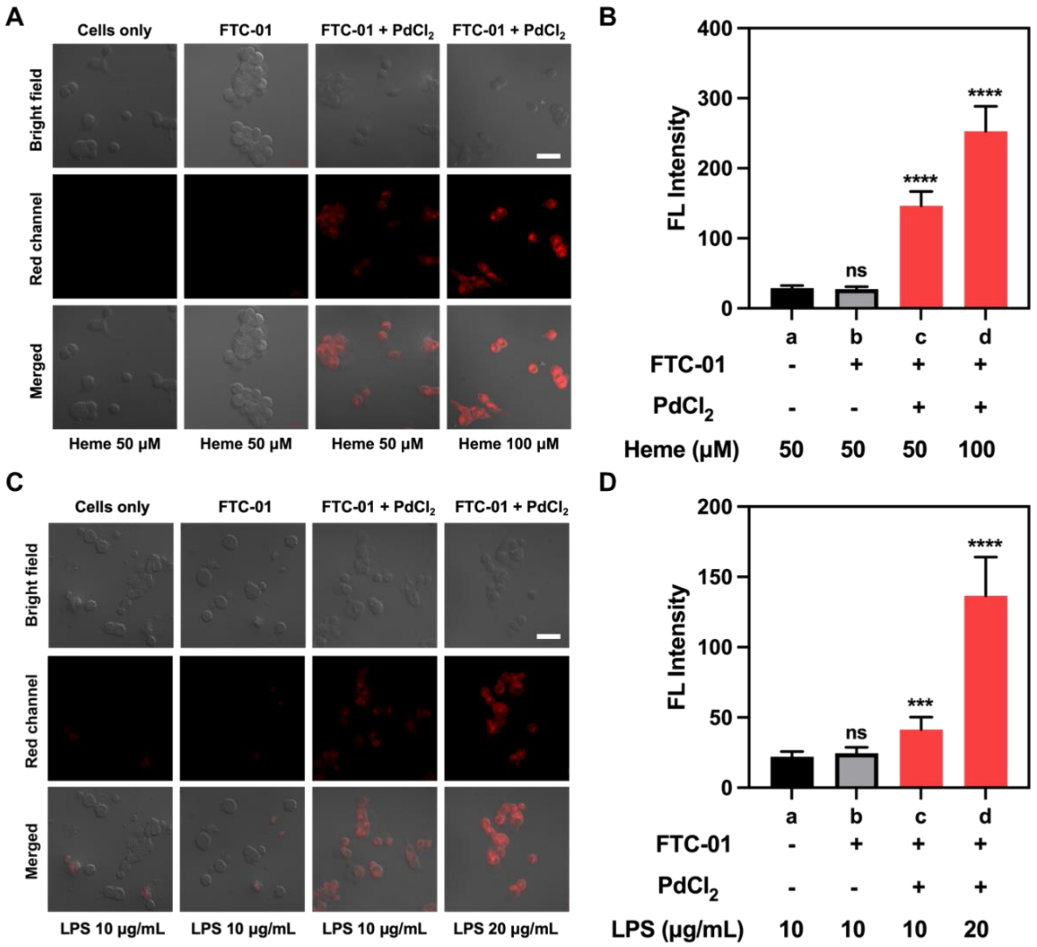

Figure S7. Confocal fluorescence images of HepG2 cells (A, C) pretreated with different concentrations of Heme or LPS for 12 h and then incubated with different treatments: (a) cells only; (b) **FTC-01** (10 μM) for 30 min; (c, d) **FTC-01** (10 μM) + $PdCl_2$ (10 μM) for 30 min. (B, D) Quantification of the fluorescence intensities in (A, C). Red channel: λ_{ex} = 555 nm, λ_{em} = 685−760 nm. Scale bar = 20 μm. Data as mean values ± SD (n = 6).

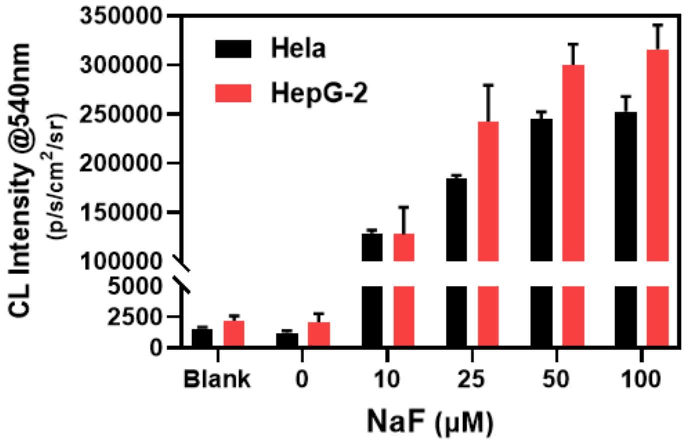

Figure S8. Chemiluminescence intensity of **FTC-01** (10 μM) in HeLa cells (5×10^4/well) and HepG2 cells (5×10^4/well) with different concentrations of NaF. Data as mean values ± SD (n = 6).

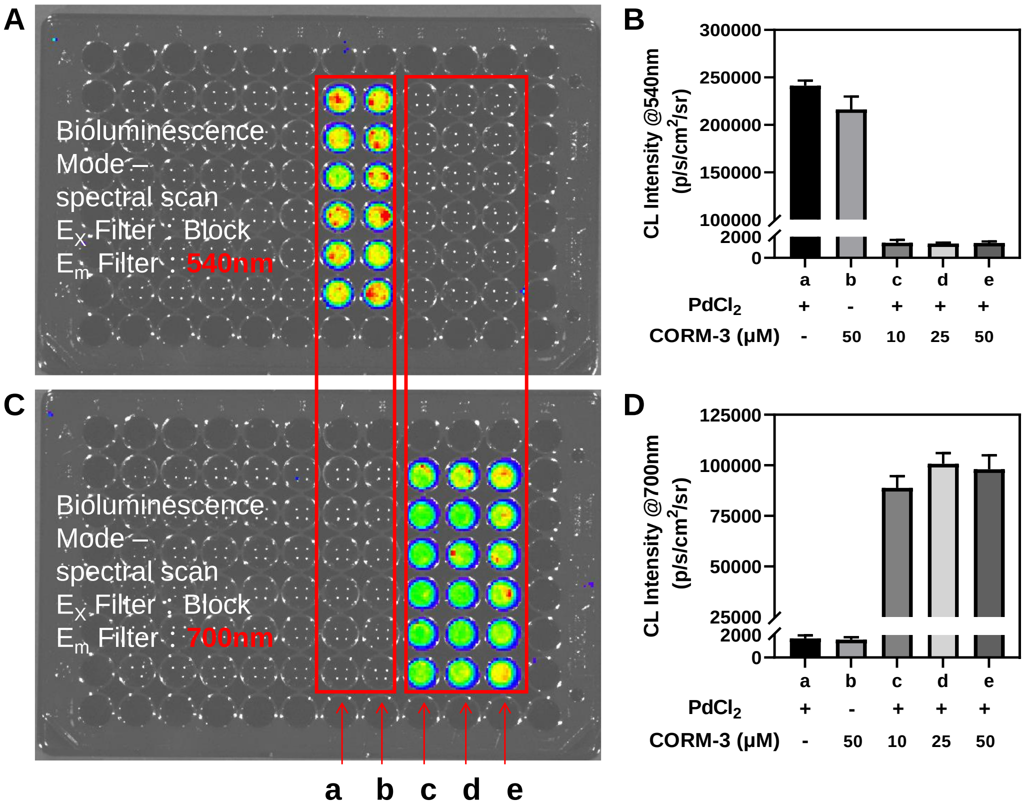

Figure S9. (A, C) CL images of HeLa cells taken from the same 96 well plate under the action of emission filters with different wavelengths. Group a: $PdCl_2$ (10 μM) was added to pre incubate for 1h. Group b: CORM-3 (50 μM) was added to pre incubate for 1h. Group c: $PdCl_2$ (10 μM) and CORM-3 (10 μM) were added for pre incubation for 1h. Group d: $PdCl_2$ (10 μM) and CORM-3 (20 μM) were added for pre incubation for 1h. Group e: $PdCl_2$ (10 μM) and CORM-3 (50 μM) were added for pre incubation for 1h. NaF (50 μM) was subsequently added to each group, and CL imaging was performed at 540 nm and 700 nm using bioluminescence mode spectral scanning, respectively. The above concentrations are the final concentration of each substance in the hole. (B, D) Quantification of the CL intensities in (A, C). Data as mean values ± SD (n = 6).

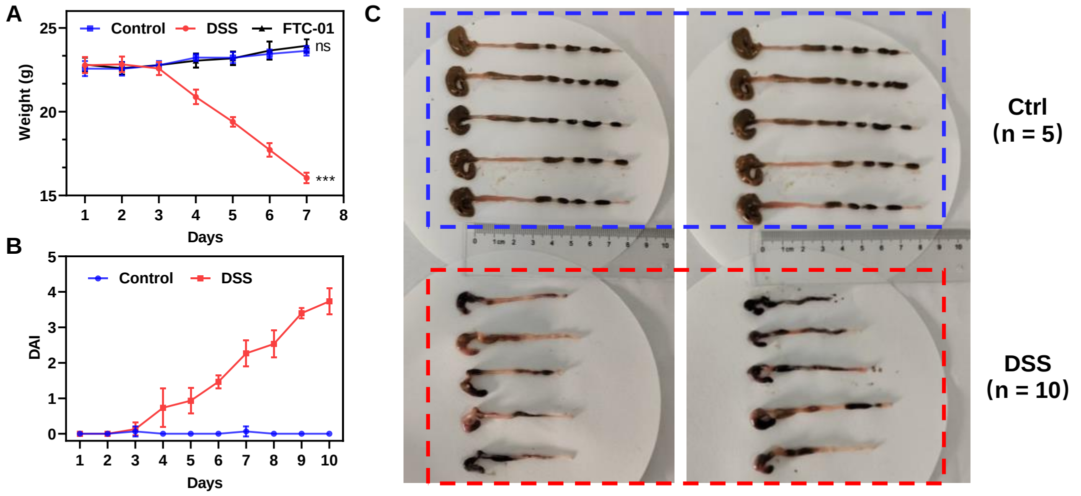

Figure S10. (A) Changes in the average body weight of each group of mice. (B) Daily disease activity measurements encompassing weight, stool consistency and the presence of blood were assessed for each group of mice. Data as mean values ± SD (n = 5 for control, n = 10 for DSS group); ns: not significant, *** P < 0.001. (C) Observation of the colon characteristics of mice in each group on the 10th day of DSS administration.

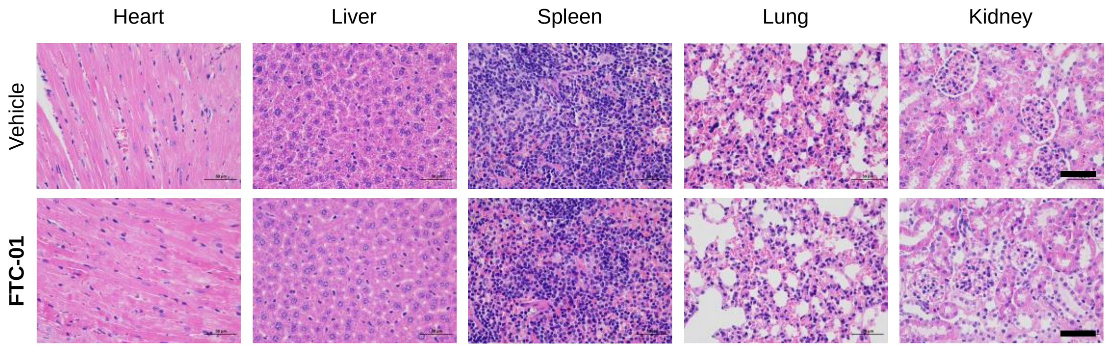

Figure S11. Safety evaluation of probe **FTC-01** in mice. Representative H&E staining images (400× magnification; scale bar: 50 μm) of major organs from vehicle- and probe-treated mice.

Procedure: For acute toxicity assessment, male C57 mice (n = 3 per group) were randomly allocated to two groups: one vehicle control and one group dosed with **FTC-01** (daily, 40 mg/kg, i.p., a dose ten times higher than the typical dosage for probe application). Survival and body weight were monitored daily for 14 days. Following the observation period, major tissues (heart, liver, spleen, lungs, kidneys) were harvested for histopathological analysis with H&E staining.

Results: Histopathological analysis of major organs (heart, liver, spleen, lungs, kidneys) revealed no pathological alterations following intraperitoneal administration of **FTC-01** at 40 mg/kg.

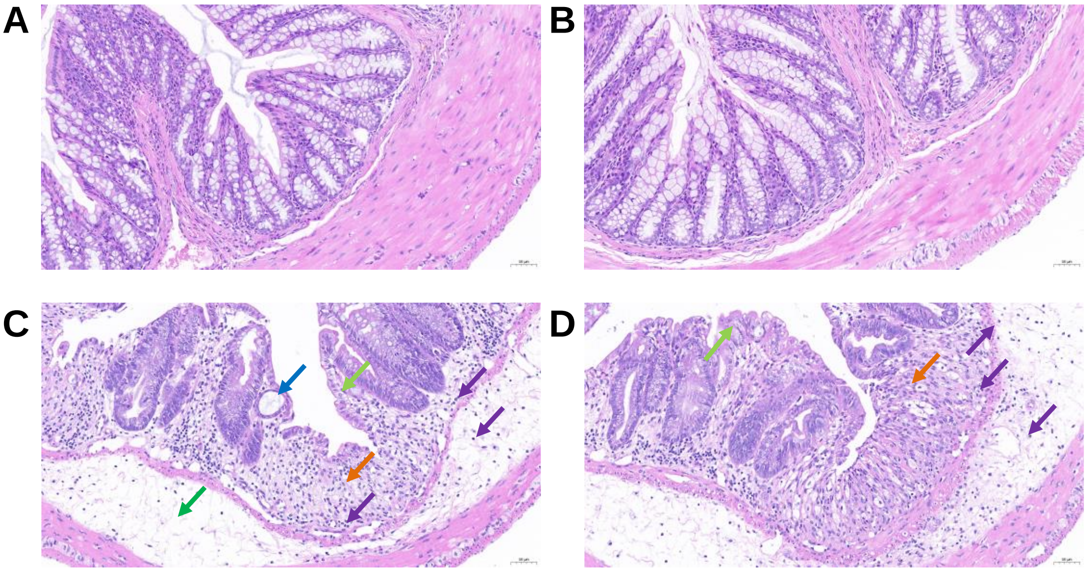

Figure S12. Representative micrographs of H&E staining in paraffin embedded intestinal sections of (A, B) normal mice and mice after DSS treatment for 10 days (C, D). Scale bar = 50 μm.

Representative microscopic images of H&E staining in paraffin-embedded intestinal sections of mice after 10 days of DSS treatment showed extensive ulcers in the intestinal tissue (orange arrows), disappearance of the mucosal epithelial cells and intestinal glands, replaced by connective tissue hyperplasia, accompanied by a large number of lymphocytes and granulocytes infiltration (purple arrows), with inflammatory cell infiltration into the submucosa; a small amount of mucosal epithelial cell edema (light green arrows), with loose and pale staining of the cytoplasm; the remaining intestinal glands in the lamina propria were large in volume, with few goblet cells, and occasional intestinal gland dilation (blue arrows), with flattened intestinal gland epithelium; extensive edema in the submucosa (green arrows), with loose arrangement of connective tissue.

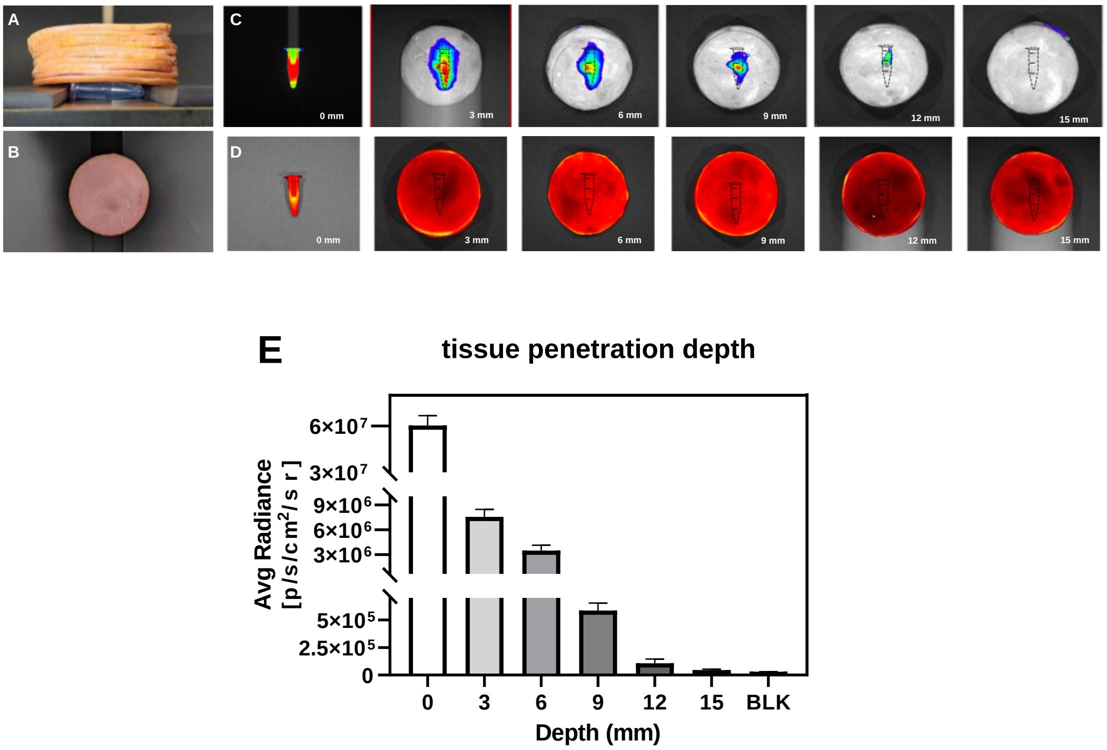

Figure S13. Tissue penetration of NIR-CL and NIR-FL imaging. (A, B) Bacon model. (C, D) NIR-CL (C) and NIR-FL (D) imaging at different tissue thicknesses. (E) NIR-CL intensity at different tissue thicknesses.

The experimental results comparing the tissue penetration depth between the NIR-CL channel and the NIR-FL channel are quite remarkable. In the NIR-CL channel, the deepest signal response exceeds 10 mm, and even at a thickness of 12 mm, there is still more than double the signal response. In contrast, the NIR-FL channel shows almost no significant fluorescence signal transmission, likely due to the strong background fluorescence from the ham slices (used to simulate tissue), resulting in an inability to accurately quantify the signal intensity.

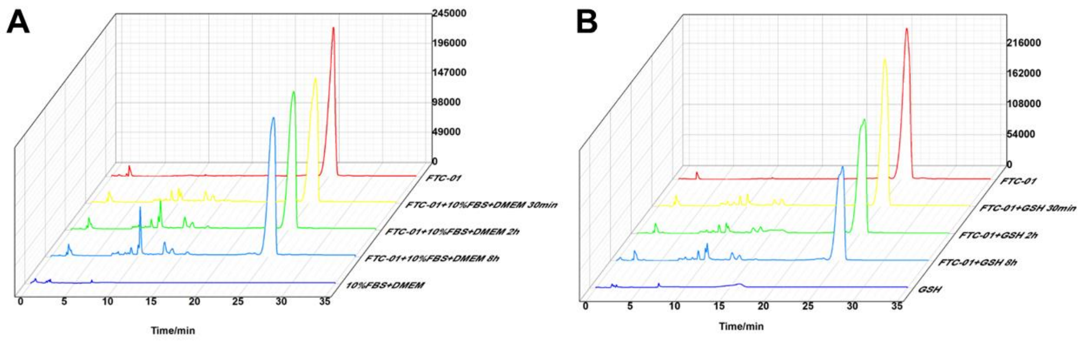

Figure S14. HPLC analysis of **FTC-01** stability under different conditions. (A) Stability of FTC-01 (5 mM) in 10% FBS-supplemented DMEM at 37°C. (B) Stability of FTC-01 (5 mM) in DMEM containing 5 mM glutathione (GSH) at 37°C. Samples were prepared as described and analyzed by HPLC at indicated time points.

5. NMR and HRMS spectra

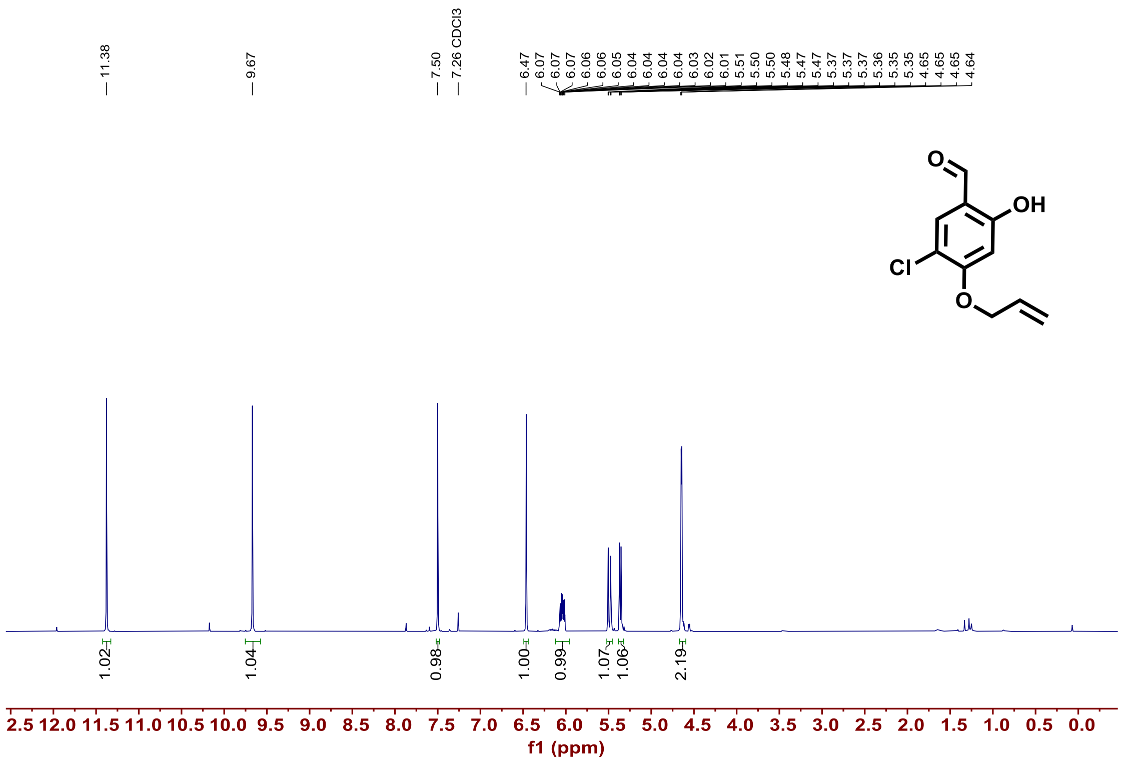

^{1}H NMR of Compound **1**

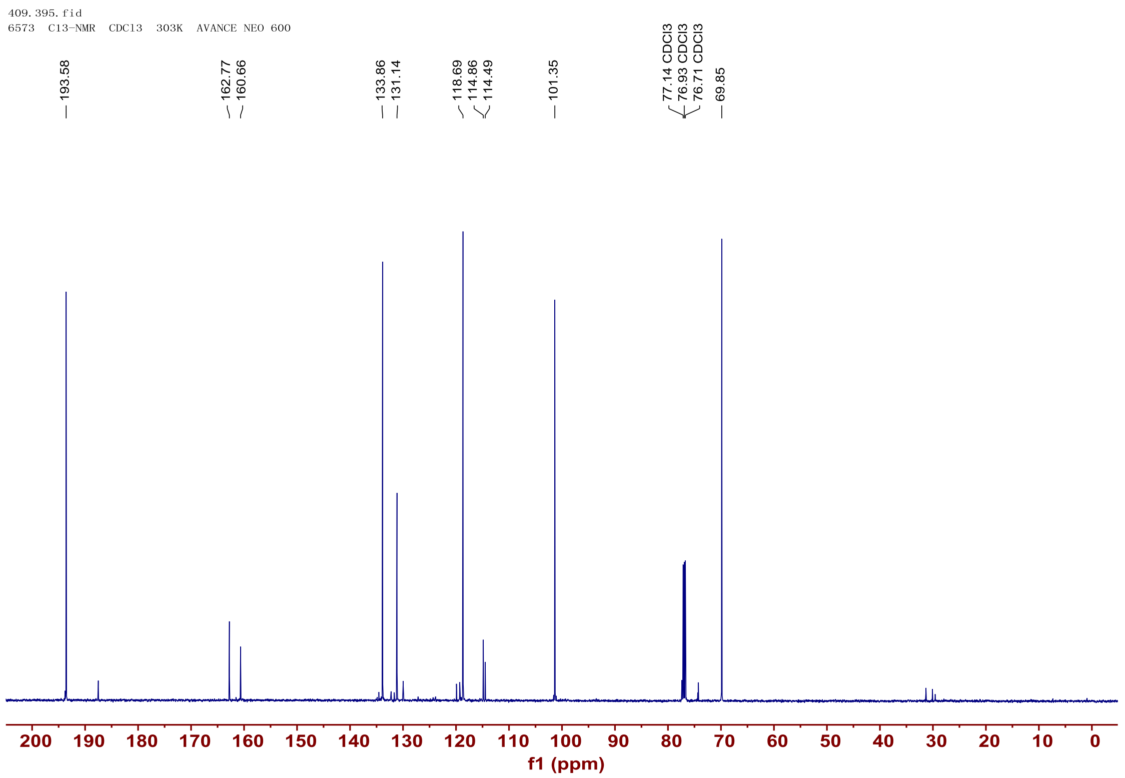

^{13}C NMR of Compound **1**

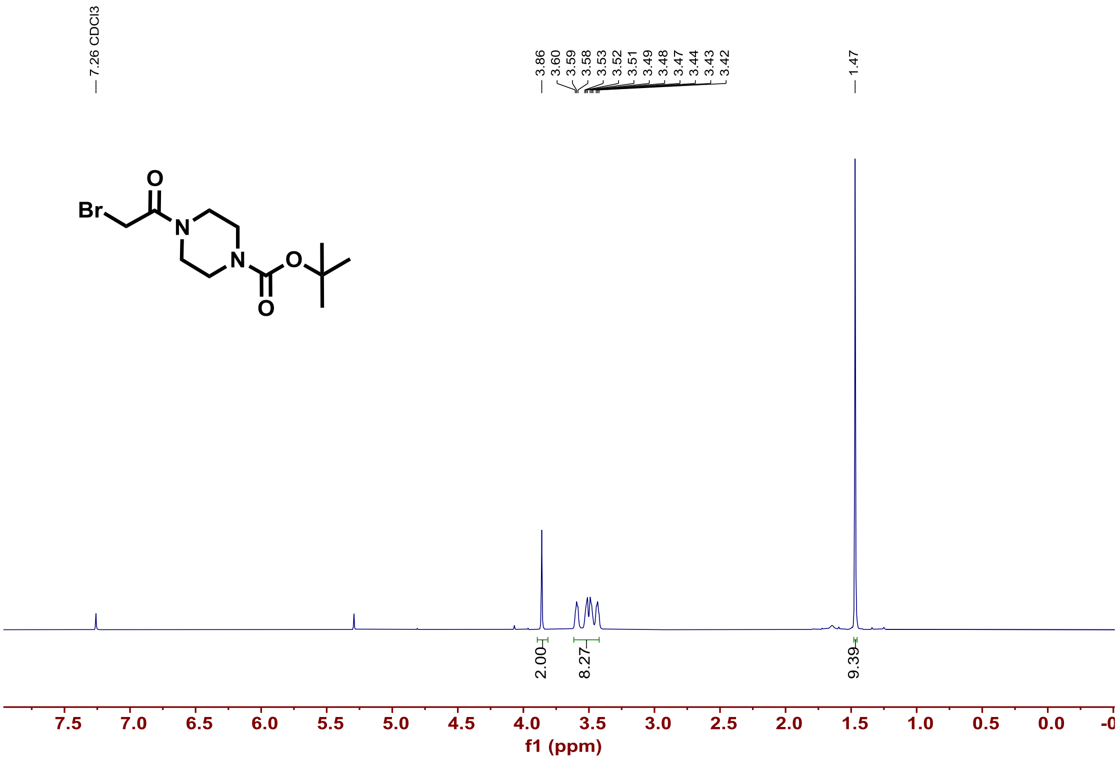

^{1}H NMR of Compound **2**

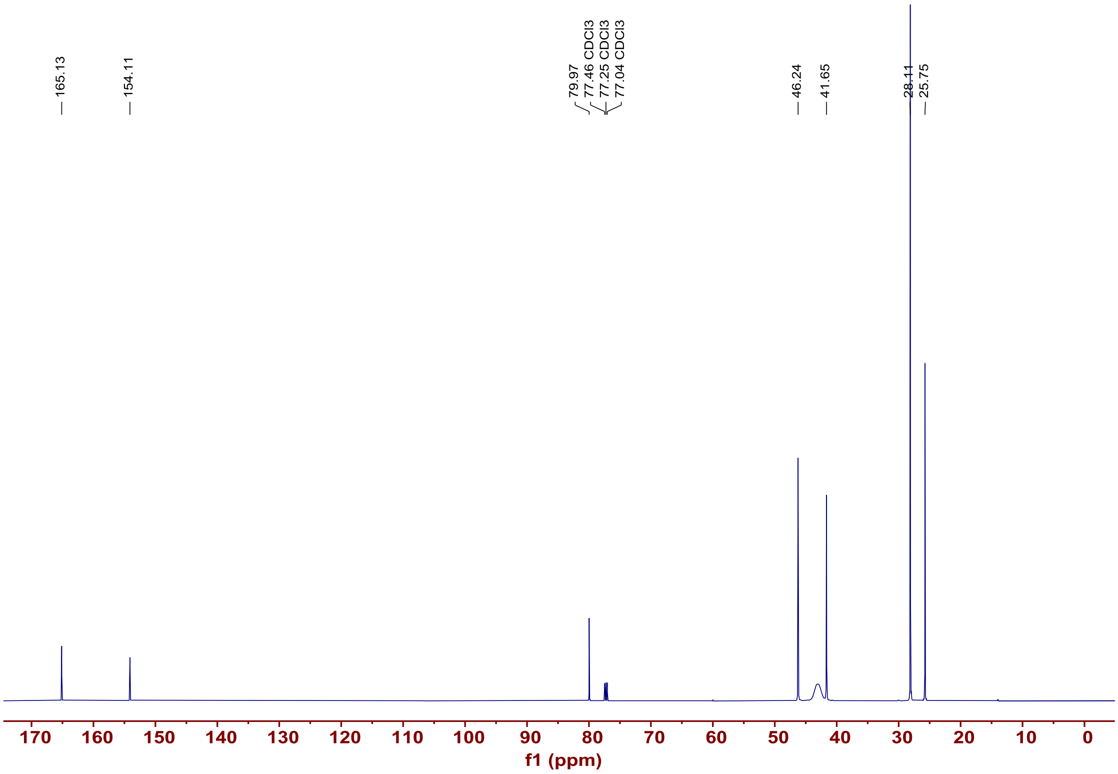

^{13}C NMR of Compound **2**

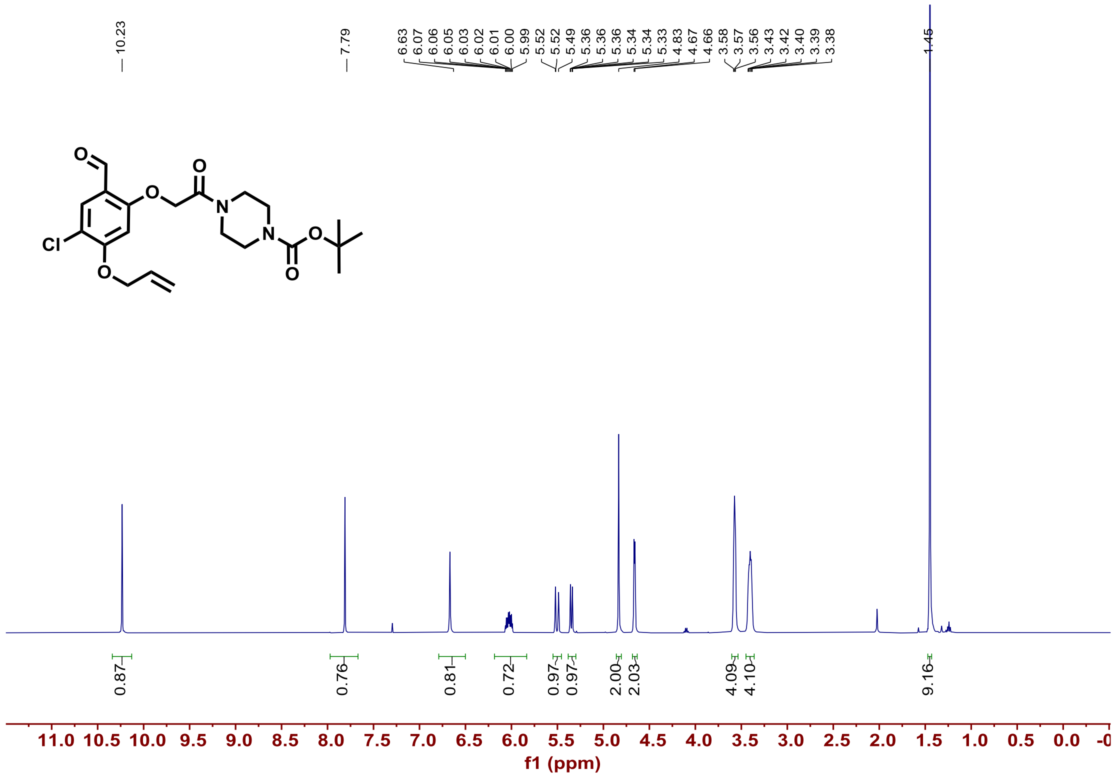

^{1}H NMR of Compound **3**

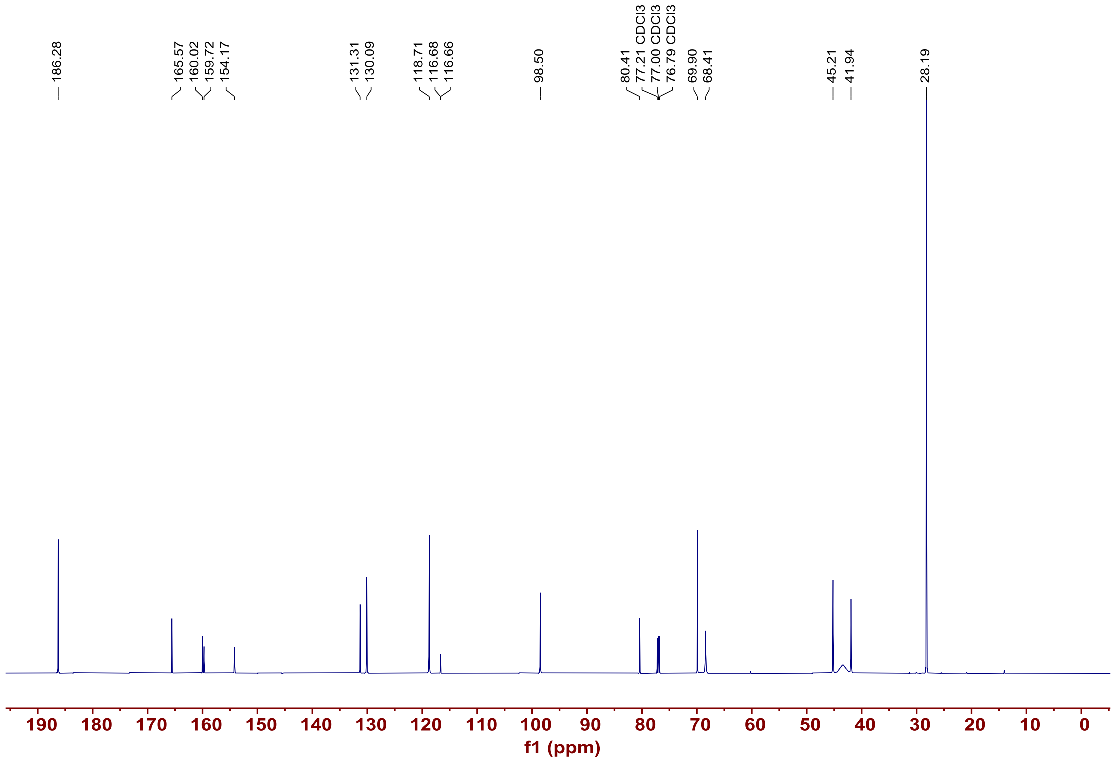

^{13}C NMR of Compound **3**

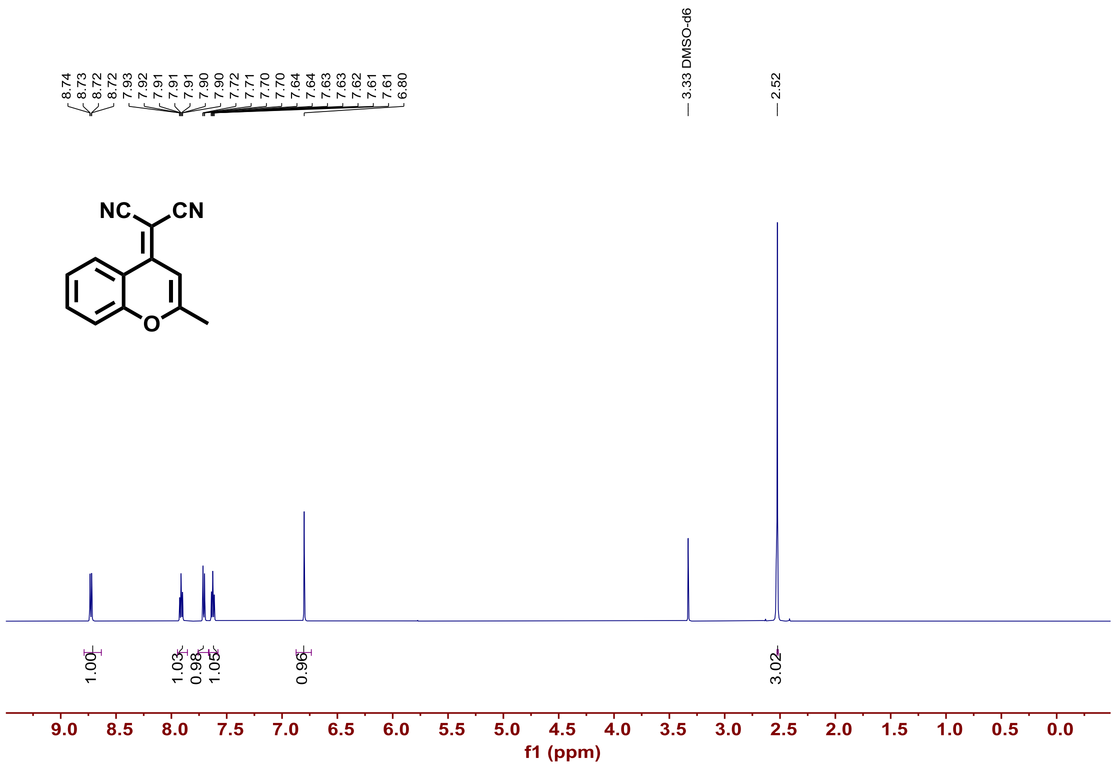

^{1}H NMR of Compound **4**

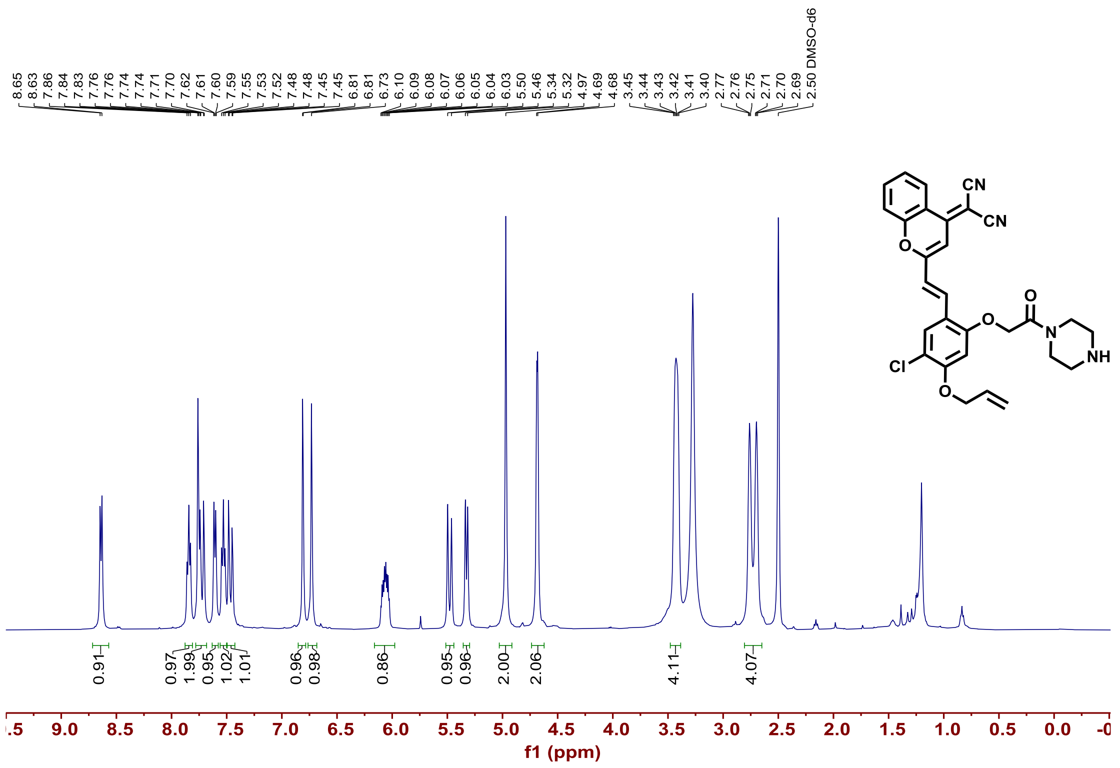

^{1}H NMR of Compound **6**

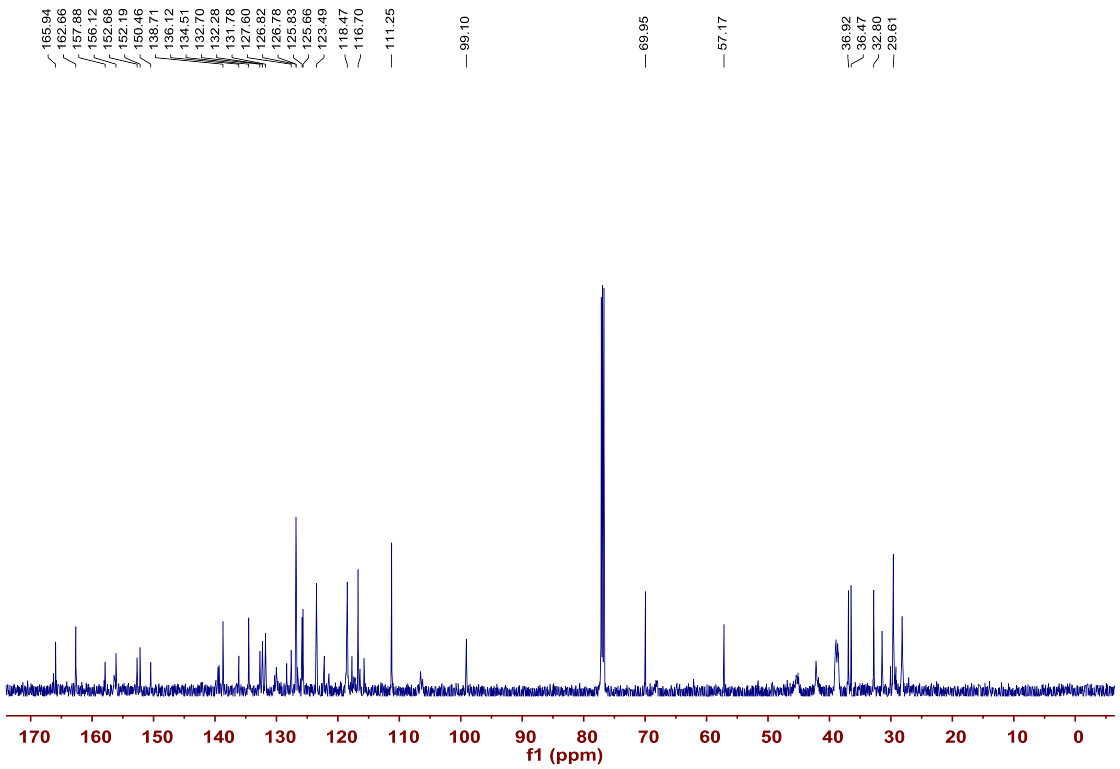

^{13}C NMR of Compound **6**

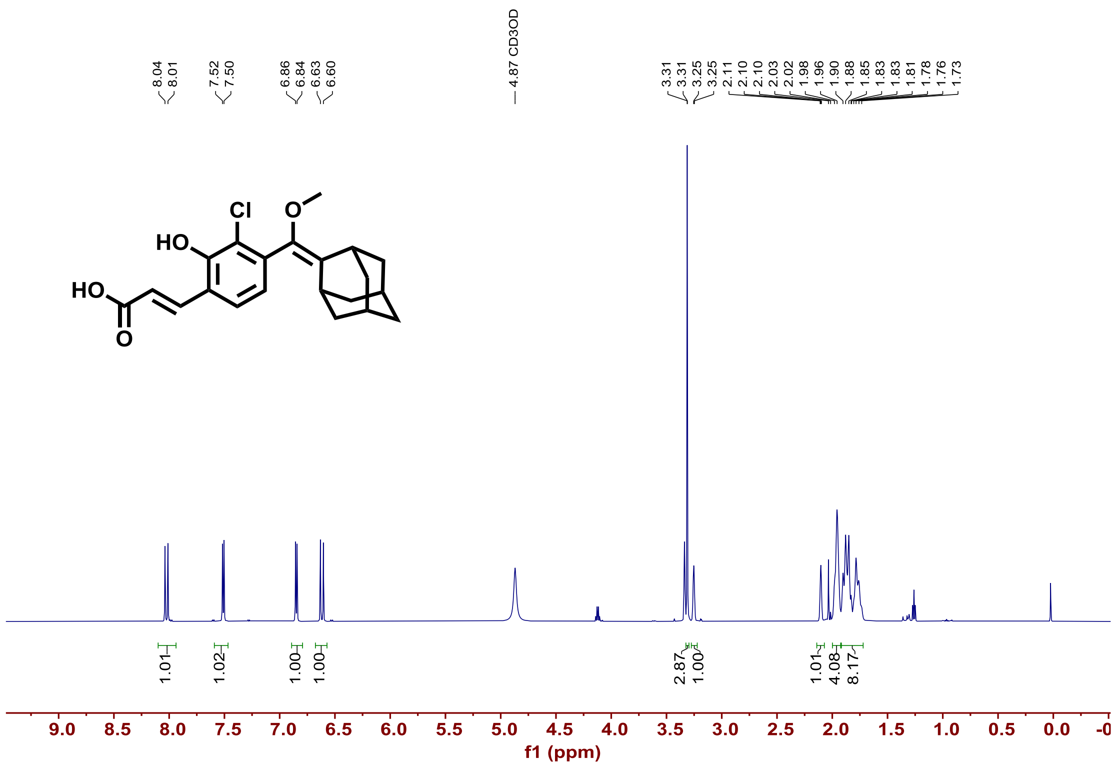

^{1}H NMR of Compound **7**

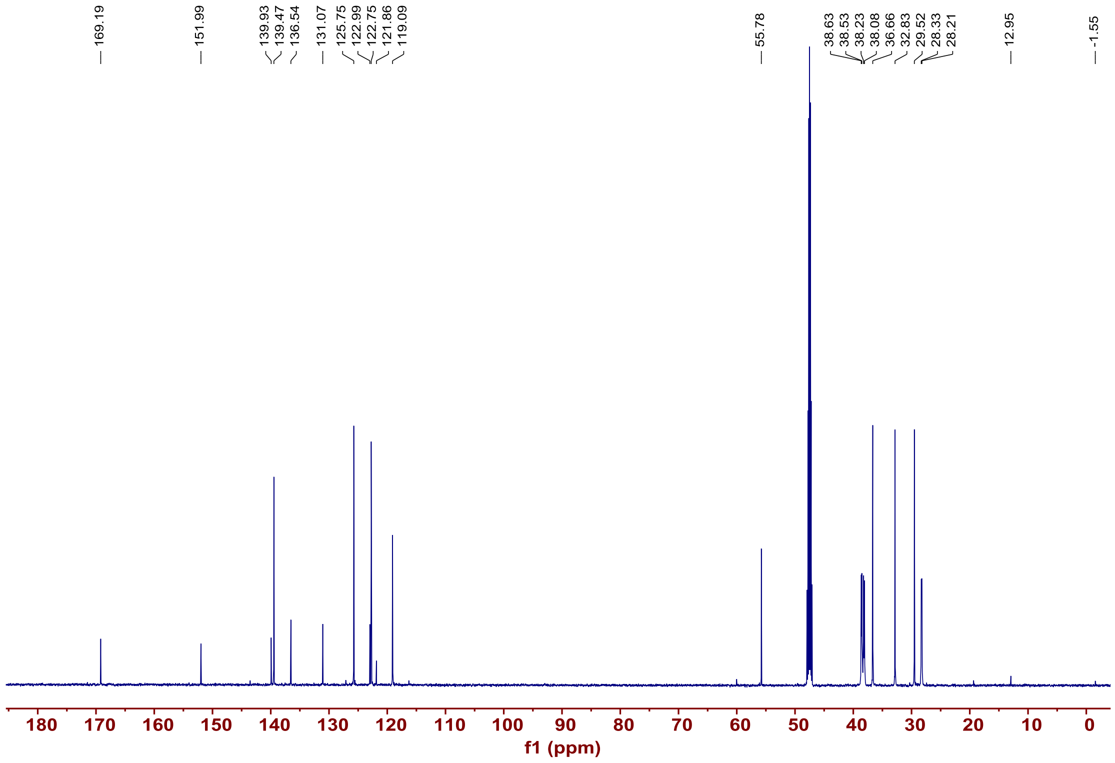

^{13}C NMR of Compound **7**

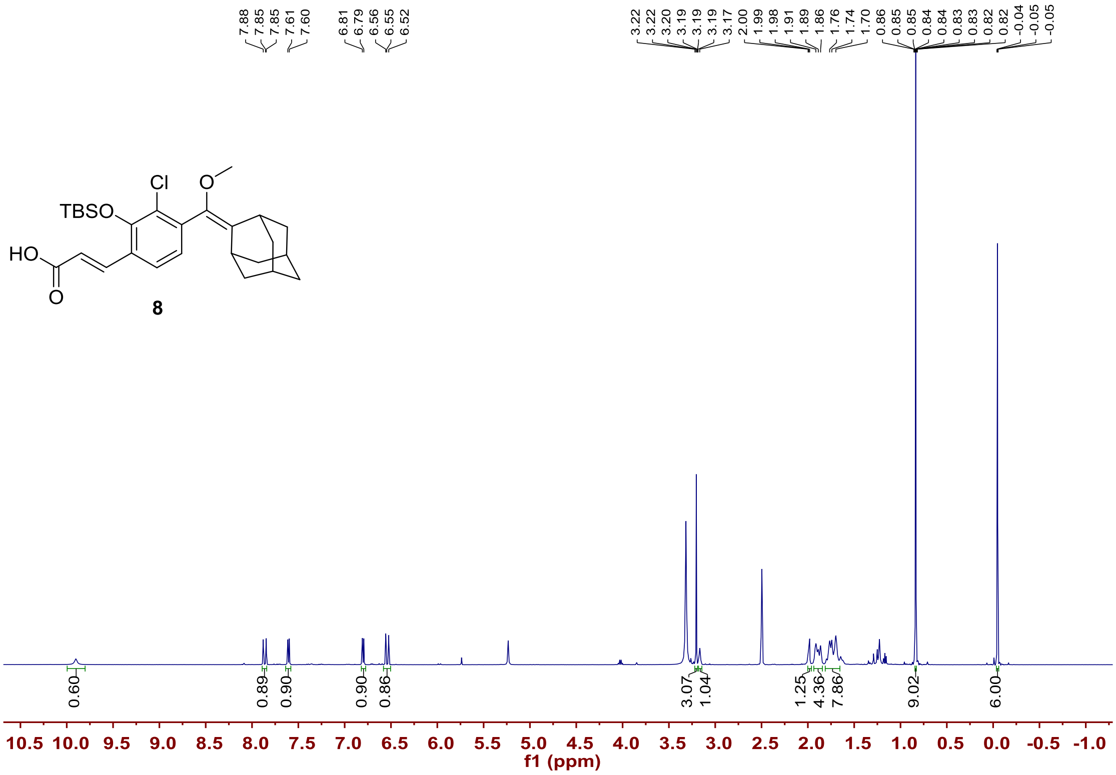

^{1}H NMR of Compound **8**

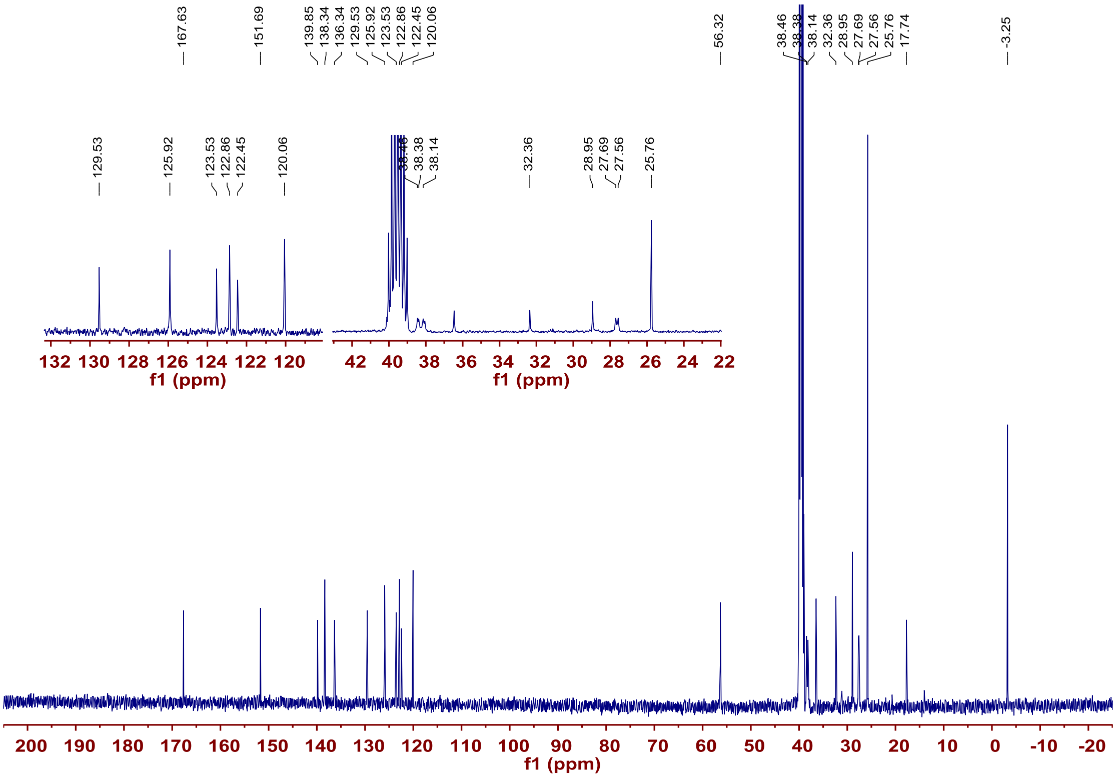

^{13}C NMR of Compound **8**

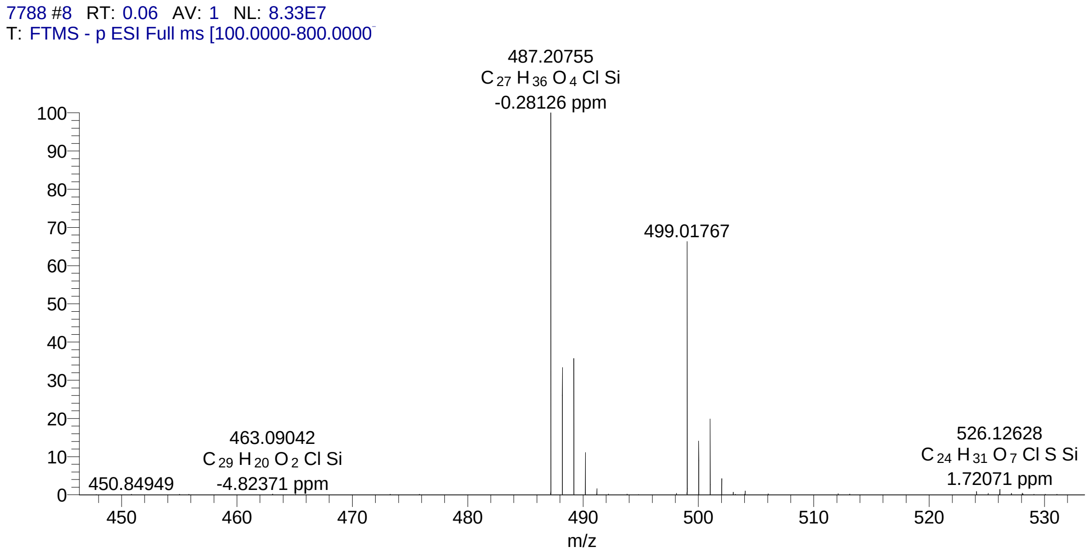

HRMS of Compound **8**

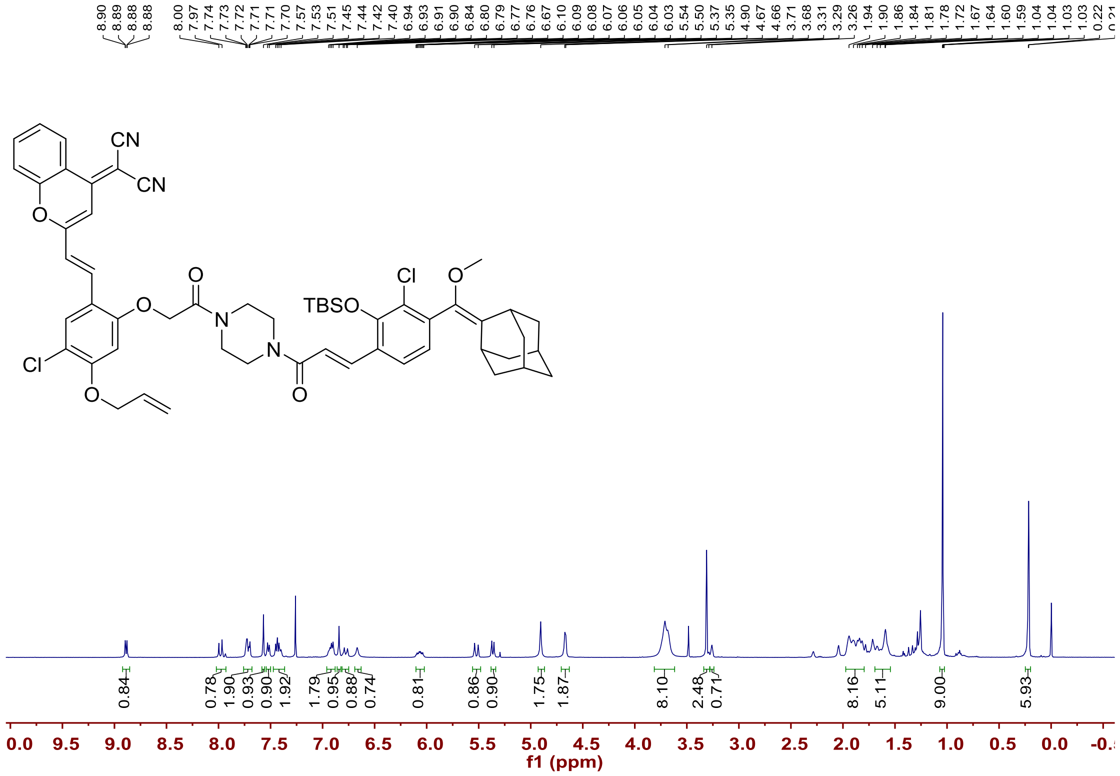

^{1}H NMR of Compound **9**

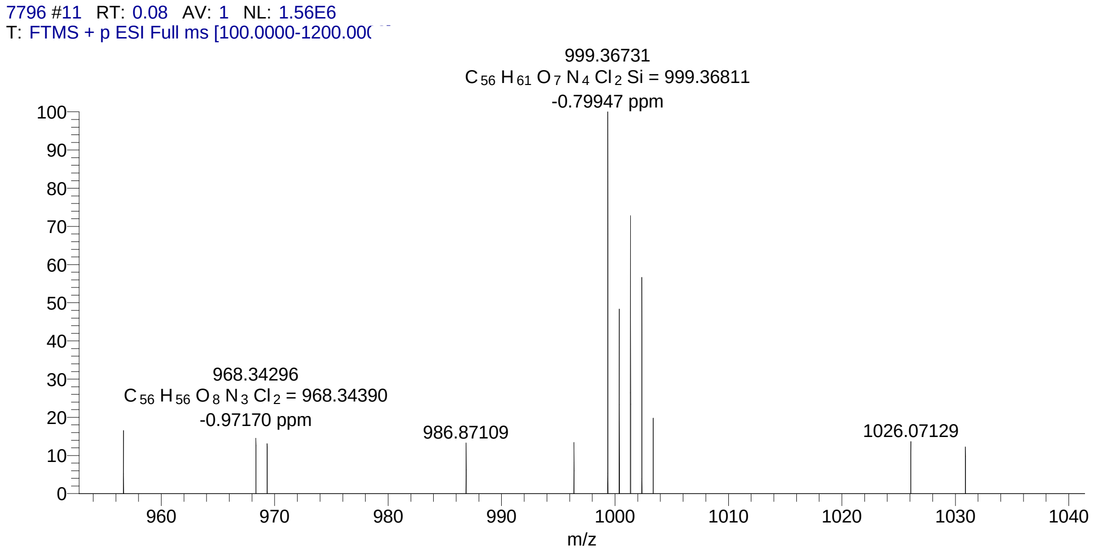

HRMS of Compound **9**

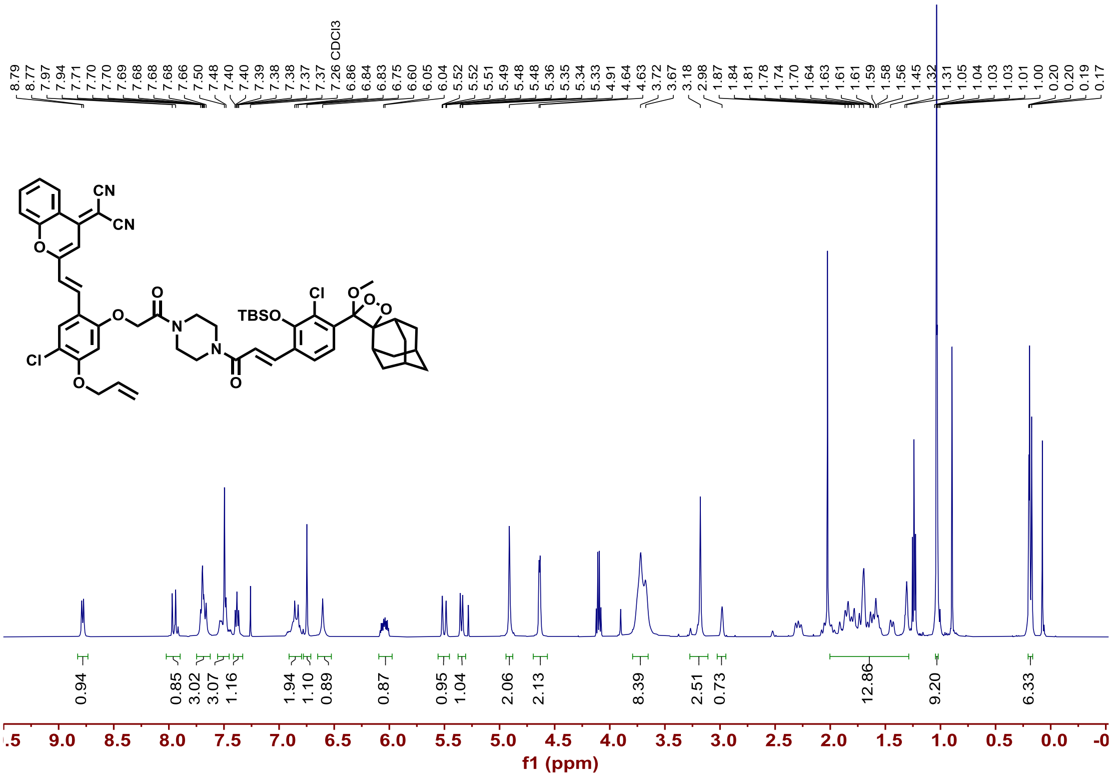

^{1}H NMR of Compound **FTC-01**

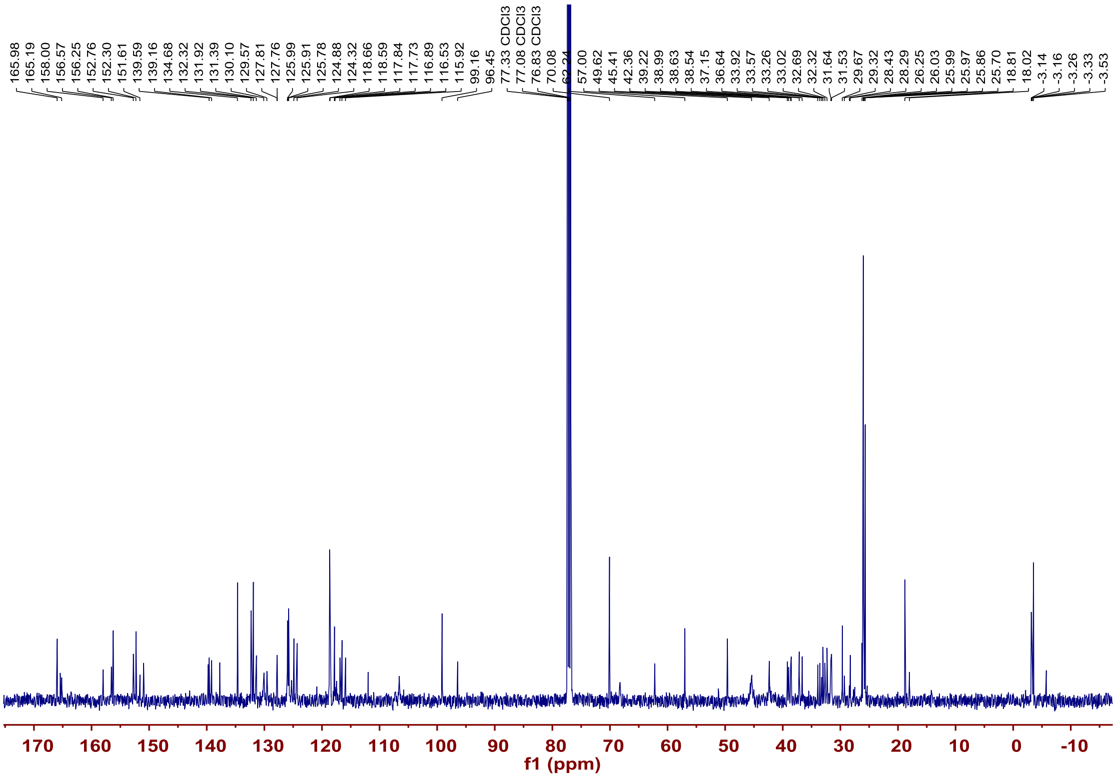

^{13}C NMR of Compound **FTC-01**

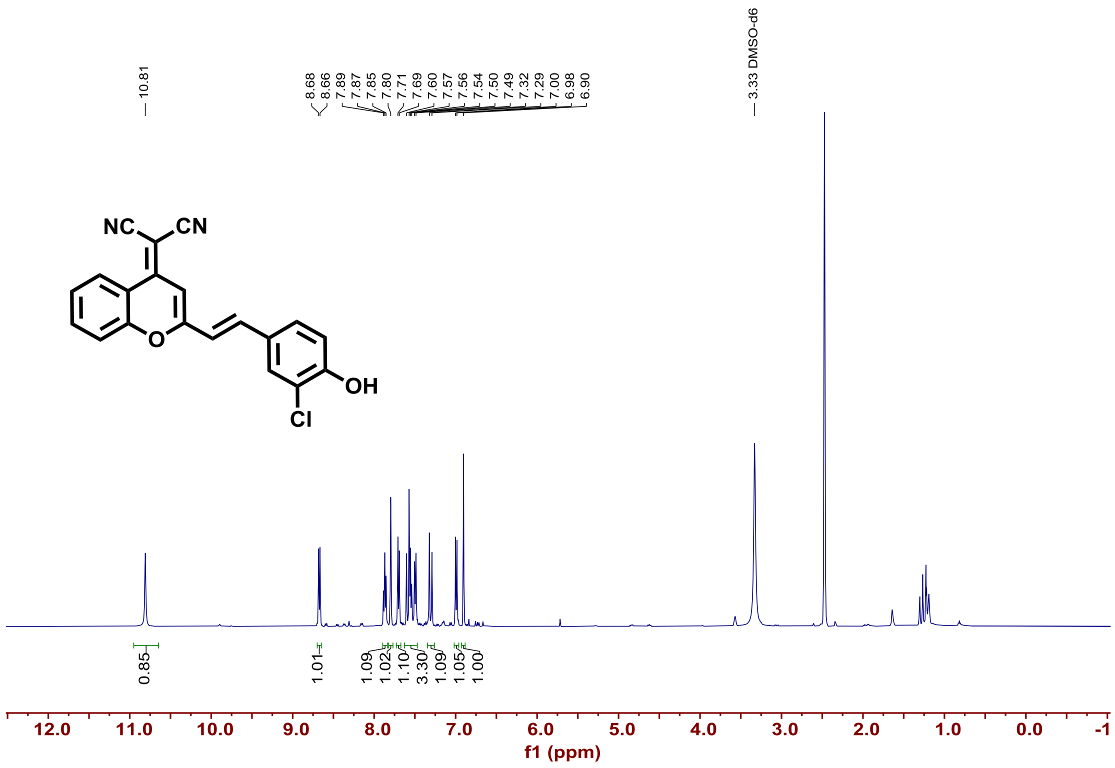

^{1}H NMR of Compound **F-01**

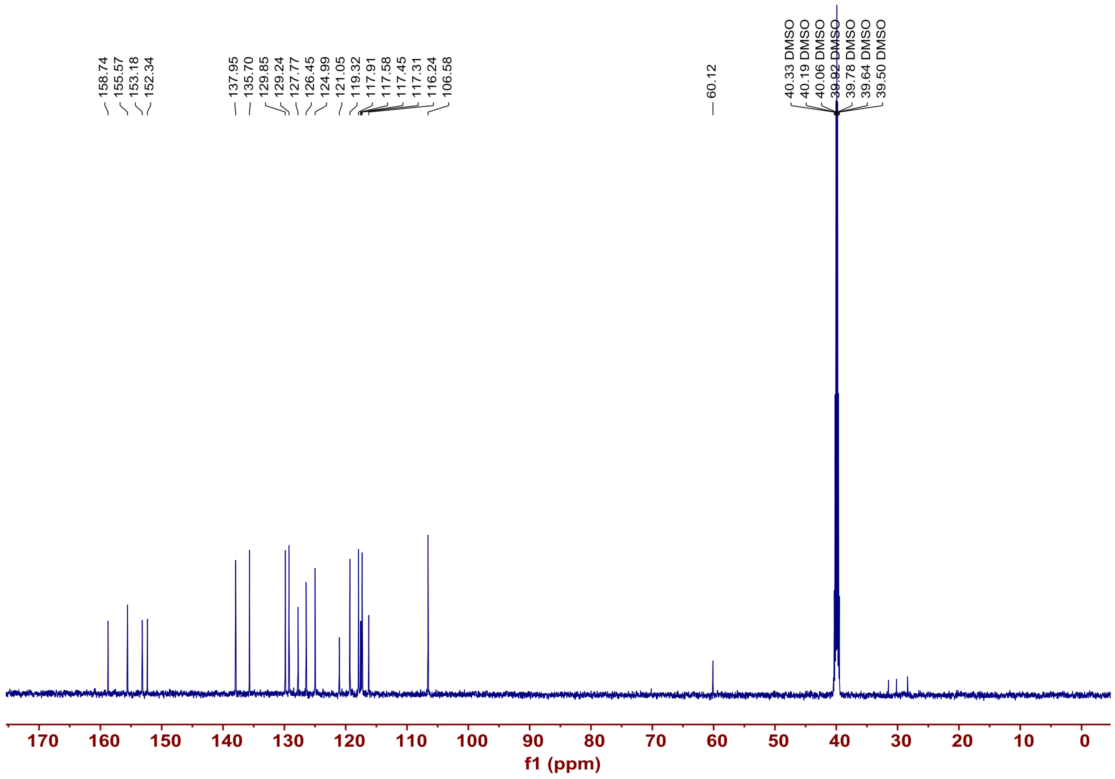

^{13}C NMR of Compound **F-01**

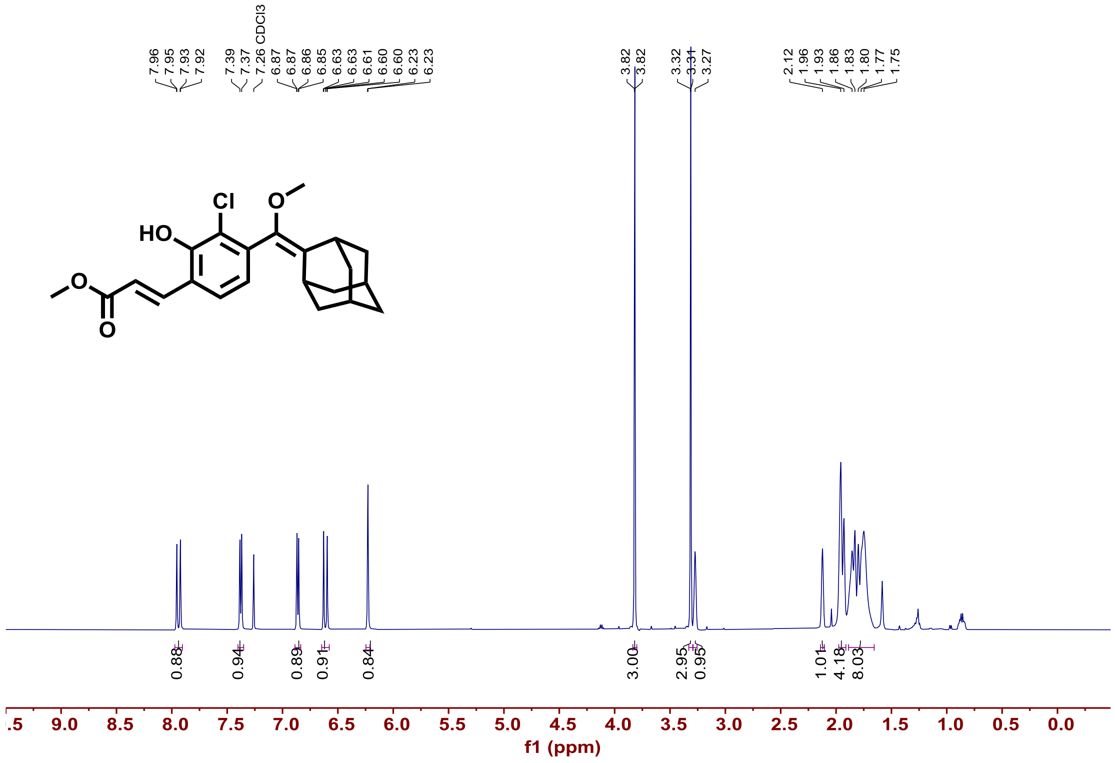

^{1}H NMR of Compound **CL**

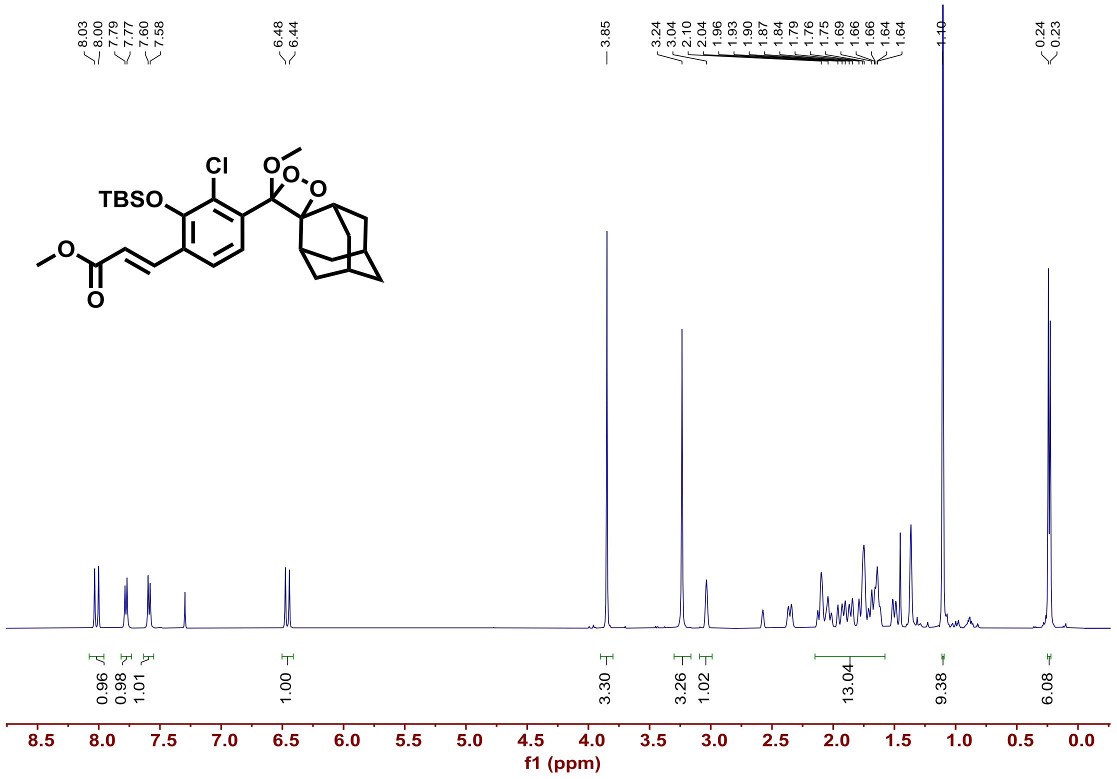

^{1}H NMR of Compound **C-01**

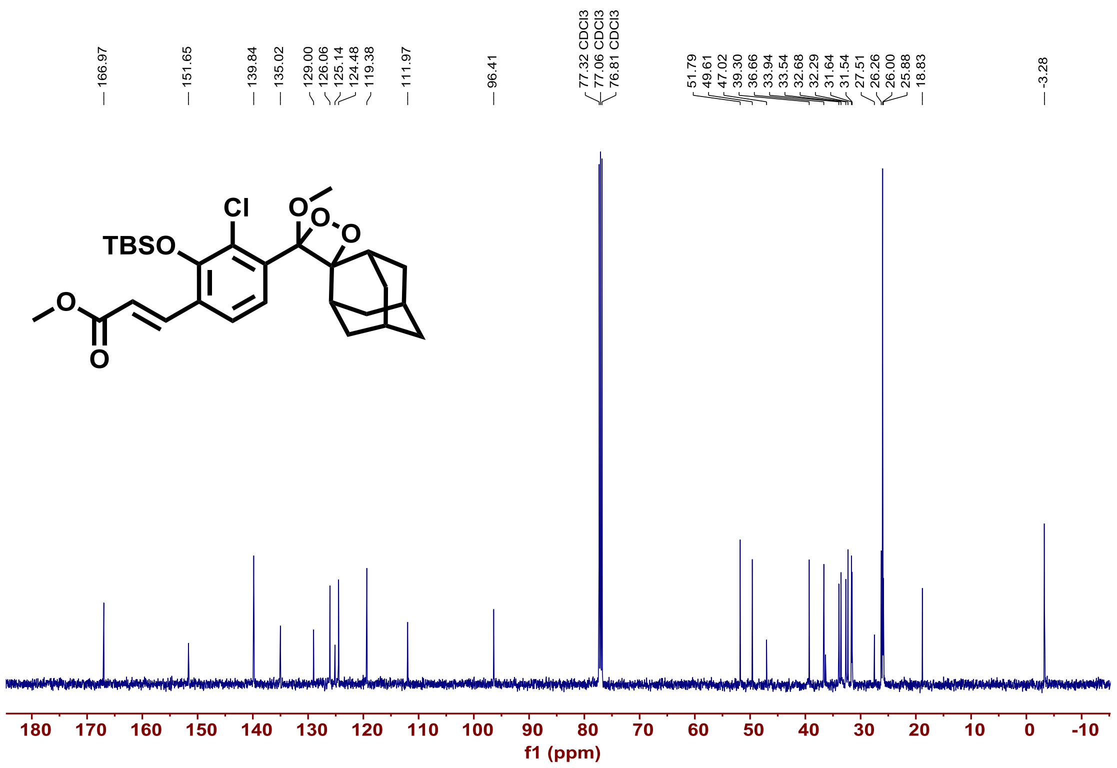

^{13}C NMR of Compound **C-01**

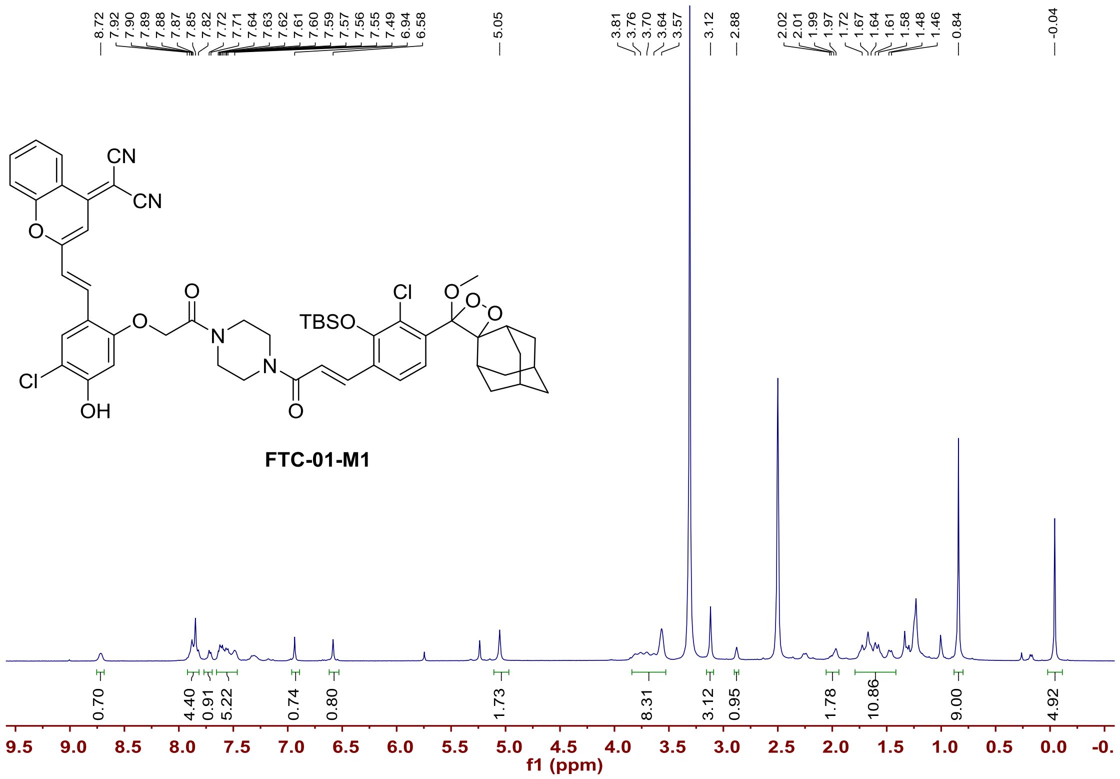

^{1}H NMR of **FTC-01-M1**

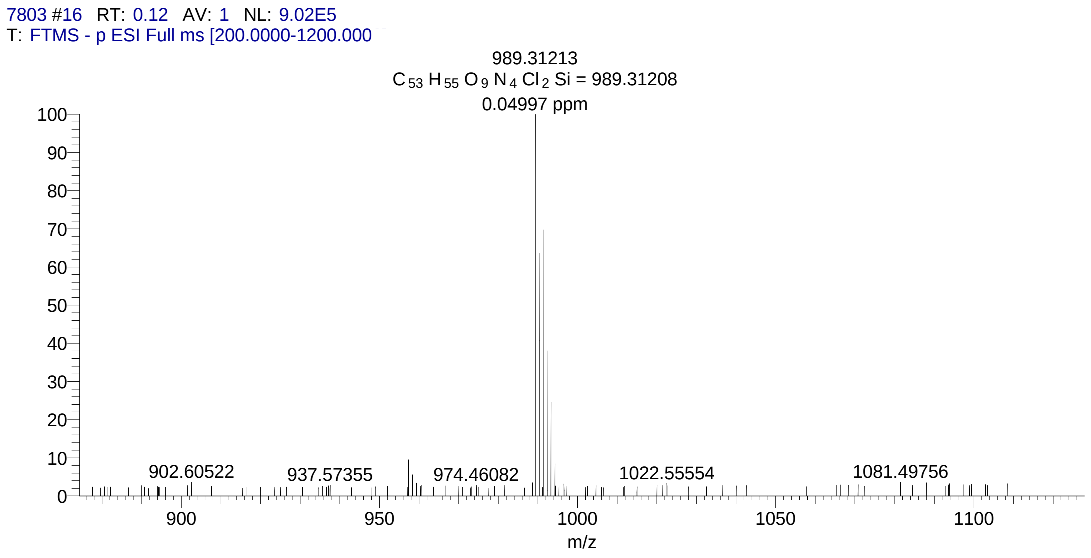

HRMS of **FTC-01-M1**

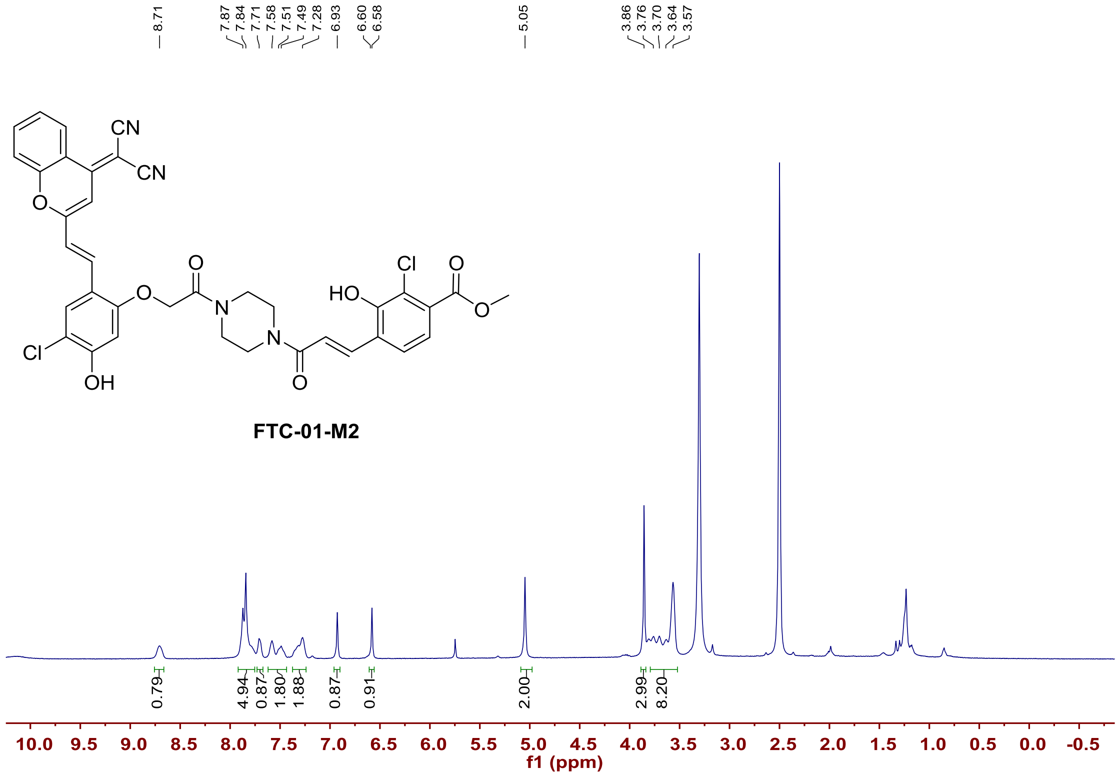

^{1}H NMR of **FTC-01-M2**

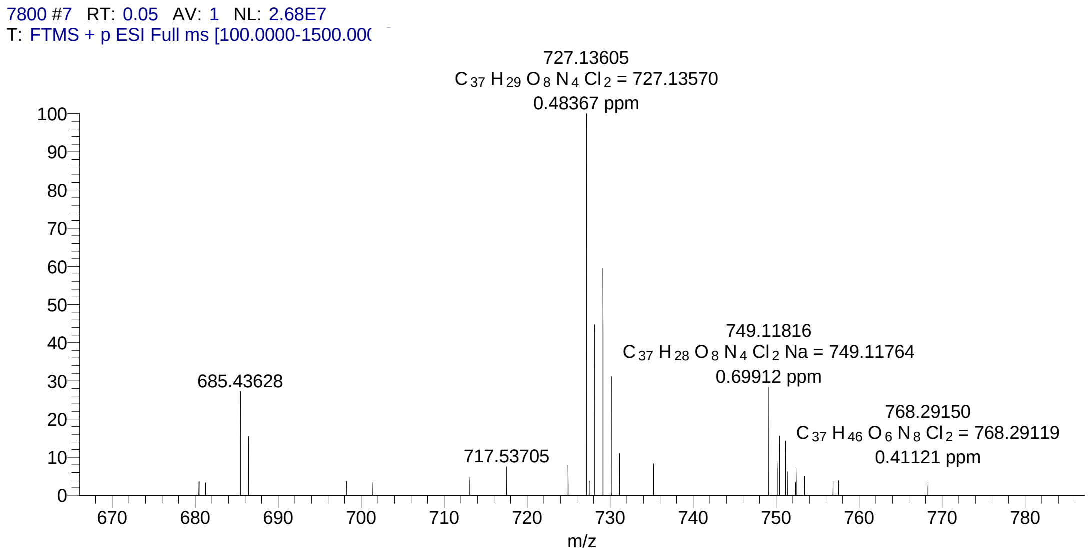

HRMS of **FTC-01-M2**

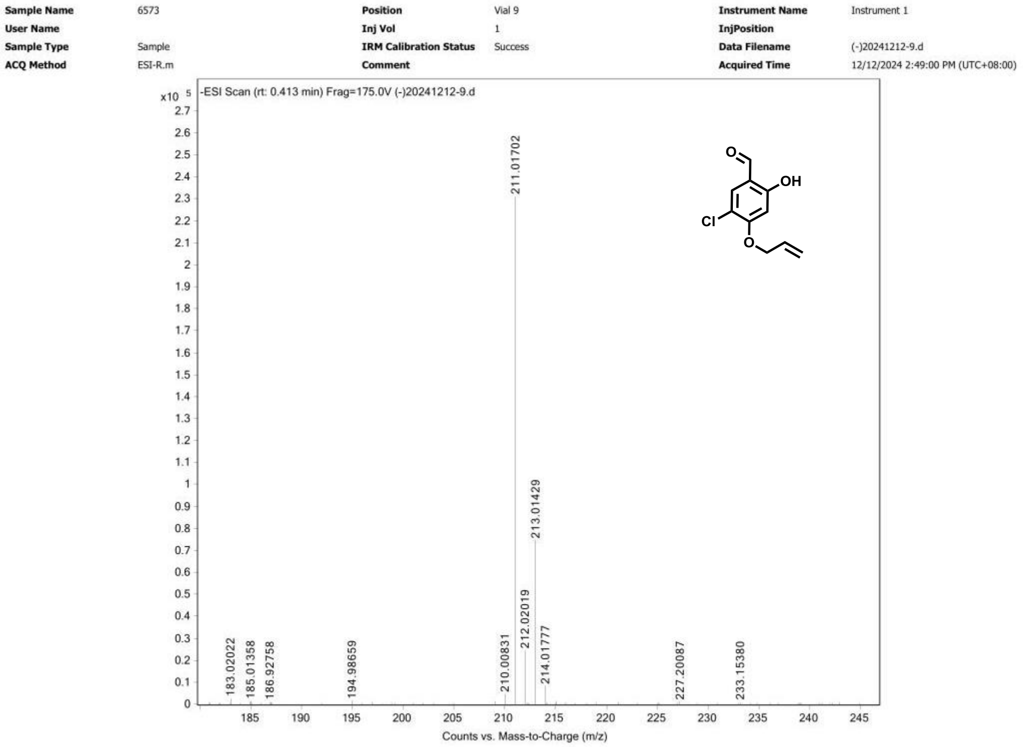

HRMS of Compound 1

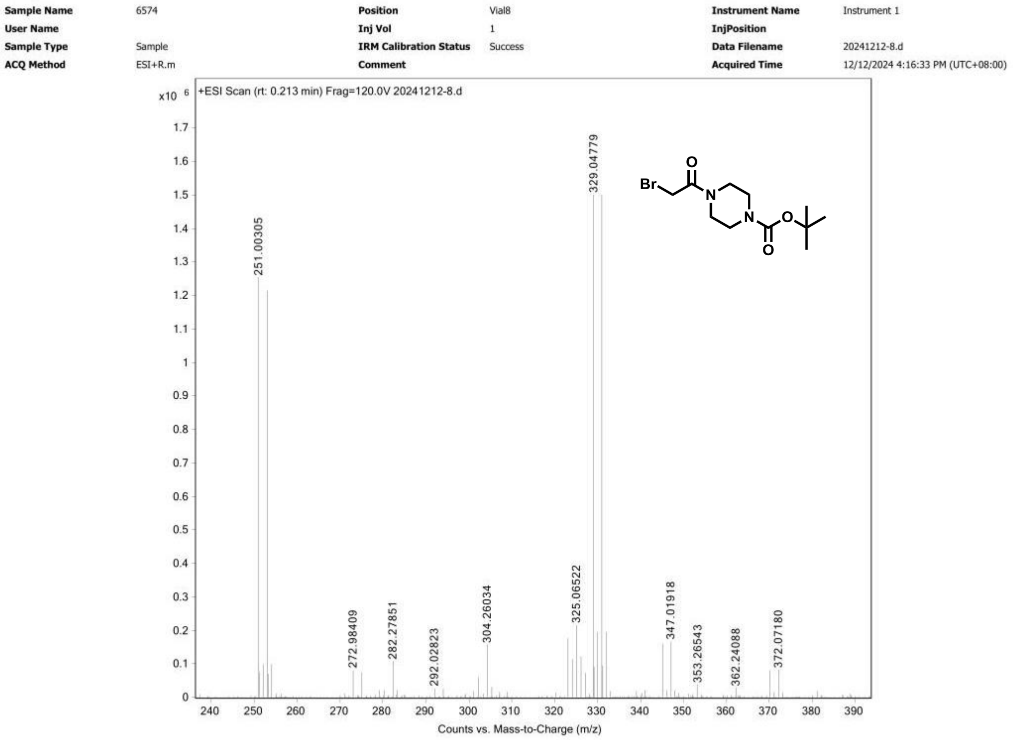

HRMS of Compound **2**

**Sample Name**	6577	**Position**	Vial7	**Instrument Name**	Instrument 1
**User Name**		**Inj Vol**	0.2	**InjPosition**	
**Sample Type**	Sample	**IRM Calibration Status**	Success	**Data Filename**	20241212-7.d
**ACQ Method**	ESI+R.m	**Comment**		**Acquired Time**	12/12/2024 12:40:06 PM (UTC+08:00)

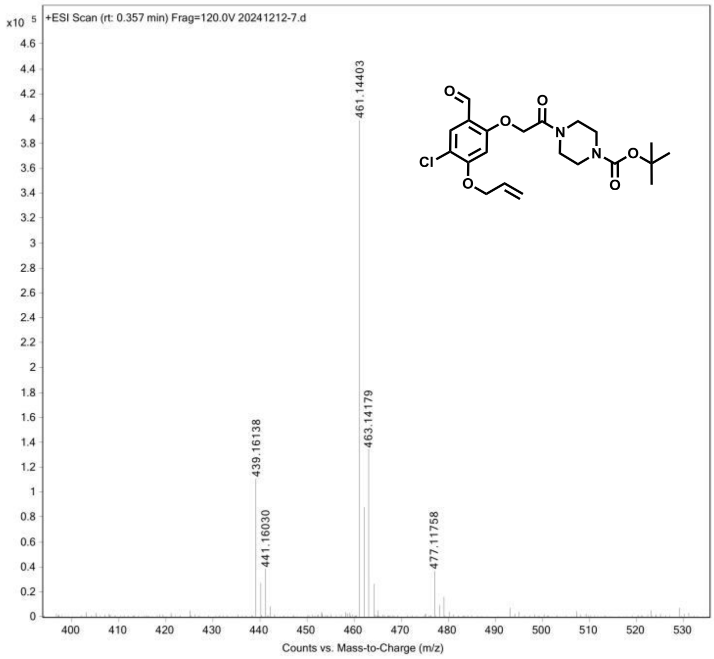

HRMS of Compound **3**

**Sample Name**	7151	**Position**	Vial5	**Instrument Name**	Instrument 1
**User Name**		**Inj Vol**	0.2	**InjPosition**	
**Sample Type**	Sample	**IRM Calibration Status**	Success	**Data Filename**	20241212-5.d
**ACQ Method**	ESI+R.m	**Comment**		**Acquired Time**	12/12/2024 12:31:16 PM (UTC+08:00)

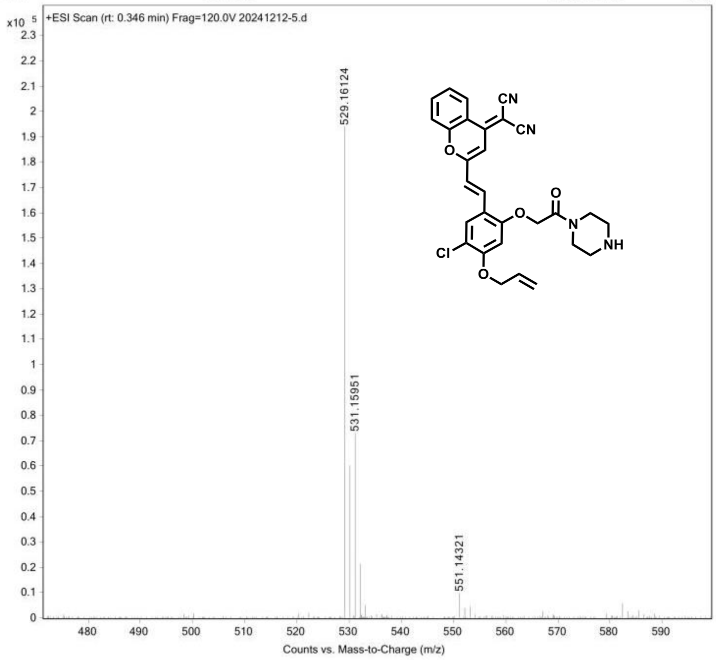

HRMS of Compound **6**

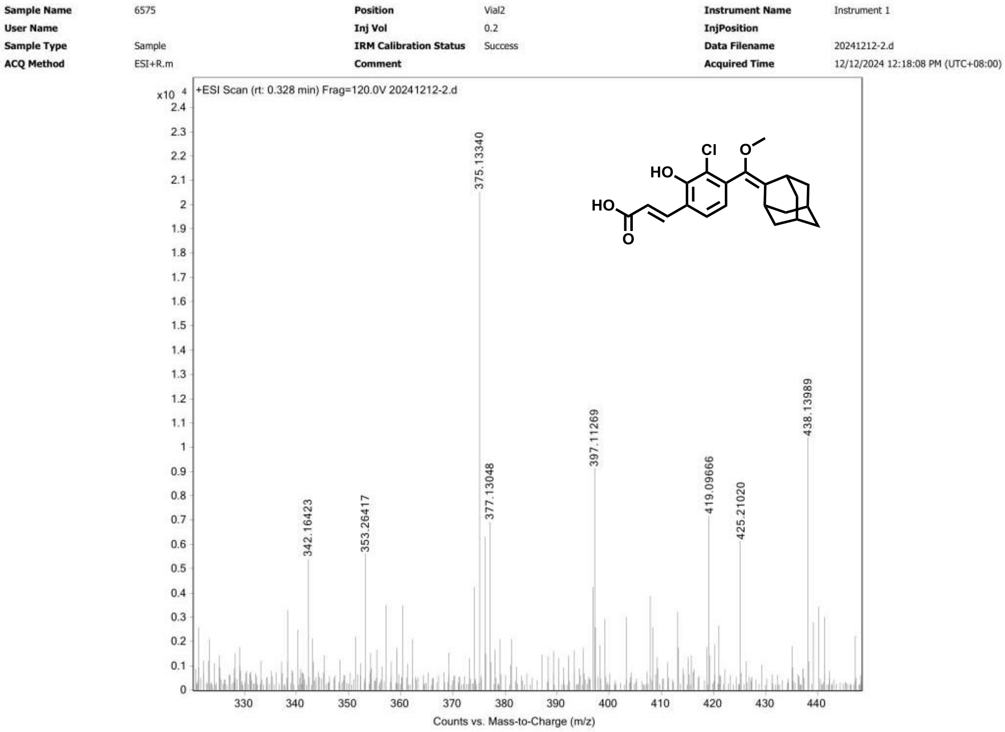

HRMS of Compound **7**

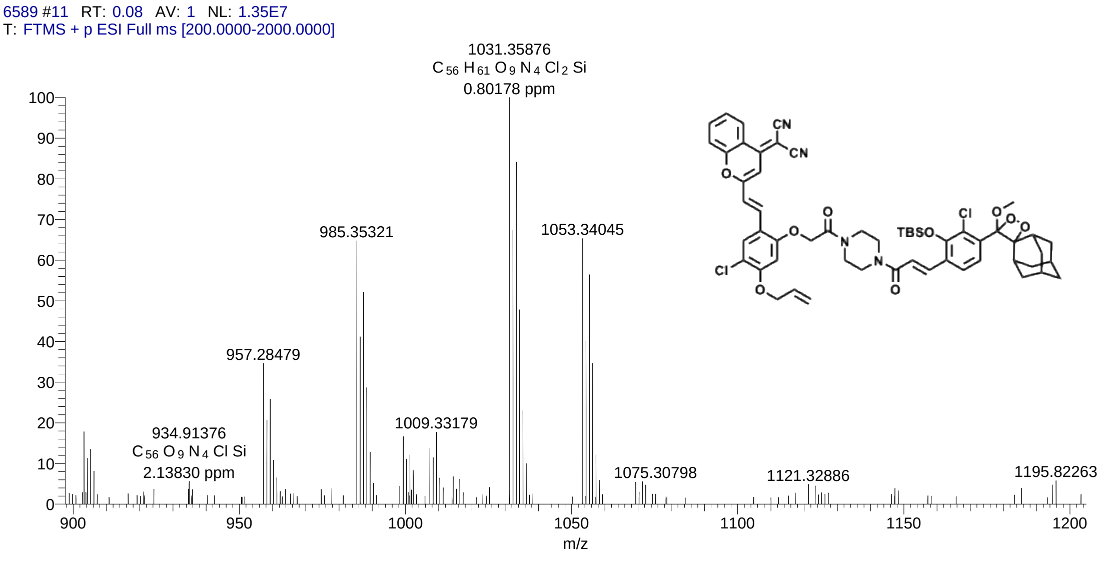

HRMS of **FTC-01**

**Sample Name**	6576	**Position**	Vial1	**Instrument Name**	Instrument 1
**User Name**		**Inj Vol**	1	**InjPosition**	
**Sample Type**	Sample	**IRM Calibration Status**	Success	**Data Filename**	(-)20241212-1.d
**ACQ Method**	ESI-R.m	**Comment**		**Acquired Time**	12/12/2024 2:20:09 PM (UTC+08:00)

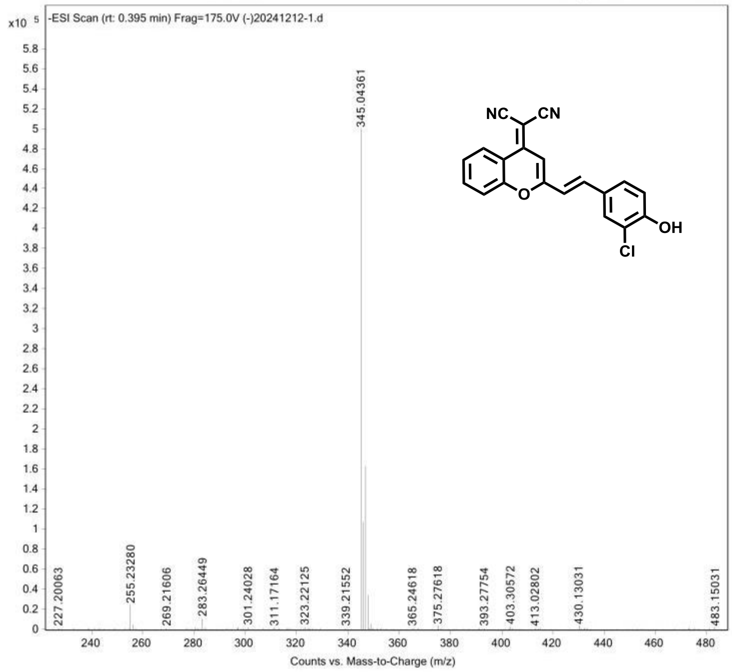

HRMS of Compound **F-01**

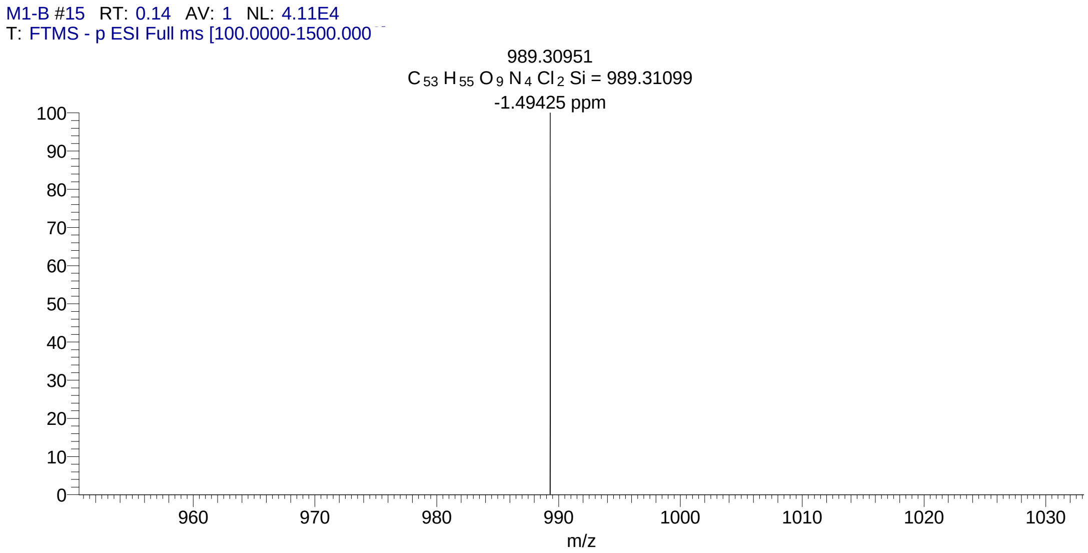

HRMS of Compound **M1**

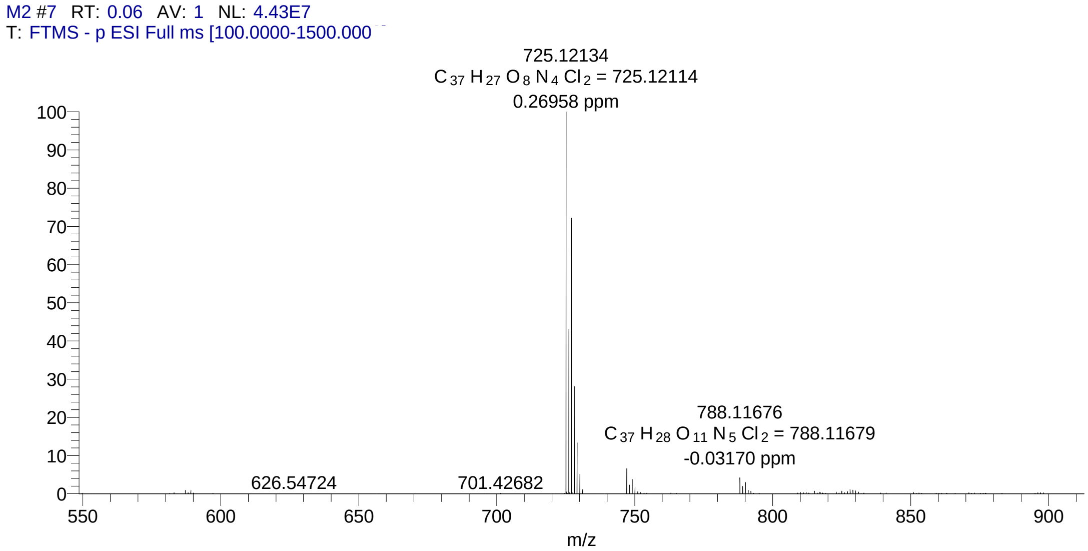

HRMS of Compound **M2**

REFERENCES

[1] Green, O.; Eilon, T.; Hananya, N.; Gutkin, S.; Bauer, C. R.; Shabat, D. Opening a Gateway for Chemiluminescence Cell Imaging: Distinctive Methodology for Design of Bright Chemiluminescent Dioxetane Probes. *ACS Cent. Sci.* **2017**, *3* (4), 349-358.

[2] Ryan, L. S.; Gerberich, J.; Haris, U.; Nguyen, D.; Mason, R. P.; Lippert, A. R. Ratiometric pH Imaging Using a 1,2-Dioxetane Chemiluminescence Resonance Energy Transfer Sensor in Live Animals. *ACS Sens.* **2020**, *5* (9), 2925-2932.

[3] Digby, E. M.; Tung, M. T.; Kagalwala, H. N.; Ryan, L. S.; Lippert, A. R.; Beharry, A. A. Dark Dynamic Therapy: Photosensitization without Light Excitation Using Chemiluminescence Resonance Energy Transfer in a Dioxetane–Erythrosin B Conjugate. *ACS Chem. Biol.* **2022**, *17* (5), 1082-1091.

[4] Chen, Z.; Su, L.; Wu, Y.; Liu, J.; Wu, R.; Li, Q.; Wang, C.; Liu, L.; Song, J. Design and synthesis of a small molecular NIR-II chemiluminescence probe for in vivo-activated H2S imaging. *Proc. Natl. Acad. Sci. U.S.A.* **2023**, *120* (8), e2205186120.

[5] Kagalwala, H. N.; Gerberich, J.; Smith, C. J.; Mason, R. P.; Lippert, A. R. Chemiluminescent 1,2-Dioxetane Iridium Complexes for Near-Infrared Oxygen Sensing. *Angew. Chem. Int. Ed.* **2022**, *61* (12), e202115704.

[6] Hananya, N.; Press, O.; Das, A.; Scomparin, A.; Satchi-Fainaro, R.; Sagi, I.; Shabat, D. Persistent Chemiluminescent Glow of Phenoxy-dioxetane Luminophore Enables Unique CRET-Based Detection of Proteases. *Chem. Eur. J.* **2019**, *25* (64), 14679-14687.

[7] Hananya, N.; Eldar Boock, A.; Bauer, C. R.; Satchi-Fainaro, R.; Shabat, D. Remarkable Enhancement of Chemiluminescent Signal by Dioxetane–Fluorophore Conjugates: Turn-ON Chemiluminescence Probes with Color Modulation for Sensing and Imaging. *J. Am. Chem. Soc.* **2016**, *138* (40), 13438-13446.
